# RNA profiling of blood platelets noninvasively differentiates colorectal cancer from healthy donors and noncancerous intestinal diseases: a retrospective cohort study

**DOI:** 10.1186/s13073-022-01033-x

**Published:** 2022-03-02

**Authors:** Luming Xu, Xinbo Li, Xiangchun Li, Xingyue Wang, Qian Ma, Dan She, Xiaohuan Lu, Jiao Zhang, Qianqian Yang, Shijun Lei, Lin Wang, Zheng Wang

**Affiliations:** 1 Department of Clinical Laboratory, Union Hospital, Tongji Medical College, Huazhong University of Science and Technology, Wuhan, 430022 China; 2 Research Center for Tissue Engineering and Regenerative Medicine, Union Hospital, Tongji Medical College, Huazhong University of Science and Technology, Wuhan, 430022 China; 3 Tianjin Cancer Institute, National Clinical Research Center of Cancer, Key Laboratory of Cancer Prevention and Therapy of Tianjin, Tianjin Medical University, Tianjin, 300060 China; 4 Department of Gastrointestinal Surgery, Union Hospital, Tongji Medical College, Huazhong University of Science and Technology, Wuhan, 430022 China

**Keywords:** Colorectal cancer, Early diagnosis, RNA profiles, Platelet

## Abstract

**Background:**

The RNA profiles of tumor-educated platelets (TEPs) possess pathological features that could be used for early cancer detection. However, the utility of TEP RNA profiling in detecting early colorectal cancer (CRC) versus noncancerous colorectal diseases has not yet been investigated. This study assesses the diagnostic capacity of TEP RNA profiles in a cohort of patients with CRC and noncancerous diseases.

**Methods:**

Transcriptome sequencing for platelets isolated from 132 patients with CRC at early and late stages and 190 controls consisting of healthy donors and patients with ulcerative disease, Crohn’s disease, polyps, and adenomas was performed and analyzed using binary particle swarm optimization coupled with support vector machine to identify genes that contributed to the classification of CRC patients versus controls. The area under the receiver operating curves (AUROCs) and the accuracy of TEP RNA profiles in CRC diagnosis were assessed.

**Results:**

TEP RNA profiling achieved high performance in distinguishing and staging CRC patients from the controls. Using the swarm intelligence algorithm, the 921 most contributive genes that classified CRC patients from the controls were identified. AUROCs of 0.928 for the training set via leave-one-out cross-validation and 0.92 for the validation set were achieved, both of which were significantly higher than the clinically utilized serum biomarkers: carcinoembryonic antigen and cancer antigen 19-9. Notably, an AUROC of 0.915 in an external validation set was achieved. For predicting different CRC stages, an AUROC of 0.984 was achieved in the training set and 1.000 in the internal validation set.

**Conclusions:**

RNA profiles of TEPs are of potential diagnostic value for identifying early CRC from noncancerous diseases. Prospective studies are needed to validate its clinical relevance.

**Supplementary Information:**

The online version contains supplementary material available at 10.1186/s13073-022-01033-x.

## Background

Colorectal cancer (CRC) is a leading cause of cancer-related death worldwide [1]. In the USA and China, the mortality and incidence of CRC rank 2nd/4th and 4th/3rd, respectively, among various cancers [2, 3]. Although surgical removal is infrequently curative once metastatic diseases occur, early CRC detection at surgically resectable stages without distant metastasis can indeed improve both survival outcomes and life quality of patients. Given that the development of CRC malignancy often takes two to three decades before clinical manifestation [4], a wide time window for detecting CRC before metastasis is provided. Therefore, it is critical to develop new methods and technology for the diagnosis of CRC at early stages, and particularly for patients who suffer from inflammatory bowel diseases (IBD, such as Crohn’s disease and ulcerative colitis), polyps, or adenomas, because these patients have a high risk of developing CRC [5–7]. Conventional serum protein biomarkers, such as carcinoembryonic antigen (CEA) and cancer antigen 19-9 (CA199), have low sensitivity and specificity. The sensitivity of CEA and CA199 to detect CRC was reportedly 46.6% and 14.4%, respectively, while the specificity was 80% and 89%, respectively [8]. Precancerous lesions, such as advanced adenoma and high-risk adenoma, also induce aberrant expression of CEA and CA199, which complicates early CRC detection [9]. Besides, some molecular biological markers of CRC, such as serum microRNAs and methylated septin-9 DNA, were also reported, but they did not distinguish well CRC from polyps or adenomas [10, 11]. Thus, a new accurate and efficient method is needed for screening CRC from patients with IBD, polyps, and adenomas.

Blood platelets, small anucleate cells originated from bone marrow megakaryocytes, play important roles in response to tumor progression. Apart from genetic materials, proteins, and RNAs derived from megakaryocytes, platelets also actively sequester proteins and spliced/unspliced RNAs, as well as oncogenic and angiogenic factors from cancer cells, such as VEGF, PDGF, and TGF-β [12]. Blood platelets reportedly contribute to creating tumor microenvironment supporting cancer development and progression [13]. The interplays between tumor cells and blood platelets impact tumor growth and dissemination [14–16]. Tumor cells can regulate blood platelets by transferring tumor-associated factors into platelets, consequently altering the expression profiles of blood platelets [17]. Meanwhile, platelet activation can release growth factors to facilitate tumor cell survival [18]. Platelets can encompass circulating tumor cells (CTCs) in the bloodstream, helping CTCs escape from immune cell-induced apoptosis and fluidic shearing force. Moreover, in addition to stimulating cancer cell adhesion and extravasation, platelets also contribute to CTC transmigration through the blood vessel walls and distant metastatic lesion formation [19].

RNA profiling of TEPs has emerged as a new liquid biopsy-based cancer detection method, allowing for noninvasive cancer detection [12]. The advantage of using RNA profiles of TEPs as a new strategy for early cancer detection is that platelet is the second most abundant cell types in the blood and can be stored at room temperature up to 48 h [20]. RNA profiles of TEPs were reported to achieve an AUROC of 0.99 and an accuracy of 96% in differentiating healthy donors and multiple cancer types at advanced or metastatic stages [20]. Of note, primary tumor sites and oncogenic alterations at the DNA level could be pinpointed using TEP RNA profiles [20]. The performance of using RNA profiles of TEPs to differentiate nonsmall cell lung cancer (NSCLC) from other noncancerous diseases was robust, but the control diseases were not closely related to NSCLC, and the classification accuracy was not as good as its performance in distinguishing healthy donors from cancer patients at advanced stages [21]. The accuracy of TEP-based detection of nonsmall cell lung cancer was reportedly 81% for early stages and 88% for late stages [21]. This performance is independent of age, smoking habits, and inflammatory states [21]. However, the analysis on RNA profiles of TEPs towards detecting early CRC, especially in the context of noncancerous intestinal diseases that were associated with the development of CRC, has not yet been explored. Tumorigenesis of sporadic CRC follows canonical multistep development, starting from polyps and adenomas to carcinoma and involving diverse genomic and epigenomic alterations [4], which collectively complicate early CRC detection. This study was set to assess the diagnostic performance of TEP RNA profiles in detecting CRC at an early stage from a cohort of 322 donors by expanding controls to cover a wide range of noncancerous diseases, such as IBDs, polyps, and adenomas.

## Methods

### Blood processing and platelet isolation

This study was conducted according to the Helsinki human subject doctrine and was approved by the Huazhong University of Science and Technology Review Board and Ethics Committee. Written consent to participate was acquired from all patients. A total of 322 blood samples were obtained from healthy donors (*n* = 21), patients with Crohn’s disease (CD, *n* = 40), ulcerative colitis (UC, *n* = 22), polyps (*n* = 48), and adenoma (Ad, *n* = 59) or CRC (*n* = 132) in Wuhan Union Hospital. The number of CRC patients at stages I, II, III, and IV was 25, 48, 58, and 1, respectively. Blood samples were stored in 5-mL purple-capped vacutainers equipped with the anti-coagulant EDTAK_2_ (purchased from Zhiyuan Medical Technology Co., Ltd.). Platelets were isolated using gradient centrifugation according to the standard experimental method described previously [22]. To evaluate platelet purity, morphological analysis was implemented to check freshly isolated and randomly selected platelet samples. Samples with 0–5 nucleated cells per 10 million platelets were included in the follow-up processes. Isolated platelets were lysed with 1 mL RNAiso (takara NO9109), followed by pipetting RNAiso solution to complete lysis. Platelet total RNA was purified with Direct-zol RNA Miniprep (ZYMO RESEARCH R2052), then 500 pg of total RNA was subjected to SMARTer mRNA amplification and sequencing. During blood acquisition for library preparation, 134 (29.4%) samples were excluded due to low blood volume (< 2 mL, six samples), nucleated cell contamination (60 samples), or poor RNA quality (total RNA < 5 ng and/or RIN value < 6, 68 samples).

### Transcriptome sequencing

The quality of total RNA was examined using an Agilent 2100 bioanalyzer and then was subjected to cDNA synthesis and amplification using SMARTer kit (Clontech Laboratories, Inc.) according to the manufacturers’ protocol. By using Agilent 2100 bioanalyzer with DNA high-sensitivity chip, we performed quality control to amplified sequencing. Samples were prepared using the Ovation® SoLo RNA-seq Systems (HUMAN PART NO0500) according to the manufacturer’s protocol*.* Finally, we pooled high-quality samples with product sizes ranging 300–500 bp in equimolar concentrations, then submitted for 100 bp paired-read sequencing on the Illumina HiSeq X-ten.

### Sequencing data analysis

We used STAR to perform spliced alignment of clean reads to human reference genome hg19 guided by annotated transcripts of Ensembl version 75 [23]. The intron-spanning read count table of each transcript was collected by STAR during sequence alignment. The read count table obtained from the STAR aligner was equal to those obtained from the HTSeq tool [24]. Both coding and noncoding RNAs were included in abundance estimation and downstream analyses.

### Differential gene expression analysis and data normalization

Differential gene expression analysis was performed using the R-package DESeq2 [25]. Transcripts with less than five read counts in all samples were excluded. We constructed DESeq object from the read count table obtained above and performed variance stabilizing transformation with the *vst* function [26]. Construction of the DESeq object included estimation of size factors, estimation of dispersion, and fitting negative binomial general linear model to the data. The differential expression analysis statistics including log2 fold-change, test statistics, and *p*-value were extracted from the DESeq object. We employed the R routine *results* to extract the result table from the DESeq analysis. We used the test statistics to perform gene set enrichment analysis for cancer hallmark pathways and platelet signatures downloaded from MsigDB [27]. We obtained normalized gene expression data after variance stabilizing transformation. We calculated the correlation of the expression level of each gene with the age of individuals. Genes that exhibited significant correlation with age were excluded from the downstream analysis to avoid the impact induced by age. We then performed surrogate variable analysis to remove unwanted variations within the data via the R-package sva [28].

### Training sets and validation sets

The training set (*n* = 202) was composed of 80 CRC patients at stages I (*n* = 15), II (*n* = 30), III (*n* = 34), and IV (*n* = 1) and 122 controls including HD (*n* = 17) and patients with CD (*n* = 24), UC (*n* = 24), polyps (*n* = 31), and Ad (*n* = 36) (Additional file 1: Table S1). The internal validation set (*n* = 120) was composed of 68 CRC patients at stages I (*n* = 10), II (*n* = 18), and III (*n* = 24) and 52 controls including HD (*n* = 4) and patients with CD (*n* = 16), UC (*n* = 8), polyps (*n* = 17), and Ad (*n* = 23) (Additional file 1: Table S1). Meanwhile, the cohort from Best and colleagues (*n* = 101, 38 cancer patients and 63 controls) was used as the external validation set [20].

### Feature selection via binary particle swarm optimization

Particle swarm optimization (PSO) mimics natural phenomena such as movements of bird flocks. The optimization procedure was first initiated with multiple particles. In the feature selection setting, particles are subsets of different predictors. Each particle has its position, velocity, and fitness value in the searching space. In our study, we used support vector machine (SVM) as the classifier and area under the receiver operating characteristic curve (AUROC) as the fitness value of the classification model. The fitness of the model was iteratively evaluated on the last position and current velocity, and the best position was determined. The PSO algorithm was firstly proposed for real-value optimization and later adapted to discrete optimization [29]. Let *x*_*id*_ and *v*_*id*_ denote the coordinates and velocity of the *i*_*th*_ particle in D-dimensional space and *g* as the index of the best particle in the neighborhoods identified so far [28]. The movement of the *i*_*th*_ particle is as follows:

$${v}_{id}={v}_{id}+\boldsymbol{\varphi} \left({p}_{id}-{x}_{id}\right)+\boldsymbol{\varphi} \left({p}_{gd}-{x}_{id}\right)$$

where *p*_*id*_ and *x*_*id*_ are binary values, i.e., 0 or 1, and *𝝋* is the random positive number generated for particle *i*_*th*_. The velocity vid was transformed by logistic function *S*(*v*_*id*_) as follows:

$$if\ \left(\mathit{\operatorname{rand}}\left(\right)<S\left({v}_{id}\right)\right)\ then\ {x}_{id}=1, else\ {x}_{id}=0$$

The function rand is a uniform random number generator in [0.0, 1.0], and the range of *S*(*vid*) is [0.0, 1.0]. We ran the algorithm for 100 iterations. The feature sets with the highest AUROC were used to build the final SVM classifier and subsequently evaluated its performance on internal and external validation sets. The R package caret (version 6.0–78) was used to build and optimize the parameters of the SVM classifier. The calculation of AUROC and the visualization of ROC were performed with the R package pROC (version 1.10.0). We used the R function multiclass.roc in the pROC package to calculate the multiclass AUROC, which implemented the multiclass AUROC calculation proposed by Hand and Till [30]. Tenfold cross-validation was used as an optimization cohort.

## Results

**Fig. 1:**
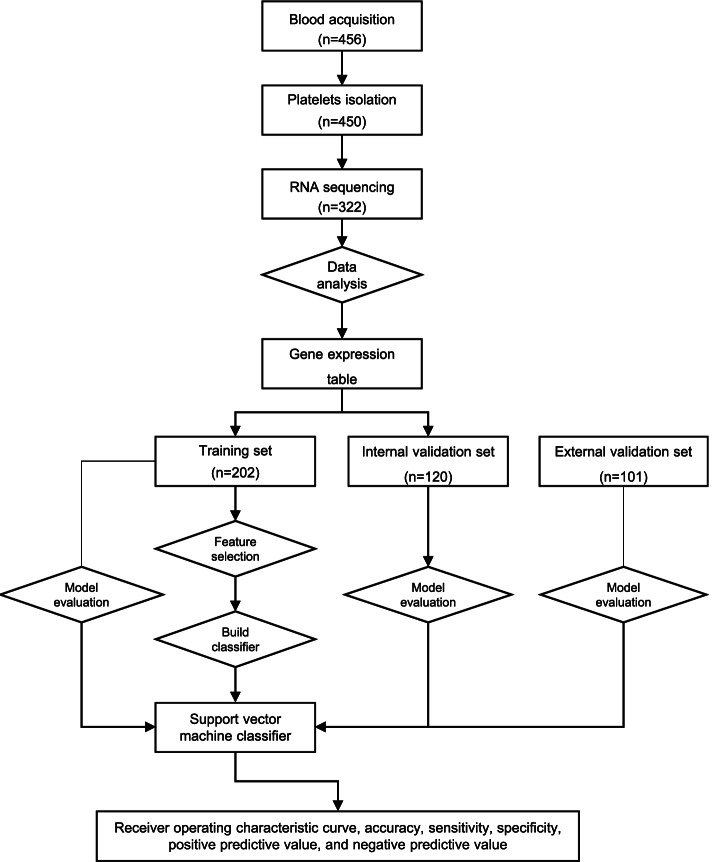
Flowchart depicting the experimental design of this study

**Fig. 2:**
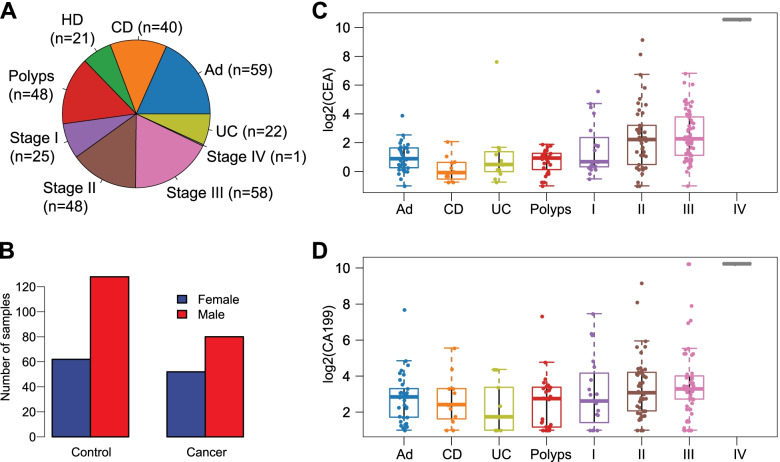
Distribution of blood platelet samples and serum levels of CEA and CA199 stratified by different diseases. **A** Numbers of different blood platelet samples from healthy donors (HD), patients with noncancerous diseases (polyps, Ad, UC, and CD), and CRCs (stages I–IV). **B** The number of males and females in the cancer group and control group. **C**, **D** Distribution of CEA (**C**) and CA199 (**D**) stratified by disease types and TNM stages (I, II, III, and IV)

We collected and isolated blood platelets from 132 CRC patients and 190 controls from Wuhan Union Hospital between January 2016 and August 2017 (322 samples in total). A flowchart depicting the experimental design is shown in Fig. 1. These 132 CRC patients included patients at stage I (*n* = 25), stage II (*n* = 48), stage III (*n* = 58), and stage IV (*n* = 1), whereas the control group included healthy donors (HD, *n* = 21) and patients with Crohn’s disease (CD, *n* = 40), ulcerative colitis (UC, *n* = 22), polyps (*n* = 48), and adenomas (Ad, *n* = 59) (Fig. 2A). The age ranged from 31 to 72 (mean ± SD, 54.6 ± 11.3) for healthy donors, 24 to 89 (59.3 ± 12.5) for CRC patients, 15 to 69 (30.6 ± 13.2) for CD, 18 to 65 (42.9 ± 11.9) for UC, 18 to 85 (56.2 ± 12.9) for polyps, and 30 to 76 (54.9 ± 10.1) for adenomas. The clinical features were provided in Additional file 1: Table S1. The proportions of different genders (i.e., male and female) in the CRC group versus the control group were comparable (60.6% male (80/132) versus 67.3% female (128/190); Fisher’s exact test, *p* = 0.237; Fig. 2B). The levels of CEA and CA199 were significantly higher in CRC patients versus the controls (Additional file 1: Fig. S1, log2-transformed median, 2.08 versus 0.77, 3.16 versus 2.68; Wilcoxon rank sum test, *p* < 0.001, *p* = 0.002, respectively). The distributions of CEA and CA199 with respect to disease status are shown in Figs. 1D and 2C.

**Fig. 3:**
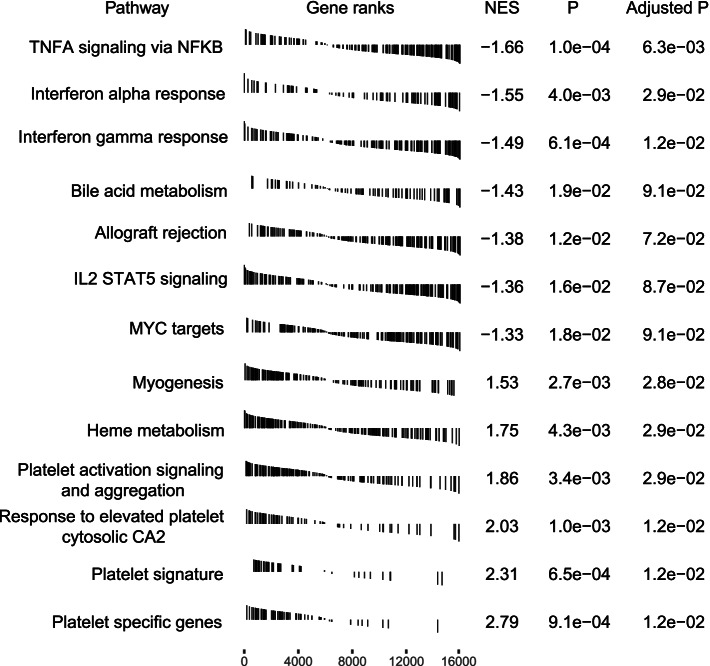
Gene set enrichment analyses of differentially expressed genes in the pathways of cancer hallmarks and platelet signatures

**Fig. 4:**
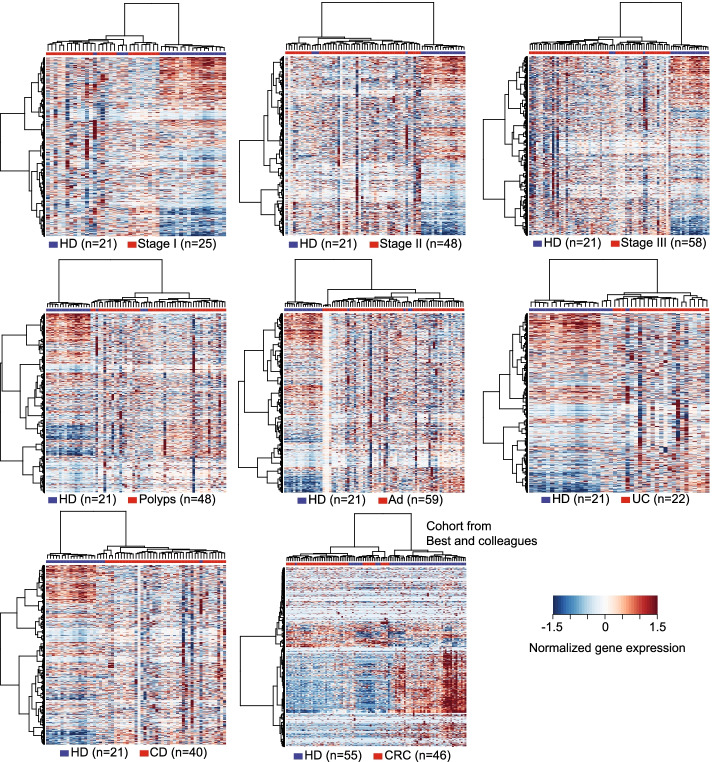
Heatmap representation of differentially expressed genes in healthy donor versus cancer group (stages I–III) and noncancerous controls (polyps, Ad, UC, and CD) in our cohort and in Best and colleagues’ cohort

We tested the platelet purity by morphological analysis (Additional file 1: Fig. S2) [20]. Platelet samples that passed the quality control criteria were subjected to RNA isolation, quality and quantity assessment, and RNA sequencing (see the “Methods” section). The median sequencing reads of 58.8 million per sample were obtained; RNA sequence alignment was performed by STAR [21], and the median percentage of uniquely mapped reads was 83.2% per sample (for detailed information regarding sequencing data and mapping results, see Additional file 1: Table S2). Intron-spanning reads of each gene were collected during sequence alignment (see the “Methods” section). After exclusion of genes with low coverage, 16,300 genes were finally obtained for the following analyses. We performed differential gene expression analysis and subsequently gene set enrichment analysis (GSEA). In total, 863 genes exhibited significant differences in CRC patients versus all controls (adjusted *p* < 0.1): 161 upregulated and 702 downregulated genes; 1095 genes exhibited differential expression across CRC patients, healthy donors, and patients with noncancerous diseases. The GSEA analysis showed that immune-related signaling circuits were significantly downregulated in CRC patients, whereas the circuits of platelet signatures and platelet activation were significantly upregulated in CRC patients. This finding was consistent with a previous study by Best and colleagues [20]. The downregulated circuits included TNF-α signaling via NF-κB, interferon α/γ responses, and IL2 signaling, while the upregulated circuits included myogenesis, heme metabolism, platelet signature, responses to elevated platelet cytosolic CA2, and platelet activation and aggregation (Fig. 3). On the contrary to CRC, those immune-related signaling pathways (TNF-α signaling via NF-κB, interferon α/γ responses, and allograft rejection) were significantly upregulated in both polyp or adenoma patients (Additional file 1: Fig. S3). The heatmap representation of differentially expressed genes (Fig. 4) suggests that RNA profiles of TEPs in healthy donors are clearly separable from patients diagnosed with CRC, polyps, adenoma, Crohn’s disease, and ulcerative colitis in our cohort (Fisher’s exact test, all *p* < 0.001) and in an external cohort from Best and colleagues (Fisher’s exact test, *p* < 0.001) [20].

**Fig. 5:**
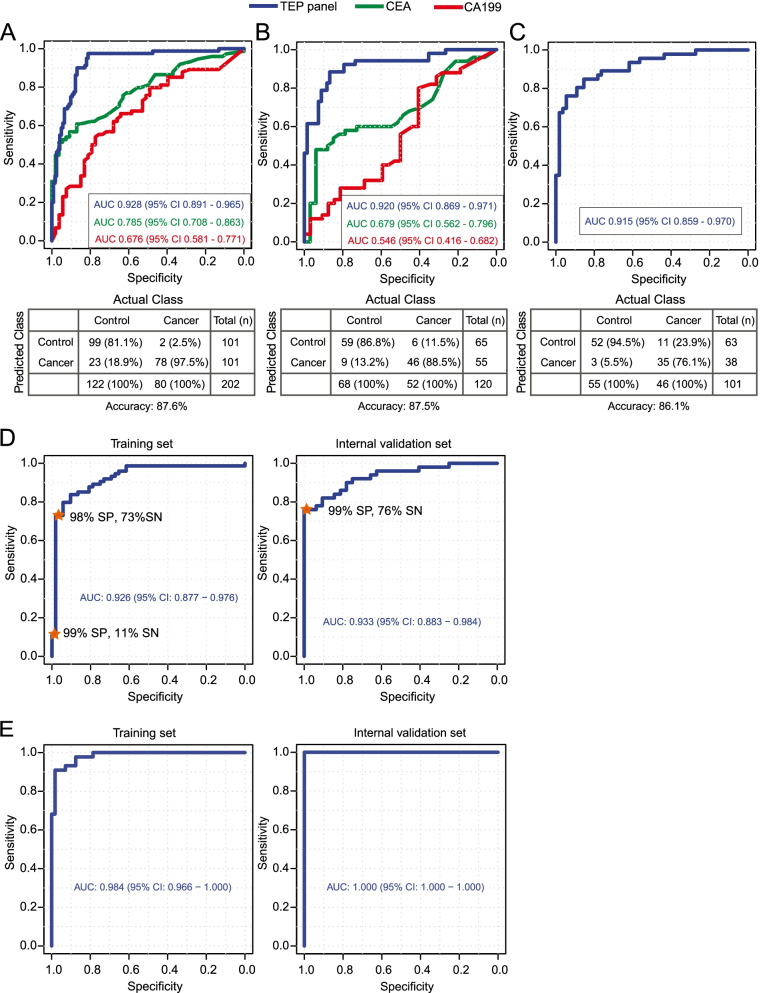
**A**–**C** The ROC curves of the selected gene panels and confusion matrices in the training set (**A**), the internal validation set (**B**), and the external validation set (**C**). **D** The ROC curves of the training set (via LOOCV) and the internal validation set by including CEA and CA199 into the set of selected genes. SP, specificity; SN, sensitivity. **E** The ROC curves of the selected genes in the classification of CRC stage

Binary particle swarm optimization (PSO) coupled with support vector machine (SVM) was used to identify a panel of genes that contributed the most to the classification of CRC patients versus controls. We excluded the genes significantly associated with age of individuals to avoid unwanted impact on classification. Variance stabilizing transformation of gene expression matrix (after exclusion of age-associated genes) was used as the inputs for binary PSO-based feature selection. Eventually, 921 genes were identified as the most contributive genes and used to build the cancer-versus-control classifier (Additional file 1: Table S3). The area under the receiver operating curves (AUROCs) for the repeated cross-validation of the training set and the internal validation set were evaluated iteratively (Additional file 1: Fig. S4), which indicates that SVM fits well to both the training and the validation sets. We achieved an AUROC of 0.928 (95% CI 0.891–0.965) on the training set (Fig. 5A) as measured by leave-one-out cross-validation and 0.92 (95% CI 0.869–0.971) on the internal validation set (Fig. 5B). Additionally, an AUROC of 0.915 (95% CI 0.859–0.970) was obtained for the external dataset reported by Best and colleagues (Fig. 5C) [20]. The AUROC values in the training set and the internal validation set were 0.785 (95% CI 0.708–0.863) and 0.679 (95% CI 0.562–0.796) for CEA, respectively, and 0.676 (95% CI 0.581–0.771) and 0.546 (95% CI 0.416–0.482) for CA199, respectively. The identified panel markers achieved significantly higher AUROC in both the training set and the internal validation set than did CEA (*p* = 0.001 and *p* = 0.0003, respectively) and CA199 (all *p* < 0.0001). For the training set, the classification accuracy, sensitivity, and specificity were 87.6%, 97.5%, and 81.1%, respectively; for the internal validation set, the classification accuracy, sensitivity, and specificity were 87.5%, 88.5%, and 86.8%, respectively; for the external dataset from Best and colleagues, the classification accuracy, the sensitivity, and the specificity were 86.1%, 76.1%, and 94.5%, respectively. Other classification metrics including positive predictive value, negative predictive value, kappa coefficient, and F1 score were shown in Table 1. Moreover, we observed that the classification accuracy was comparable when stratified by different disease types and TNM stages. The sensitivities of identifying CRC patients in the training set were 93.3% (14/15) at stage I, 96.7% (29/30) at stage II, and 100% (34/34) at stage III. For the internal validation set, the sensitivities were 80% (8/10) for stage I, 88.9% (16/18) for stage II, and 91.7% (22/24) for stage III. We achieved comparable classification performance when incorporating the serum levels of CEA and CA199 into the identified gene panels in both the training set (0.926, 95% CI 0.877–0.976) and the validation set (0.933, 95% CI 0.883–0.984) (Fig. 5D). The sensitivities were 73% in the training set at 98% specificity and 76% in the internal validation set at 99% specificity (Fig. 5D).

**Table 1:** Classification metrics of SVM

Performance metrics	The classification metrics of SVM across three data sets		
	Training set (LOOCV, *n* = 202)	Internal validation set (*n* = 120)	External validation set (*n* = 101)
Accuracy (95% CI)	0.876 (0.823–0.918)	0.875 (0.802–0.928)	0.861 (0.778–0.922)
Sensitivity (95% CI)	0.975 (0.913–0.997)	0.885 (0.766–0.956)	0.761 (0.612–0.874)
Specificity (95% CI)	0.811 (0.731–0.877)	0.868 (0.764–0.938)	0.945 (0.849–0.989)
Positive predicted value	0.772 (0.678–0.850)	0.836 (0.712–0.922)	0.921 (0.786–0.983)
Negative predicted value	0.980 (0.930–0.998)	0.908 (0.810–0.965)	0.825 (0.709–0.909)
Kappa^a^	0.752	0.747	0.717
F_1_^a^	0.862	0.860	0.833

^a^Kappa measured the agreement between the predicted classification with true labels. F_1_ was the harmonic average of precision (positive predicted value) and recall rates (sensitivity)

TEP RNA profiling also showed high sensitivity and specificity in predicting the stages of CRC. An AUROC of 0.984 (95% CI 0.966–1.000) was achieved on the training set and 1.000 (95% CI 1.000–1.000) for the internal validation set (Fig. 5E). Apart from CRC identification and staging, TEP RNA profiles also allowed the classification of healthy donors, patients with noncancerous diseases, and CRC patients. We obtained a gene panel of 929 genes from the binary PSO algorithm for the classification of healthy donors, patients with noncancerous diseases, and CRC (Additional file 1: Table S4). The AUROCs were 0.895 and 0.892 for the training set and the internal validation set, respectively (Additional file 1: Table S5).

## Discussion

Blood-based liquid biopsy provides a potential noninvasive alternative for early CRC detection. Among various liquid biopsy procedures (such as circulating cell-free DNA sequencing and gut microbiome profiling) [31–33], TEP RNA profiles emerged as a promising marker of molecular diagnostics to detect CRC at early stages. TEP RNA profiling has been employed for multiclass cancer diagnosis and reportedly achieved high accuracy in detecting several cancer types, including CRC [20]. However, previous studies did not include IBDs, polyps, and adenomas, which are common among human populations and often confound CRC early detection. Here, we showed that the TEP RNA profile can effectively detect CRC patients at an early stage from the population with the inclusion of noncancerous diseases, as well as predicting the stages of CRC. Our study revealed that RNA profiles of blood platelets from healthy donors are distinct from those of patients with CRC and other noncancerous diseases, whereas RNA profiles of blood platelets from patients with CRC and noncancerous diseases were admixed in the linear space of heatmap representation even though they were separable (Additional file 1: Fig. S5, Fisher’s exact test, *p* = 0.002). The SVM algorithm has high classification power as it projects data into higher dimensional space with kernel methods, which can model the nonlinear features embedded in the TEP RNA expression data that were not captured in heatmap representation. This underscored the importance of including noncancerous diseases in the control group when developing TEP-based CRC early detection methods. In the internal validation set, five patients with adenoma, two with polyps, and two with Crohn’s disease were misclassified as CRC (Fig. 5). Follow-up of these misclassified noncancerous patients in this study should be conducted to determine their risks of CRC development in the future.

Pathologically, there is a cross-talk between blood platelets and cancer cells. Cancer cells participate in platelet activation and reshape platelet RNA profiles by their oncogenic transformation mechanisms. Meanwhile, platelets contain growth and angiogenic factors facilitating cancer progression, and also interact with immune cells, such as natural killer cells and neutrophils, to promote cancer cell evasion from immune surveillance. Functional analysis from our study indicates that gene sets related to platelet activation and platelet signature are upregulated in CRC patients, while immune-related pathways, such as TNF-α signaling via NF-κB and interferon responses, are downregulated in CRC patients. This observation is consistent with a previous study [18]. Notably, the platelet RNA profiles of patients with polyps or adenomas are clearly distinguishable from healthy donors, which has not been reported before, suggesting that the development of noncancerous diseases involves pathological interactions with platelets.

The identified gene panels achieved significantly higher performance than did serum protein biomarkers, such as CEA and CA199 (*p* < 0.001). The performance was validated by an internal validation set and an external validation set whose control group however only contained healthy donors. The accuracy of detecting CRC patients at different stages was comparable. When incorporating CEA and CA199, the sensitivity of the performance of the classifier was marginally improved, suggesting that the selected gene panel possesses the CEA/CA199 comparable or even higher power in identifying CRC patients. Despite the incorporation of CEA and CA199, the performance of the classifier was marginally improved, suggesting that the selected gene panel possesses the CEA/CA199 comparable or even higher power in identifying CRC patients. Besides, TEP RNA profiling maintained a stable performance in the external validation set, indicating that the classifier is suitable for the data acquired by different RNA isolation and sequencing methods. Although some other biomolecules, such as cell-free DNA and intestinal microbiome, employed by several liquid biopsies were also interrogated in the early CRC detection, they have their own limitations. The recall rates based on mutations of cell-free DNA depend on the sequencing depth and vary across different stages [31]. The profiles of microbiome collected from fecal samples and intestinal microenvironment during colonoscopy examination were reportedly valuable for early CRC detection [32], but microbiome data are readily affected by antibiotics and sample collection procedures [32, 34, 35]. The classification power of these early CRC detection approaches, including our TEP RNA profiling, is expected to increase in the future by including more samples, incorporating multiple types of datasets, and using deep learning algorithms that have higher feature representation learning capability.

Although we demonstrated the applicability of TEP RNA profiling for CRC screening, some limitations should be considered. In this study, the sample exclusion rate reached 29.4%, mainly due to the insufficient blood volume, nucleated cell contamination, or poor sample quality, thus burdening the sample collection process. These underscore the importance of optimizing platelet and platelet RNA isolation procedure for TEP RNA profiling. Furthermore, the CRC diagnostic efficiency of TEP RNA profiling still needs to be evaluated in prospective studies.

## Conclusions

In summary, we showed that RNA profiles of blood platelet are potentially applicable for early CRC detection from noncancerous diseases. However, further validation, especially prospective validation, is required for further demonstrating the diagnostic significance of TEP RNA profiling.

## Supplementary Information

**Additional file 1: Fig. S1.** Distribution of CEA (left) and CA199 (right) in CRC patients and controls. **Fig. S2.** Representative images of isolated platelets (A) and bioanalyzer curves of platelet RNA (B). **Fig. S3.** Gene set enrichment analyses (GSEA) of differentially expressed genes in the blood platelets between patients with polyps or adenoma and healthy donors in hallmark gene sets from Molecular Signatures Database. **Fig. S4.** AUROCs of the training set (via repeated sampling) and the validation set at each iteration. **Fig. S5.** Heatmap representation of differentially expressed genes in control group versus cancer group. **Table S1.** Clinical features. **Table S2.** Alignment metrics. **Table S3.** Genes for classification of CRC patients from controls. **Table S4.** Genes for classification of CRC patients, healthy donors and patients with noncancerous diseases. **Table S5.** Predicted probabilities for multiclass classification.

**Additional file 2.** Raw reads count data matrix of all samples.

## Data Availability

The read count data matrix was submitted as Additional file 2. The raw sequence data were deposited in the Sequence Read Archive (SRA) in the National Center for Biotechnology Information (NCBI), under accession number PRJNA737596, that are publicly accessible https://www.ncbi.nlm.nih.gov/bioproject/PRJNA737596 [36]. The codes used in this study were deposited in GitHub https://github.com/lixiangchun/psofs [37].

## Abbreviations

Ad: Adenomas
AUROC: Area under the receiver operating curve
CA199: Cancer antigen 19-9
CD: Ulcerative disease
CEA: Carcinoembryonic antigen
CRC: Colorectal cancer
CTCs: Circulating tumor cells
GSEA: Gene set enrichment analysis
HD: Healthy donors
IBD: Inflammatory bowel disease
PSO: Particle swarm optimization
SVM: Support vector machine
TEP: Tumor-educated platelet
UC: Ulcerative disease
