# Supplementary material for: RNA profiling of blood platelets noninvasively differentiates colorectal cancer from healthy donors and noncancerous intestinal diseases: a retrospective cohort study
Source: Genome Med. 2022 Mar 2;14:26. doi: 10.1186/s13073-022-01033-x (PMC8889759; doi:10.1186/s13073-022-01033-x)
Supplement: Supplementary file 1 — Additional file 1: Fig. S1. Distribution of CEA (left) and CA199 (right) in CRC patients and controls. Fig. S2. Representative images of isolated platelets (A) and bioanalyzer curves of platelet RNA (B). Fig. S3. Gene set enrichment analyses (GSEA) of differentially expressed genes in the blood platelets between patients with polyps or adenoma and healthy donors in hallmark gene sets from Molecular Signatures Database. Fig. S4. AUROCs of the training set (via repeated sampling) and the validation set at each iteration. Fig. S5. Heatmap representation of differentially expressed genes in control group versus cancer group. Table S1. Clinical features. Table S2. Alignment metrics. Table S3. Genes for classification of CRC patients from controls. Table S4. Genes for classification of CRC patients, healthy donors and patients with noncancerous diseases. Table S5. Predicted probabilities for multiclass classification. [file 13073_2022_1033_MOESM1_ESM.pdf]

Additional Figures and Tables

Content:

Fig. S1. Distribution of CEA (left) and CA199 (right) in CRC patients and controls……………………………………………………………………..………..2

Fig. S2. Representative images of isolated platelets (A) and bioanalyzer curves of platelet RNA (B)……………………………………………………………...……3

Fig. S3. Gene set enrichment analyses (GSEA) of differentially expressed genes in the blood platelets between patients with polyps or adenoma and healthy donors in hallmark gene sets from Molecular Signatures Database…4

Fig. S4. AUROCs of the training set (via repeated sampling) and the validation set at each iteration…………………………………………………………….………5

Fig. S5. Heatmap representation of differentially expressed genes in control group versus cancer group………………………………………………………6

Table S1. Clinical features………………………………………………………7

Table S2. Alignment metrics…………………………………………………....15

Table S3. Genes for classification of CRC patients from controls……………..28

Table S4. Genes for classification of CRC patients, healthy donors and patients with noncancerous diseases……………………………………………………50

Table S5. Predicted probabilities for multiclass classification…………………73

Fig. S1 Distribution of CEA (left) and CA199 (right) in CRC patients and controls.

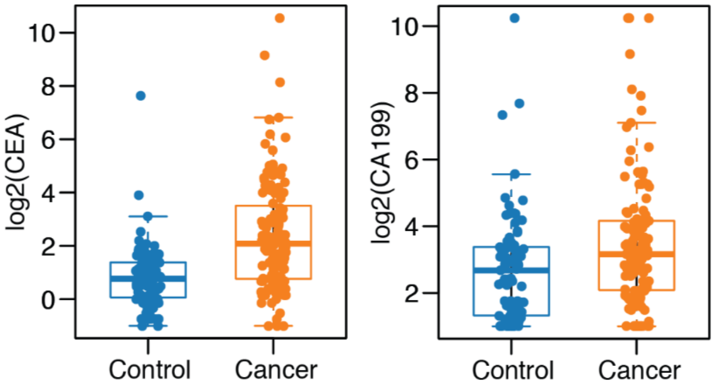

Fig. S2 Representative images of isolated platelets (A) and bioanalyzer curves of platelet RNA (B). Scale bars, 200 μm.

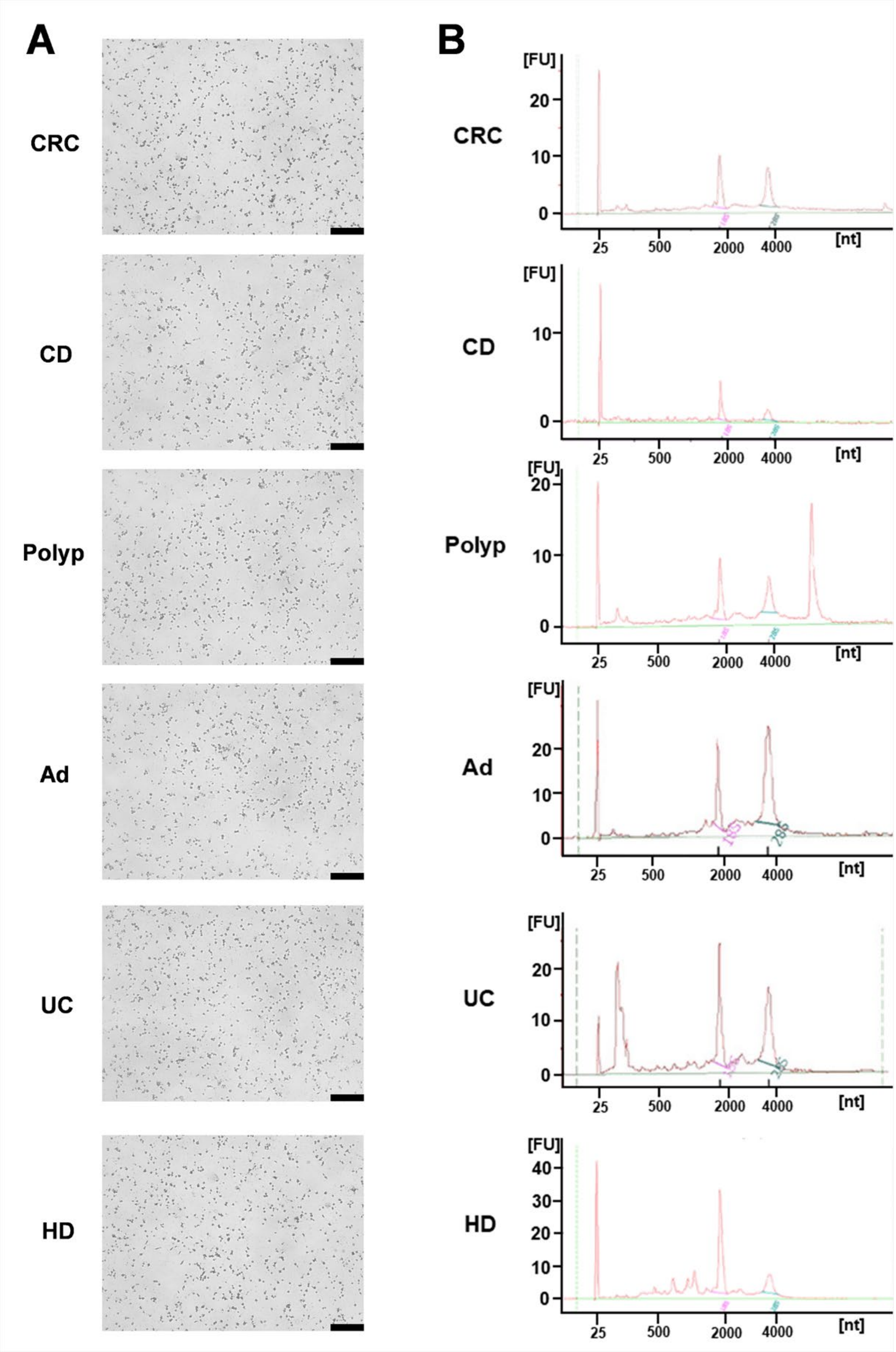

Fig. S3 Gene set enrichment analyses (GSEA) of differentially expressed genes in the blood platelets between patients with polyps or adenoma and healthy donors in hallmark gene sets from Molecular Signatures Database.

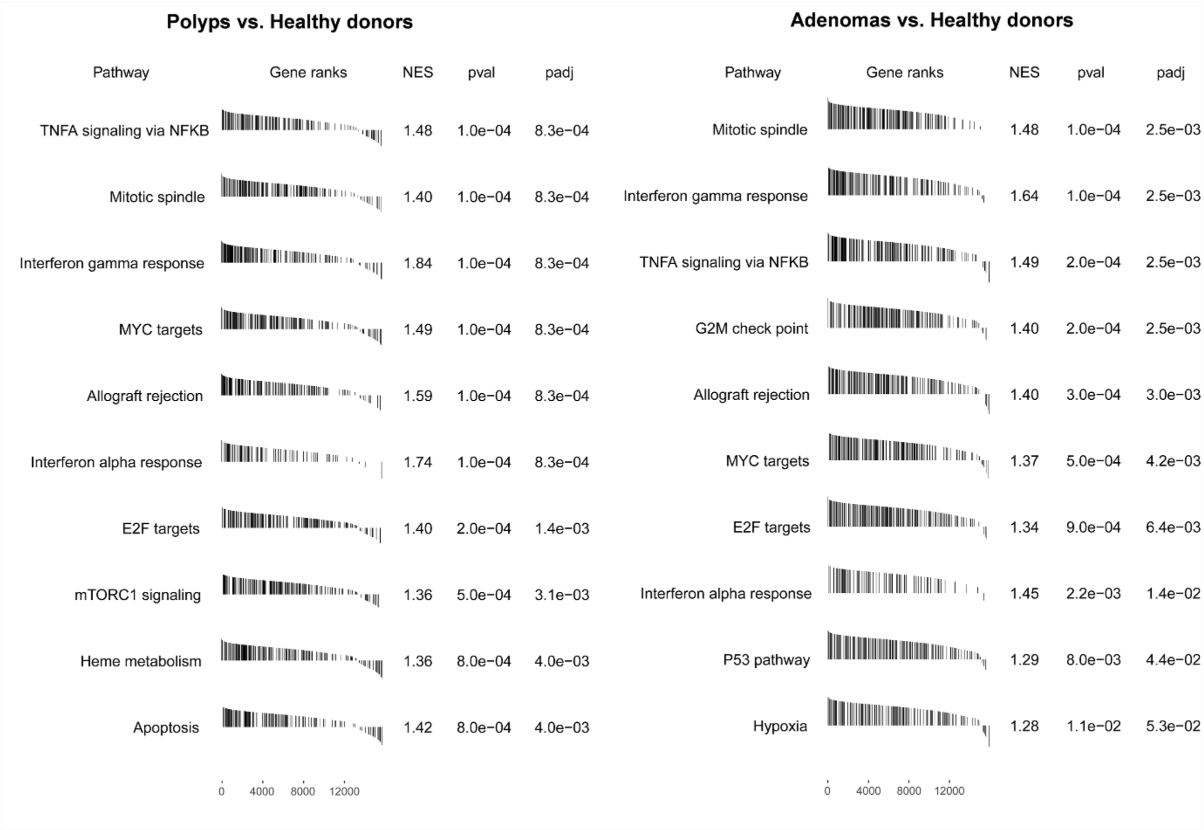

Fig. S4 AUROCs of the training set (via repeated sampling) and the validation set at each iteration.

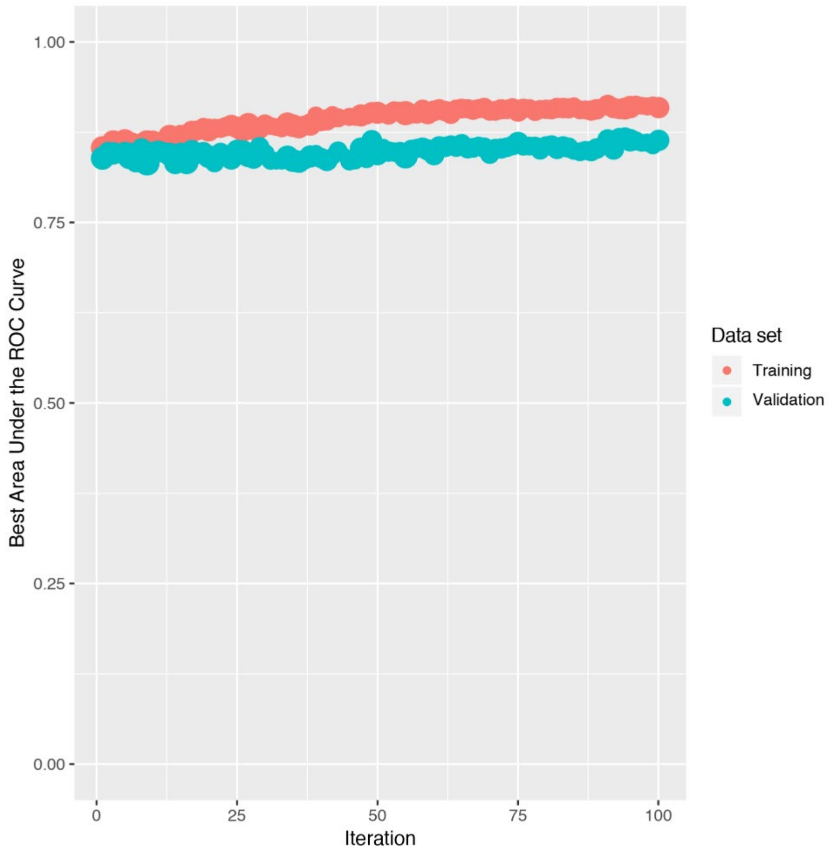

Fig. S5 Heatmap representation of differentially expressed genes in control group versus cancer group.

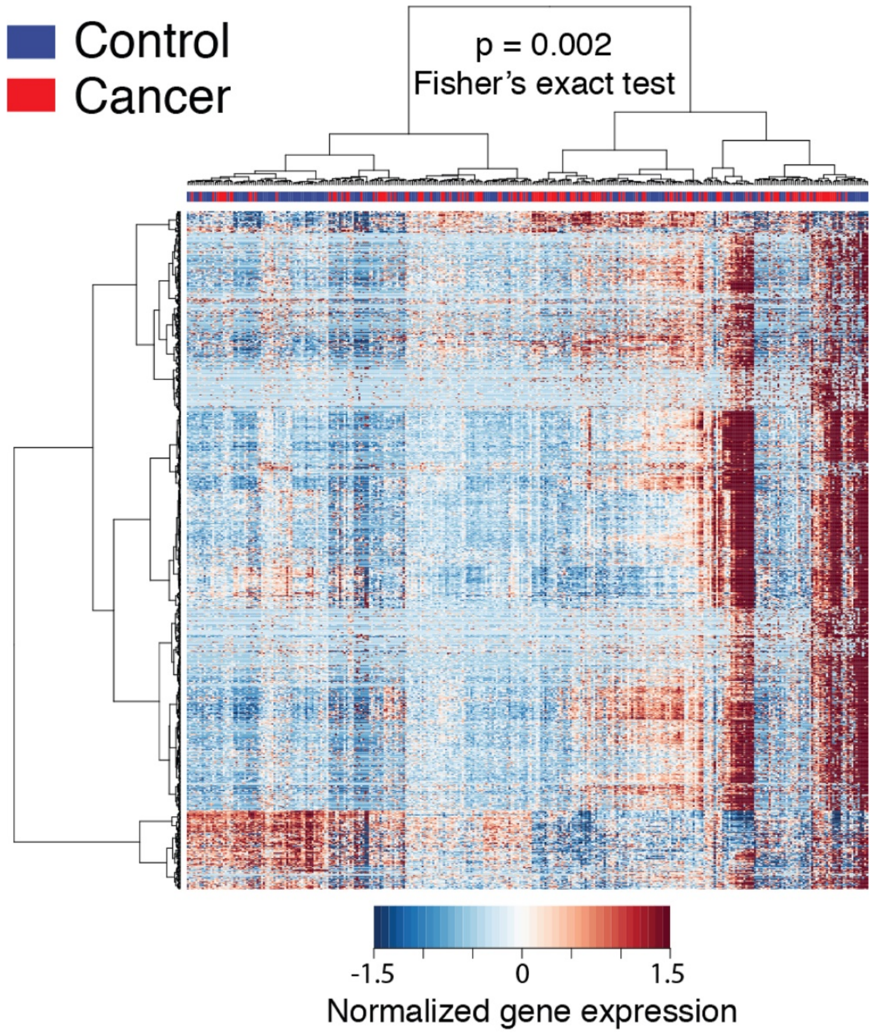

Table S1. Clinical features.

Sample ID	Group	CEA	CA199	Data Set
1390079A	ad	1.5	4.9	Training
1511894A	ad	NA	NA	Validation
1633021A	ad	5.8	206.3	Training
1641852A	ad	NA	NA	Training
1691282A	ad	NA	NA	Training
1706853A	ad	1.3	7.1	Validation
1750981A	ad	3.2	2.1	Training
1767539A	ad	3.3	7.6	Training
1818805A	ad	NA	NA	Training
1851168A	ad	NA	NA	Training
1853798A	ad	3.7	20.1	Training
1858985A	ad	NA	NA	Validation
1861715A	ad	2	5.3	Training
1865587A	ad	NA	NA	Validation
1865678C	ad	1.4	8	Training
1865727A	ad	NA	NA	Validation
1870332A	ad	NA	NA	Training
1875509A	ad	2.5	7.3	Validation
1878996A	ad	NA	NA	Validation
1879308B	ad	NA	NA	Training
1883356A	ad	NA	NA	Validation
1883708A	ad	NA	NA	Validation
1884008A	ad	4.6	24.6	Training
1884513A	ad	NA	NA	Validation
1884881A	ad	1.1	8.5	Training
1887168A	ad	2.3	28.9	Validation
1888448A	ad	NA	NA	Training
1889952C	ad	4	2.6	Training
1890001A	ad	3	7.3	Training
1890188A	ad	3.2	11.7	Training
1892655C	ad	1.1	6.4	Training
1894643C	ad	NA	NA	Training
1894735C	ad	1	14	Validation
1894774A	ad	1.8	4.7	Training
1897415A	ad	14.9	18.1	Validation
1897444A	ad	NA	NA	Training
1898490A	ad	NA	NA	Validation
1899157A	ad	0.5	2.4	Validation
1899623A	ad	NA	NA	Training
1899739A	ad	2.7	2.3	Training
1899845A	ad	3.1	8.6	Validation

Sample ID	Group	CEA	CA199	Data Set
1905833A	ad	1.8	8.4	Training
1906132A	ad	NA	NA	Validation
1906614A	ad	NA	NA	Training
1908768A	ad	1.9	9.9	Validation
1913593A	ad	0.7	2.8	Training
1914065A	ad	1.1	8.4	Validation
1915169A	ad	1.4	17.1	Training
1916287A	ad	NA	NA	Training
1923328A	ad	1.4	4.8	Validation
1923483A	ad	NA	NA	Training
1923712A	ad	1	3.3	Validation
1923760A	ad	NA	NA	Training
1928232A	ad	0.9	2.5	Training
1930827A	ad	1.7	3.4	Validation
3-19-1435611A	ad	1.9	2.2	Training
3-31-1930444A	ad	1.2	3.3	Training
4-10-1932654A	ad	2.6	2	Validation
4-8-1932227A	ad	NA	NA	Training
1437597A	crohn	NA	NA	Training
1510333A	crohn	NA	NA	Validation
1678848A	crohn	2.1	5.4	Validation
1697790A	crohn	1.6	5.3	Training
1737566C	crohn	NA	NA	Training
1740111A	crohn	NA	NA	Validation
1743552A	crohn	NA	NA	Training
1743786A	crohn	NA	NA	Training
1773040A	crohn	NA	NA	Training
1784113C	crohn	NA	NA	Training
1784113	crohn	NA	NA	Validation
1787667A	crohn	NA	NA	Training
1799829A	crohn	NA	NA	Training
1805451A	crohn	NA	NA	Validation
1808689A	crohn	NA	NA	Validation
1810051A	crohn	NA	NA	Validation
1817230A	crohn	NA	NA	Training
1822008A	crohn	NA	NA	Training
1831458A	crohn	NA	NA	Training
1849492A1	crohn	NA	NA	Validation
1849492A	crohn	NA	NA	Validation
1849694C	crohn	NA	NA	Validation
1857272A	crohn	1.2	2.8	Validation
1858555A	crohn	NA	NA	Validation

Sample ID	Group	CEA	CA199	Data Set
1865617A	crohn	0.8	2	Training
1865617C	crohn	0.8	2	Training
1869841B	crohn	NA	NA	Validation
1870205A	crohn	4.2	47.1	Training
1871427C	crohn	NA	NA	Training
1871480A	crohn	NA	NA	Validation
1874505A	crohn	1.5	8.4	Validation
1881219A	crohn	1	3.4	Training
1881790A1	crohn	0.6	9.9	Training
1881790A	crohn	0.6	9.9	Training
1884678A	crohn	NA	NA	Training
1886485A	crohn	0.9	4.6	Validation
1899597A	crohn	NA	NA	Training
1911217A	crohn	NA	NA	Training
1911731A	crohn	0.6	21	Training
1920204A	crohn	NA	NA	Training
1706470342A	hd	NA	NA	Validation
1706528242A	hd	NA	NA	Training
1706560342A	hd	NA	NA	Training
1897700A	hd	8.6	1200	Training
20180613-1A	hd	NA	NA	Training
20180613-3A	hd	NA	NA	Training
20180614-7A	hd	NA	NA	Training
20180614-9A	hd	NA	NA	Validation
20180619-12A	hd	NA	NA	Validation
20180619-8A	hd	NA	NA	Training
20180621-11A	hd	NA	NA	Training
20180621-16A	hd	NA	NA	Training
20180621-17A	hd	NA	NA	Training
20180621-18A	hd	NA	NA	Training
20180621-1A	hd	NA	NA	Training
20180621-20A	hd	NA	NA	Training
20180621-3A	hd	NA	NA	Training
20180621-4A	hd	NA	NA	Training
20180621-7A	hd	NA	NA	Training
20180621-8A	hd	NA	NA	Training
20180621-9A	hd	NA	NA	Validation
1521742A	polyp	NA	NA	Validation
1565660A	polyp	1.9	8.8	Training
1588154A	polyp	NA	NA	Training
1631136A	polyp	NA	NA	Training
1653398C	polyp	0.6	10.1	Training

Sample ID	Group	CEA	CA199	Data Set
1673045A	polyp	NA	NA	Training
1708311A	polyp	2.8	2.5	Training
1720296A	polyp	1	10.5	Training
1726513A	polyp	3.3	10.4	Validation
1745061A	polyp	2.6	9.3	Training
1746400A	polyp	NA	NA	Training
1759160A	polyp	1.8	2	Validation
1762308A	polyp	NA	NA	Training
1769697A	polyp	NA	NA	Training
1829253A	polyp	NA	NA	Training
1831610A	polyp	3.5	11.4	Validation
1859699A	polyp	2.8	12.6	Training
1880378B	polyp	NA	NA	Validation
1881428B	polyp	1.2	2.3	Training
1886064A	polyp	2.1	2	Validation
1888951A	polyp	NA	NA	Training
1889526A	polyp	NA	NA	Validation
1889576A	polyp	2.2	7.4	Training
1890357A	polyp	0.9	2	Training
1891753C	polyp	2.1	160.5	Validation
1893988A	polyp	NA	NA	Validation
1894005C	polyp	NA	NA	Training
1894926C	polyp	NA	NA	Training
1894946A	polyp	NA	NA	Training
1899012A	polyp	2.4	10.8	Training
1899447A	polyp	NA	NA	Training
1901282A	polyp	NA	NA	Training
1901448A	polyp	NA	NA	Validation
1904971A	polyp	1.9	2.3	Validation
1905067A	polyp	2.1	27.3	Validation
1905138A	polyp	1.5	2	Validation
1905350A	polyp	0.8	6.6	Training
1906391A	polyp	0.6	2.2	Training
1908348A	polyp	2.4	3.1	Training
1908351A	polyp	0.9	14.4	Training
1908612A	polyp	NA	NA	Training
1909623A	polyp	1.4	9.6	Validation
1917945A	polyp	3.7	2	Training
1923420A	polyp	1.6	2.7	Validation
1926306A	polyp	2	2	Validation
1928274A	polyp	NA	NA	Training
8125630A	polyp	0.5	6.9	Validation

Sample ID	Group	CEA	CA199	Data Set
8134785C	polyp	1.84	2.8	Training
1838221C	stage1	1.6	3.6	Validation
1840338A	stage1	NA	NA	Training
1841812A	stage1	26.3	28.6	Training
1846752A	stage1	47.8	176.8	Training
1853560A	stage1	1.1	3.7	Validation
1864082A	stage1	1.3	77.7	Validation
1874533A	stage1	0.9	2	Training
1877908C	stage1	22.2	2	Validation
1884726A	stage1	3.4	9.7	Training
1886703A	stage1	1.3	4.3	Validation
1887041A	stage1	2.8	2	Training
1891008C	stage1	0.7	6	Training
1892326C	stage1	1.4	8.1	Validation
1892923C	stage1	3.5	2	Training
1894753A	stage1	1.6	2	Training
1897877A	stage1	16.7	82.5	Training
1898496A	stage1	1.2	5.7	Validation
1902979A	stage1	NA	NA	Training
1904061A	stage1	1.3	23	Training
1905577A	stage1	1.1	7.9	Validation
1906588A	stage1	NA	NA	Validation
1907444A	stage1	NA	NA	Validation
1910771A	stage1	7.3	14	Training
1925846A	stage1	3.6	6.3	Training
4-2-1922690A	stage1	NA	NA	Training
1279536A	stage2	1.2	4.8	Training
1352617A	stage2	5.1	15.5	Validation
1561766A	stage2	3.2	12.6	Training
1654825A	stage2	6.4	4.2	Training
1824723A	stage2	0.6	16.5	Training
1830154A	stage2	107	574.2	Training
1860979A	stage2	56.8	20.1	Training
1864179A	stage2	5.2	3.2	Validation
1871082A	stage2	1.2	3.9	Validation
1876394B	stage2	4.4	17.8	Training
1880231B	stage2	2.2	18.7	Validation
1880284B	stage2	6.8	50.1	Training
1881406A	stage2	4.6	3.5	Training
1883814A	stage2	2.6	6.7	Validation
1884478A	stage2	9.2	21.5	Validation
1885869A	stage2	17	61.8	Training

Sample ID	Group	CEA	CA199	Data Set
1886923A	stage2	7.6	2	Training
1887311A	stage2	1.1	16.9	Training
1888087A	stage2	3	8.9	Training
1888328A	stage2	8	5.1	Validation
1889737A	stage2	0.9	2	Validation
1896461C	stage2	7.6	6.8	Validation
1897393A	stage2	27.2	6.1	Validation
1897847A	stage2	568.7	275	Training
1898529A	stage2	6.7	18.5	Training
1900924A	stage2	0.5	3	Training
1904585A	stage2	19.5	3.8	Training
1904904A	stage2	2.2	3	Training
1905470A	stage2	NA	NA	Validation
1905977A	stage2	1.2	21	Training
1913516A	stage2	1.4	3.5	Training
1918957A	stage2	7.1	22.1	Validation
1923555A	stage2	1.2	6.5	Training
1924049A	stage2	0.5	49.5	Training
1924478A	stage2	9.3	17.4	Validation
1925252A	stage2	5.1	3.4	Training
1926272A	stage2	33.5	2	Validation
1927301A	stage2	1.2	4.2	Training
1928219A	stage2	2.1	10.9	Validation
1929671A	stage2	1.1	6.8	Training
1932301A	stage2	283.8	40	Validation
1941919	stage2	2.6	12.3	Training
4-3-1927708A	stage2	25.2	8	Validation
8128721A	stage2	2.2	15.9	Training
8131850A	stage2	4.7	7	Training
8133808A	stage2	3.94	11.1	Training
8133904A	stage2	NA	NA	Training
8134049A	stage2	15.98	4.2	Validation
1486870A	stage3	7.6	5.1	Training
1758810A	stage3	1.7	6.6	Training
1776331A	stage3	1.7	2.8	Training
1835320A	stage3	112.6	137.1	Validation
1843877A	stage3	1.3	4.5	Training
1860881A	stage3	3.7	16.1	Training
1864085A	stage3	10.8	15.2	Validation
1867295A	stage3	3.9	2	Validation
1867559A	stage3	2.9	10.8	Training
1870541A	stage3	5.3	8.8	Training

Sample ID	Group	CEA	CA199	Data Set
1870933A	stage3	2.4	6.7	Training
1872105A	stage3	27.3	45.1	Training
1873326C	stage3	8.3	6.7	Training
1874019A	stage3	1.7	2.8	Training
1878930A	stage3	5.1	7.9	Training
1878930B	stage3	5.1	7.9	Validation
1880678A	stage3	1.9	9.8	Training
1880678B	stage3	1.9	9.8	Validation
1883324B	stage3	1	6.6	Training
1883327B	stage3	73.1	38.1	Training
1883444B	stage3	4.4	9.7	Training
1886737A	stage3	1.7	8.7	Training
1887113A	stage3	15.8	1200	Training
1888419A	stage3	1.6	12.4	Training
1889293	stage3	20.9	10.1	Training
1890150A	stage3	9.1	2	Training
1890561A	stage3	23.3	124.4	Validation
1893061C	stage3	5.3	38.2	Validation
1893507A	stage3	2.4	18	Training
1894272C	stage3	10.3	36.5	Validation
1894880A	stage3	29.1	1200	Training
1895869C	stage3	15.9	11	Training
1898696A	stage3	13.8	10.6	Training
1898763A	stage3	25.5	2	Validation
1898813A	stage3	18.3	2.2	Validation
1899032A	stage3	3.2	12.6	Validation
1899554A	stage3	2.7	11.8	Validation
1902019A	stage3	4.8	240.7	Training
1904783A	stage3	7.9	9	Validation
1904949A	stage3	67.2	9.2	Training
1905402A	stage3	NA	NA	Validation
1910156A	stage3	1.5	3.8	Training
1911215A	stage3	1.8	2.9	Validation
1911352A	stage3	6	11.1	Training
1912491A	stage3	4.1	15.3	Validation
1915523A	stage3	19.2	38.3	Training
1915766A	stage3	7.1	46.6	Validation
1926132A	stage3	32.1	1200	Training
1928455A	stage3	1.8	4.5	Validation
1929976A	stage3	4.4	7.1	Validation
1940746A	stage3	3.1	2	Validation
1947162	stage3	29.9	2	Validation

Sample ID	Group	CEA	CA199	Data Set
8128463A	stage3	2.18	17.4	Training
8129174A	stage3	2.4	6.8	Training
8132228A	stage3	2.29	14.4	Training
8133283A	stage3	0.5	6.8	Validation
8135483A	stage3	1.2	5.7	Validation
8136591A	stage3	11.97	10.9	Validation
1908698A	stage4	1500	1200	Training
1386860A	uc	NA	NA	Training
1488213A	uc	1.1	NA	Validation
1496601A	uc	NA	20.7	Training
1558998-A	uc	NA	NA	Training
1763793A	uc	1.1	NA	Training
1765515A	uc	1.7	2	Validation
1771180	uc	NA	NA	Training
1837489A	uc	NA	NA	Training
1844917B	uc	NA	NA	Validation
1858491A	uc	0.9	2	Training
1865925A	uc	0.6	5.1	Validation
1887010A	uc	NA	NA	Training
1890841A	uc	2.3	10.4	Validation
1892481C	uc	1.4	20.6	Training
1893092C	uc	0.7	3.4	Validation
1894201C	uc	NA	NA	Training
1895926A	uc	198.2	2	Validation
1897570A	uc	NA	NA	Validation
1902831A	uc	1.4	2	Training
1911263A	uc	NA	NA	Training
1911496A	uc	3.2	3.3	Training
1921957A	uc	2.9	NA	Training

Table S2. Alignment metrics.

Sample ID	Number of input reads	Uniquely mapped reads number	Uniquely mapped reads %	Average mapped length	Number of splices: Total	Number of splices: Annotated (sjdb)	Number of splices: GT/AG	Number of splices: GC/AG	Number of splices: AT/AC	Number of splices: Non-canonical	Mismatch rate per base, %
1279536A	60384187	51818300	85.81%	198.05	14810367	14020198	14468170	102292	9172	230733	0.44%
1352617A	52315249	41822921	79.94%	198.1	17675800	16881406	17368566	106800	11093	189341	0.39%
1386860A	60823724	48466995	79.68%	197.97	5158681	4370408	4565624	55370	5649	532038	0.58%
1390079A	50483431	40691365	80.60%	197.78	16158485	15478626	15852748	98210	10320	197207	0.44%
1437597A	55511689	49190182	88.61%	197.57	20607967	19483821	19996425	147795	13701	450046	0.41%
1486870A	50205476	43211425	86.07%	197.82	20053319	19205854	19631536	122876	11126	287781	0.39%
1488213A	55235657	43105912	78.04%	196.92	17860083	16943931	17323199	130898	13474	392512	0.43%
1496601A	56006574	43583561	77.82%	196.48	16155780	15034233	15503268	130891	12291	509330	0.77%
1510333A	49339271	43390696	87.94%	198.38	9674884	9048748	9248109	79401	7665	339709	0.42%
1511894A	51659946	45057109	87.22%	197.89	17536726	16685585	17190500	104120	10547	231559	0.39%
1521742A	60084867	50364650	83.82%	198.28	15561288	14791788	15159262	97325	9372	295329	0.50%
1558998-A	24967904	22101434	88.52%	198.2	2925494	2687956	2791200	23085	2467	108742	0.60%
1561766A	55091165	38763038	70.36%	196.27	14535102	13584621	13949704	96189	9355	479854	0.48%
1565660A	55621767	45773482	82.29%	196.72	16360971	15177201	15746382	127192	11252	476145	0.53%
1588154A	55301408	48912488	88.45%	197.91	11026252	10300264	10693806	83156	7565	241725	0.40%
1631136A	55496852	48968040	88.24%	197.9	14066393	12961989	13541868	116338	13599	394588	0.47%
1633021A	55617431	47286145	85.02%	196.77	19999610	18832372	19393576	138456	13642	453936	0.51%
1641852A	55287545	48419448	87.58%	197.95	13424982	12642207	13091104	93466	6846	233566	0.51%
1653398C	50435945	41343357	81.97%	197.55	13133132	12405917	12792862	84149	9132	246989	0.72%
1654825A	55269027	46946431	84.94%	198.06	23002372	22079220	22417938	162697	16794	404943	0.40%
1673045A	51942115	45649778	87.89%	198	21259109	20363034	20787825	137173	14086	320025	0.51%
1678848A	51130138	43330589	84.75%	197.84	17906604	17119713	17567279	106666	11216	221443	0.36%
1691282A	20030176	11908326	59.45%	197.02	1138058	736273	821524	11364	1258	303912	0.75%
1697790A	48987716	33299633	67.98%	196.65	12088823	11350208	11614805	83604	7286	383128	0.45%
1706470342A	55072844	38642074	70.17%	196.72	13552605	12864847	13233247	91104	8745	219509	0.44%

Sample ID	Number of input reads	Uniquely mapped reads number	Uniquely mapped reads %	Average mapped length	Number of splices: Total	Number of splices: Annotated (sjdb)	Number of splices: GT/AG	Number of splices: GC/AG	Number of splices: AT/AC	Number of splices: Non-canonical	Mismatch rate per base, %
1706528242A	55374796	29745142	53.72%	196.24	12760079	12043850	12408919	78256	8550	264354	0.47%
1706560342A	55312787	30643428	55.40%	196.68	9578923	9015517	9267782	64041	5895	241205	0.50%
1706853A	54961707	45434046	82.66%	197.69	16005854	15113544	15517914	116972	11370	359598	0.43%
1708311A	51867165	41878851	80.74%	197.45	18652578	17714146	18150272	134411	13111	354784	0.50%
1720296A	55589442	45110980	81.15%	198.05	18134467	17324126	17751381	112785	10036	260265	0.49%
1726513A	76124049	49459279	64.97%	197.09	16724314	15661164	16134836	118808	11257	459413	0.38%
1737566C	50568075	42371512	83.79%	198.09	15841005	15174082	15504921	103971	10774	221339	0.44%
1740111A	27920224	24145555	86.48%	198.28	4572748	4220399	4351769	38201	3452	179326	0.50%
1743552A	56783729	39919901	70.30%	196.83	13617139	12539744	12915372	102316	9577	589874	0.45%
1743786A	78516049	59088646	75.26%	197.14	19538900	18223810	18831232	149966	13128	544574	0.38%
1745061A	55427110	40663571	73.36%	198.59	12936625	12349698	12664455	85713	8827	177630	0.43%
1746400A	55573926	47950959	86.28%	198.02	15499328	14628780	15035948	111700	10822	340858	0.43%
1750981A	55684652	47659026	85.59%	198.16	16500007	15836830	16203483	96891	9822	189811	0.54%
1758810A	28245237	23410783	82.88%	197.35	6407039	5976108	6164378	49214	5407	188040	0.50%
1759160A	68062373	38559312	56.65%	196.57	10304787	9529334	9869494	91677	7064	336552	0.56%
1762308A	55442246	42074245	75.89%	196.87	15498815	14561472	14915535	104445	10513	468322	0.46%
1763793A	55409268	44944998	81.11%	197.38	21596277	20382825	20758507	168917	15608	653245	0.41%
1765515A	74636156	56736482	76.02%	197.53	10046594	8598893	8924145	122828	9712	989909	0.46%
1767539A	60363156	50237891	83.23%	197.69	15985420	15055084	15530776	113291	11429	329924	0.49%
1769697A	50045681	36589176	73.11%	198.55	13445266	12885979	13188717	82724	8198	165627	0.39%
1771180	111109657	73586982	66.23%	198.77	5138970	4309533	4385684	47113	4225	701948	0.46%
1773040A	50223319	43771824	87.15%	197.92	17308756	16474645	16952411	103965	12559	239821	0.34%
1776331A	55609145	44226408	79.53%	196.74	18106799	16971768	17418542	138769	13375	536113	0.50%
1784113C	50375715	41663570	82.71%	198.06	13826670	13091671	13437652	91302	9474	288242	0.50%
1784113	64967753	26065297	40.12%	198.56	3839888	3539020	3653379	29539	1782	155188	0.48%
1787667A	50543982	43237304	85.54%	197.79	16127104	15330862	15787421	97084	10944	231655	0.40%

Sample ID	Number of input reads	Uniquely mapped reads number	Uniquely mapped reads %	Average mapped length	Number of splices: Total	Number of splices: Annotated (sjdb)	Number of splices: GT/AG	Number of splices: GC/AG	Number of splices: AT/AC	Number of splices: Non-canonical	Mismatch rate per base, %
1799829A	50318541	42977792	85.41%	197.67	16926572	16092858	16612958	102597	9233	201784	0.44%
1805451A	50967081	44177781	86.68%	198.11	19384973	18569850	18998432	124016	13750	248775	0.42%
1808689A	55578309	45451741	81.78%	196.9	19624155	18564102	19073138	130978	12193	407846	0.45%
1810051A	26973486	19833475	73.53%	196.27	7059228	6589936	6811040	47626	4553	196009	0.47%
1817230A	50282963	43795812	87.10%	198.08	13276443	12524976	12900873	93295	10353	271922	0.41%
1818805A	55203336	47871608	86.72%	198.25	16659463	15823405	16266768	106581	10589	275525	0.41%
1822008A	55979695	48079977	85.89%	197.76	15153686	14343421	14811712	93327	9870	238777	0.42%
1824723A	48723892	37086910	76.12%	196.94	14358204	13572535	13986232	91481	11342	269149	0.47%
1829253A	55073416	48862416	88.72%	198.46	5508427	4405531	4823786	85232	7434	591975	0.51%
1830154A	55437679	47942585	86.48%	198.26	20151322	19334514	19706942	141834	12541	290005	0.46%
1831458A	50533985	44660921	88.38%	197.8	17949045	17077323	17551000	113582	12229	272234	0.39%
1831610A	50430255	39742144	78.81%	195.75	19196832	18133882	18525077	151877	14899	504979	0.46%
1835320A	47867276	35441899	74.04%	196.05	12234476	11359966	11699443	88376	8916	437741	0.50%
1837489A	50332068	42833100	85.10%	197.77	16805315	16009662	16426549	103054	9623	266089	0.43%
1838221C	51025937	42786624	83.85%	197.22	17458854	16561553	16991444	122678	12129	332603	0.41%
1840338A	60509619	45978035	75.98%	197.86	13337828	12551964	12808859	97440	9783	421746	0.56%
1841812A	70208113	55135601	78.53%	196.73	21743757	20453776	21055395	151363	15186	521813	0.47%
1843877A	27008679	19750674	73.13%	195.63	7600326	7068905	7296997	50774	5115	247440	0.51%
1844917B	53148860	27846832	52.39%	196.64	2300505	1027547	1279452	19079	2735	999239	0.84%
1846752A	68441700	52452928	76.64%	196.12	17819984	16554443	17139718	133950	13836	532480	0.45%
1849492A1	50277466	44068971	87.65%	198.14	17633556	16796257	17217557	119429	12882	283688	0.37%
1849492A	50956773	43737773	85.83%	197.83	19185402	18292511	18754169	125296	12741	293196	0.41%
1849694C	50372883	43034406	85.43%	197.61	13732037	12954934	13286932	100284	10215	334606	0.46%
1851168A	64947759	53274571	82.03%	197.17	20882930	19733383	20295474	156091	15816	415549	0.39%
1853560A	55479616	47214124	85.10%	196.79	19087933	17960065	18565430	126617	12539	383347	0.42%
1853798A	64751975	52601210	81.23%	197.01	16999204	15852016	16332369	126716	11834	528285	0.38%

Sample ID	Number of input reads	Uniquely mapped reads number	Uniquely mapped reads %	Average mapped length	Number of splices: Total	Number of splices: Annotated (sjdb)	Number of splices: GT/AG	Number of splices: GC/AG	Number of splices: AT/AC	Number of splices: Non-canonical	Mismatch rate per base, %
1857272A	50868632	43924407	86.35%	197.65	16762431	15878129	16397898	103636	10513	250384	0.43%
1858491A	60537322	45954062	75.91%	198.29	6022390	5352233	5545880	56498	5238	414774	0.55%
1858555A	58620448	44155158	75.32%	197.31	7040815	5846983	6098129	97113	8147	837426	0.48%
1858985A	55477419	43578970	78.55%	197.75	18212983	17374027	17703698	132935	13569	362781	0.46%
1859699A	67415028	50230829	74.51%	197.17	12838076	11218335	11592865	116513	10149	1118549	0.47%
1860881A	72120533	42020761	58.26%	197.51	6280283	5160993	5403380	92527	6607	777769	0.50%
1860979A	60482812	44271078	73.20%	198.11	8434542	7823002	8052624	63778	5642	312498	0.56%
1861715A	64649921	48526800	75.06%	197.96	7840317	6833930	7068405	96709	6960	668243	0.47%
1864082A	60658298	47062830	77.59%	198.1	16341159	15698908	16010806	97780	10976	221597	0.38%
1864085A	59549904	37497320	62.97%	198.12	9462472	8934702	9158145	61607	6615	236105	0.43%
1864179A	60431638	46583650	77.08%	198.09	6600144	5862809	6084769	63951	5608	445816	0.55%
1865587A	20993300	17615667	83.91%	198.2	2232512	1989640	2086424	18169	2024	125895	0.58%
1865617A	50748225	43268952	85.26%	197.76	19028998	18250364	18620774	119338	12062	276824	0.60%
1865617C	50335453	41876184	83.19%	198.33	18028583	17285945	17547565	129756	11393	339869	0.39%
1865678C	50446197	39658212	78.61%	197.43	12378391	11603649	11961704	85305	9176	322206	0.68%
1865727A	50150895	41400122	82.55%	197.69	17101299	16301896	16702233	112220	12649	274197	0.42%
1865925A	24360684	18059615	74.13%	197.53	3359126	2958126	3086864	27901	2743	241618	0.59%
1867295A	50580827	41803507	82.65%	197.4	16279996	15376507	15826361	113439	11209	328987	0.49%
1867559A	50394074	38633407	76.66%	198.05	17608294	16890505	17235458	115737	12313	244786	0.46%
1869841B	55687383	46414252	83.35%	198.57	12689556	12031288	12298417	82772	9020	299347	0.51%
1870205A	76899604	53790173	69.95%	197.57	9153431	7808049	8130078	112212	8503	902638	0.55%
1870332A	69329715	57707928	83.24%	197.83	8034696	6645548	6936715	126755	9556	961670	0.47%
1870541A	76136994	50037405	65.72%	196.95	8954770	7407643	7783640	118440	8918	1043772	0.51%
1870933A	28004455	17999847	64.27%	197.37	2637150	2078743	2210012	23897	2447	400794	0.70%
1871082A	50438417	36455638	72.28%	198.36	17144604	16436282	16772361	115500	13062	243681	0.37%
1871427C	50184814	42540616	84.77%	197.99	20602550	19794979	20172715	136695	14147	278993	0.40%

Sample ID	Number of input reads	Uniquely mapped reads number	Uniquely mapped reads %	Average mapped length	Number of splices: Total	Number of splices: Annotated (sjdb)	Number of splices: GT/AG	Number of splices: GC/AG	Number of splices: AT/AC	Number of splices: Non-canonical	Mismatch rate per base, %
1871480A	39426664	24946738	63.27%	197.2	3144370	2419626	2598281	31525	3601	510963	0.72%
1872105A	50049231	43795230	87.50%	198.02	15799300	15029609	15488112	96851	11212	203125	0.49%
1873326C	51407866	44581177	86.72%	197.69	17618803	16716031	17156007	121699	12855	328242	0.40%
1874019A	26871108	21329390	79.38%	198.29	2391488	2110457	2217960	22292	2066	149170	0.56%
1874505A	75133670	60351784	80.33%	197.18	10181773	8388752	8780740	145248	11717	1244068	0.54%
1874533A	69526806	56784471	81.67%	197.38	8754673	7223896	7556017	123103	8725	1066828	0.51%
1875509A	60566756	31822774	52.54%	196.65	2655892	1257610	1551553	37219	4377	1062743	0.82%
1876394B	60292278	32874874	54.53%	196.58	4176737	2952378	3221322	52149	5146	898120	1.12%
1877908C	50320031	36844861	73.22%	197.31	13517358	12901235	13211488	85584	8615	211671	0.70%
1878930A	50622720	44391185	87.69%	198.1	15046132	14284331	14622827	101028	9269	313008	0.37%
1878930B	60110336	39985585	66.52%	198.21	6327378	5795118	6033943	46405	4178	242852	0.52%
1878996A	50814612	42685545	84.00%	197.83	18078623	17170799	17710585	110382	11548	246108	0.38%
1879308B	45643990	36341685	79.62%	198.2	5264286	4572448	4786231	49616	4927	423512	0.60%
1880231B	38161846	18380804	48.17%	196.36	1637815	709317	889723	12740	2080	733272	0.95%
1880284B	32847998	25498579	77.63%	198.1	3614310	3223887	3350952	30487	3145	229726	0.62%
1880378B	59988442	27009969	45.03%	196.1	3794845	2176344	2500117	38705	4581	1251442	0.89%
1880678A	50724958	33259412	65.57%	197.54	16515404	15761280	16085526	120213	11504	298161	0.37%
1880678B	50510104	38050983	75.33%	198.05	14985625	14271528	14570691	105254	11576	298104	0.40%
1881219A	20903284	14854097	71.06%	197.66	1715000	1367959	1467593	13828	1629	231950	0.61%
1881406A	50549489	43076331	85.22%	197.46	15986129	15066136	15626944	102424	10974	245787	0.49%
1881428B	26339141	22552987	85.63%	198.4	2250060	1967073	2081104	20054	2399	146503	0.58%
1881790A1	50389457	43407555	86.14%	198	15695342	14901688	15320381	107222	12564	255175	0.42%
1881790A	50441372	44104013	87.44%	198.22	14160978	13432447	13824007	93495	9752	233724	0.47%
1883324B	37658332	32477891	86.24%	198.04	4531629	4065780	4253311	39699	3784	234835	0.56%
1883327B	50183990	39702977	79.11%	197.58	6533113	5748185	5984688	58785	5926	483714	0.58%
1883356A	27839970	15347779	55.13%	197.11	2277716	1743602	1863502	19978	2300	391936	0.74%

Sample ID	Number of input reads	Uniquely mapped reads number	Uniquely mapped reads %	Average mapped length	Number of splices: Total	Number of splices: Annotated (sjdb)	Number of splices: GT/AG	Number of splices: GC/AG	Number of splices: AT/AC	Number of splices: Non-canonical	Mismatch rate per base, %
1883444B	60146861	29438064	48.94%	196.38	3592880	2012978	2322816	47019	4995	1218050	0.85%
1883708A	21519376	17745658	82.46%	198.01	3150047	2869246	2972491	26654	2822	148080	0.59%
1883814A	50973691	44617123	87.53%	197.91	18852534	18001714	18459674	113246	11673	267941	0.46%
1884008A	32488762	16854398	51.88%	196.48	1579505	775385	926808	16601	2532	633564	0.84%
1884478A	36725761	30323219	82.57%	198.02	5376804	4945096	5109112	44438	3805	219449	0.55%
1884513A	37054014	14571675	39.33%	195.22	1878160	704093	919096	15699	2864	940501	1.06%
1884678A	60155214	51177691	85.08%	197.99	16543767	15635448	16134836	108213	9992	290726	0.46%
1884726A	51221228	25327628	49.45%	196.39	3433108	2058663	2328660	48916	4967	1050565	0.81%
1884881A	50207502	43795153	87.23%	197.92	16479686	15629214	16114770	105683	10778	248455	0.41%
1885869A	50196751	42666939	85.00%	198.15	23554609	22745421	23038741	157237	16232	342399	0.38%
1886064A	50177732	43031108	85.76%	197.55	15750103	14829343	15395121	93880	9983	251119	0.34%
1886485A	27533118	23966880	87.05%	198.28	3469801	3165962	3270578	31100	3156	164967	0.44%
1886703A	60023669	49025002	81.68%	198.04	11344799	10438946	10794167	86868	8763	455001	0.50%
1886737A	50536867	43084237	85.25%	197.63	15341002	14478883	14967628	102140	10289	260945	0.38%
1886923A	50569593	43927687	86.87%	197.98	19315423	18520729	18994961	106478	10397	203587	0.36%
1887010A	50576614	39075233	77.26%	198.1	18330389	17583616	18003872	110416	12554	203547	0.43%
1887041A	50039041	43899635	87.73%	198.11	19033182	18222419	18706731	108261	12797	205393	0.45%
1887113A	50712619	44084785	86.93%	197.87	20844618	19967542	20402727	128135	13832	299924	0.42%
1887168A	50546184	42567049	84.21%	197.66	18334976	17417405	17883832	122211	12788	316145	0.41%
1887311A	50724663	43779222	86.31%	197.88	19550516	18688944	19102742	125050	13407	309317	0.39%
1888087A	50912098	44219610	86.85%	197.76	19258634	18322843	18843045	122702	13684	279203	0.44%
1888328A	50535753	43878017	86.83%	197.81	16733271	15891596	16370064	105964	11364	245879	0.45%
1888419A	32264981	28238029	87.52%	198.16	4998927	4660291	4773544	40868	3653	180862	0.66%
1888448A	50947942	45293759	88.90%	197.95	16995085	16100628	16609423	105539	12096	268027	0.44%
1888951A	51909385	44160408	85.07%	198.02	17146530	16318084	16816434	103191	10695	216210	0.46%
1889293	50054541	40619009	81.15%	197.78	14734571	14085056	14450203	87774	8986	187608	0.71%

Sample ID	Number of input reads	Uniquely mapped reads number	Uniquely mapped reads %	Average mapped length	Number of splices: Total	Number of splices: Annotated (sjdb)	Number of splices: GT/AG	Number of splices: GC/AG	Number of splices: AT/AC	Number of splices: Non-canonical	Mismatch rate per base, %
1889526A	50903873	43778603	86.00%	197.7	18077907	17176963	17697556	108666	12569	259116	0.46%
1889576A	50320899	43847115	87.13%	197.54	15724780	14815564	15396204	96253	10129	222194	0.43%
1889737A	55575037	44702525	80.44%	196.75	19461836	18346255	18832126	154315	15097	460298	0.47%
1889952C	50344112	39490269	78.44%	197.34	12422017	11826661	12119411	76168	8479	217959	0.70%
1890001A	24135051	20907612	86.63%	198.15	3324923	3050254	3180126	26039	2576	116182	0.54%
1890150A	50605752	43491898	85.94%	197.97	18542836	17747543	18157052	115089	12481	258214	0.39%
1890188A	50345973	42930795	85.27%	197.63	16037862	15197992	15668481	96903	9822	262656	0.46%
1890357A	50391696	42101479	83.55%	197.67	16308263	15452268	15911346	104859	11805	280253	0.45%
1890561A	60292460	48849060	81.02%	198.14	11621321	10884603	11237129	83456	7592	293144	0.52%
1890841A	57745655	51812307	89.73%	198.25	9897562	9064805	9449919	80823	7358	359462	0.49%
1891008C	50467871	39055359	77.39%	198.07	14834660	14136042	14487479	101380	9419	236382	0.41%
1891753C	50256723	40341993	80.27%	198.03	16429143	15711787	16039693	108950	10689	269811	0.42%
1892326C	50261761	40185217	79.95%	197.87	15898358	15167470	15497048	107971	10606	282733	0.42%
1892481C	50242277	41057165	81.72%	197.45	13007242	12305033	12646247	87912	8726	264357	0.89%
1892655C	45866838	35768435	77.98%	197.21	11908695	11163923	11466472	91206	8767	342250	0.43%
1892923C	50134663	42377668	84.53%	198.04	16947594	16177845	16567843	114546	10776	254429	0.42%
1893061C	50343852	41176521	81.79%	197.98	17925661	17137583	17438929	130752	13167	342813	0.39%
1893092C	50497272	41924852	83.02%	197.78	11029723	10409710	10746311	70583	7932	204897	0.82%
1893507A	8850666	7511828	84.87%	197.83	3491052	3340367	3408896	24655	2460	55041	0.43%
1893988A	55259450	44088731	79.78%	196.81	13754922	12751400	13223662	107561	9194	414505	0.46%
1894005C	50463967	38643355	76.58%	197.8	14851089	14193339	14510983	92520	10193	237393	0.43%
1894201C	50361823	41034559	81.48%	196.97	14384170	13527458	13930295	94147	9164	350564	0.48%
1894272C	50423233	41636658	82.57%	198.16	10396443	9793461	10091475	72849	6528	225591	0.44%
1894643C	50180815	42509669	84.71%	197.68	13212313	12510970	12812915	92872	9263	297263	0.76%
1894735C	50548190	40974071	81.06%	198.06	13421997	12688054	13009364	99012	10529	303092	0.41%
1894753A	57608530	51507655	89.41%	197.96	8493392	7732447	8061377	64537	7346	360132	0.48%

Sample ID	Number of input reads	Uniquely mapped reads number	Uniquely mapped reads %	Average mapped length	Number of splices: Total	Number of splices: Annotated (sjdb)	Number of splices: GT/AG	Number of splices: GC/AG	Number of splices: AT/AC	Number of splices: Non-canonical	Mismatch rate per base, %
1894774A	28406481	25075691	88.27%	198.13	6473209	6066941	6219396	50935	5095	197783	0.40%
1894880A	33421706	27649497	82.73%	198.15	5310620	4898292	5031679	44798	4736	229407	0.51%
1894926C	50192908	40780936	81.25%	197.85	15281004	14535829	14886333	102306	10070	282295	0.41%
1894946A	50568371	42304263	83.66%	197.59	20405500	19500709	19946708	135541	15377	307874	0.40%
1895869C	50330302	43210878	85.85%	197.99	14864005	14090601	14440650	106091	10086	307178	0.44%
1895926A	41968425	34244158	81.60%	197.75	13129862	12484298	12787106	84846	7779	250131	0.44%
1896461C	50407085	40312739	79.97%	197.56	14992290	14255088	14569681	104768	8857	308984	0.74%
1897393A	60050491	53601436	89.26%	197.97	9817307	9038928	9332609	83388	8487	392823	0.54%
1897415A	25595453	21971587	85.84%	197.56	9651604	9132744	9268758	83394	7548	291904	0.54%
1897444A	22166996	18943070	85.46%	197.73	2034452	1798499	1875022	20042	2147	137241	0.43%
1897570A	50379461	41993229	83.35%	197.63	19103506	18237765	18599564	139867	13466	350609	0.41%
1897700A	24680603	20222916	81.94%	198.32	3756761	3504299	3590813	29643	2326	133979	0.58%
1897847A	50533988	37065696	73.35%	197.98	15514801	14870960	15259410	89657	9639	156095	0.46%
1897877A	50690211	41187620	81.25%	197.16	20335340	19499454	19947717	130093	12192	245338	0.40%
1898490A	26563021	23045271	86.76%	198.12	2948252	2623215	2734232	26288	2880	184852	0.52%
1898496A	29499496	23543953	79.81%	197.83	3903089	3631054	3732684	29930	2829	137646	0.76%
1898529A	29574850	26308932	88.96%	197.92	4814396	4499306	4613138	37448	3672	160138	0.73%
1898696A	50496501	37773708	74.80%	197.94	18214910	17521625	17849127	116320	11677	237786	0.40%
1898763A	50314696	41743559	82.96%	198.05	15773030	15043364	15349033	110946	10753	302298	0.46%
1898813A	47527165	42463365	89.35%	198.55	5481175	5088994	5248225	39997	4737	188216	0.51%
1899012A	50186189	37662335	75.05%	198.43	16574074	15924382	16245966	111003	11506	205599	0.37%
1899032A	50182820	42867914	85.42%	197.84	17471234	16605284	17024428	127723	12581	306502	0.40%
1899157A	50612382	42918409	84.80%	198.2	16861953	16143312	16488533	109238	10619	253563	0.45%
1899447A	50749570	34317573	67.62%	197.94	11907238	11325531	11608949	78512	9063	210714	0.40%
1899554A	50343158	41346720	82.13%	197.5	17992894	17129904	17506198	130865	13744	342087	0.43%
1899597A	55766835	43051144	77.20%	197.46	10043116	9223666	9525933	90711	8355	418117	0.41%

Sample ID	Number of input reads	Uniquely mapped reads number	Uniquely mapped reads %	Average mapped length	Number of splices: Total	Number of splices: Annotated (sjdb)	Number of splices: GT/AG	Number of splices: GC/AG	Number of splices: AT/AC	Number of splices: Non-canonical	Mismatch rate per base, %
1899623A	50819770	43112490	84.83%	198.17	18548503	17767697	18131357	126589	14039	276518	0.39%
1899739A	50078973	41704063	83.28%	197.83	16697470	15878434	16297908	109678	12002	277882	0.41%
1899845A	50337321	43418571	86.26%	197.86	14613965	13886385	14257986	96768	9876	249335	0.47%
1900924A	50348402	40942158	81.32%	197.82	17401917	16594734	16944663	119241	13236	324777	0.37%
1901282A	50240223	42044256	83.69%	197.51	16655319	15845062	16222097	115291	12215	305716	0.39%
1901448A	51184397	44273374	86.50%	198	16806211	16055401	16448605	106664	12080	238862	0.48%
1902019A	55525964	45721417	82.34%	196.81	15150230	14115294	14554139	118692	9986	467413	0.46%
1902831A	50800584	43439606	85.51%	197.96	17349684	16576320	16955042	114155	12065	268422	0.42%
1902979A	50418666	44238224	87.74%	198.2	17057939	16281713	16672681	110335	11224	263699	0.38%
1904061A	51902462	41604243	80.16%	197.71	15517399	14691799	15170422	98348	10825	237804	0.44%
1904585A	55243140	48041133	86.96%	198.07	18861392	17960443	18378237	131017	13691	338447	0.39%
1904783A	55634764	44425886	79.85%	196.59	13948267	12960164	13432249	102356	9355	404307	0.80%
1904904A	55506645	47691180	85.92%	197.94	12416565	11607909	11949485	94600	9032	363448	0.49%
1904949A	55805696	47643031	85.37%	198.03	18881887	18049807	18388207	132411	13842	347427	0.47%
1904971A	55447965	47882979	86.36%	198.02	17450049	16652877	17061500	115758	12677	260114	0.56%
1905067A	51012737	44273206	86.79%	198.01	18481353	17623422	18020362	126333	13098	321560	0.38%
1905138A	55715652	47049577	84.45%	197.73	18137830	17275114	17748578	111855	13407	263990	0.45%
1905350A	50407669	43453867	86.20%	197.86	17309063	16472057	16873725	116931	11779	306628	0.45%
1905402A	50970704	44338031	86.99%	197.92	17757140	16857818	17306040	121543	12293	317264	0.37%
1905470A	50327406	38422919	76.35%	198.15	18776174	18020559	18464656	122748	13216	175554	0.42%
1905577A	55653039	47910825	86.09%	198.28	23668162	22736007	23129224	172092	17627	349219	0.37%
1905833A	55390710	47359247	85.50%	198.22	13239896	12549450	12912100	90948	9143	227705	0.54%
1905977A	56096473	46233147	82.42%	197.78	20648370	19790874	20161610	150845	14715	321200	0.38%
1906132A	55616449	48300379	86.85%	197.94	16972317	16180523	16588171	110347	13935	259864	0.56%
1906391A	56143434	45477963	81.00%	196.9	16254447	14988401	15559481	127426	11681	555859	0.42%
1906588A	55196757	47235919	85.58%	198.12	17405741	16593766	16974466	121834	13155	296286	0.40%

Sample ID	Number of input reads	Uniquely mapped reads number	Uniquely mapped reads %	Average mapped length	Number of splices: Total	Number of splices: Annotated (sjdb)	Number of splices: GT/AG	Number of splices: GC/AG	Number of splices: AT/AC	Number of splices: Non-canonical	Mismatch rate per base, %
1906614A	55171880	47495287	86.09%	198.32	15813437	14975129	15450812	103915	11630	247080	0.42%
1907444A	55572884	46216207	83.16%	196.8	17509900	16394082	16900150	129631	11848	468271	0.45%
1908348A	56119352	46420678	82.72%	196.65	15802910	14668058	15158641	125869	13140	505260	0.48%
1908351A	55865260	46936643	84.02%	198.01	19111493	18284198	18694590	134229	13692	268982	0.44%
1908612A	56115115	41716493	74.34%	198.26	11106231	10553304	10862908	73208	7633	162482	0.56%
1908698A	55662373	44228613	79.46%	197.15	15661526	14622527	15015664	121382	12006	512474	0.54%
1908768A	55499626	46611950	83.99%	198.24	13154049	12464701	12849819	89209	9636	205385	0.38%
1909623A	55969434	46091280	82.35%	196.72	18647042	17353624	17896577	142159	13896	594410	0.46%
1910156A	56018168	45339356	80.94%	196.99	15990378	15020818	15543719	121582	12687	312390	0.54%
1910771A	55249750	38906253	70.42%	195.98	16493102	15441884	15770402	132120	10962	579618	0.43%
1911215A	55665559	45131028	81.08%	197.4	16622984	15777778	16156084	121298	12484	333118	0.46%
1911217A	55406093	48313152	87.20%	197.43	17528901	16491266	16936830	124559	13169	454343	0.47%
1911263A	55255920	45345158	82.06%	196.68	15682043	14541563	15034245	126032	12354	509412	0.48%
1911352A	48973135	40424081	82.54%	197.73	18033783	17215230	17668580	106349	12426	246428	0.41%
1911496A	45660318	36880471	80.77%	196.37	15170374	14198216	14672460	115168	11250	371496	0.47%
1911731A	55448035	47423405	85.53%	198.08	6861062	5970757	6209249	82816	8253	560744	0.43%
1912491A	42061569	31918838	75.89%	197.63	13915101	13232324	13629610	85500	9477	190514	0.39%
1913516A	55392392	48195101	87.01%	197.2	20315703	19256122	19814091	137568	14789	349255	0.39%
1913593A	55336353	47877805	86.52%	197.5	18408306	17348376	17941383	125951	14984	325988	0.40%
1914065A	55619835	43832198	78.81%	196.6	15339276	14265208	14708982	107376	12256	510662	0.47%
1915169A	55484734	46546700	83.89%	197.4	18626568	17680585	18189111	119811	12305	305341	0.45%
1915523A	55304621	47398681	85.70%	197.44	20916238	20003550	20406805	136346	16937	356150	0.39%
1915766A	55384626	46966127	84.80%	197.66	23087307	22039057	22620710	161253	18405	286939	0.43%
1916287A	55584704	49415097	88.90%	197.84	17205573	16169198	16679529	118461	12254	395329	0.43%
1917945A	55680731	48770151	87.59%	198.11	18551941	17658223	18116320	130554	12682	292385	0.39%
1918957A	55528300	47817696	86.11%	197.28	16970209	15809706	16411057	120141	13757	425254	0.41%

Sample ID	Number of input reads	Uniquely mapped reads number	Uniquely mapped reads %	Average mapped length	Number of splices: Total	Number of splices: Annotated (sjdb)	Number of splices: GT/AG	Number of splices: GC/AG	Number of splices: AT/AC	Number of splices: Non-canonical	Mismatch rate per base, %
1920204A	55606462	50104215	90.11%	198.04	19788216	18670193	19308040	131954	15276	332946	0.45%
1921957A	55850183	46576418	83.40%	196.42	21060786	20034750	20528103	145618	14807	372258	0.48%
1923328A	55518693	48952400	88.17%	197.72	12622064	11720142	12165452	92390	9791	354431	0.45%
1923420A	56141456	45488330	81.02%	196.91	17063593	16068303	16621627	110588	10903	320475	0.46%
1923483A	76839768	54021277	70.30%	196.56	22072318	20709105	21417297	154648	15893	484480	0.44%
1923555A	55562498	49989561	89.97%	198.04	14833702	13833839	14388693	107036	8157	329816	0.45%
1923712A	55653384	46708907	83.93%	196.36	17248604	16128461	16640973	130496	15037	462098	0.45%
1923760A	55448480	47119333	84.98%	196.38	22205263	21017860	21525906	161965	17447	499945	0.42%
1924049A	55264187	47904922	86.68%	196.83	20495277	19389278	19902705	138385	12998	441189	0.40%
1924478A	55357596	48970446	88.46%	197.62	17603954	16637418	17105344	118614	12000	367996	0.45%
1925252A	81943977	68742996	83.89%	197.03	22227785	20446050	21189352	188744	17763	831926	0.41%
1925846A	55116616	48869209	88.67%	197.2	17337239	16264055	16820836	122201	12791	381411	0.41%
1926132A	55399207	50758932	91.62%	198.18	13098576	12161167	12692798	91854	7469	306455	0.46%
1926272A	55495860	48551341	87.49%	197.47	19229193	18140844	18662862	138253	11986	416092	0.41%
1926306A	55300428	44342379	80.18%	197.59	14724422	13781531	14329349	92238	11096	291739	0.46%
1927301A	55150941	49959134	90.59%	198.16	14710049	13796744	14256905	102165	9915	341064	0.48%
1928219A	55226603	47621437	86.23%	197.65	18519199	17476606	17974541	125840	14078	404740	0.44%
1928232A	55715950	47568629	85.38%	197.42	14678877	13635804	14157256	101741	11378	408502	0.47%
1928274A	55540746	48243010	86.86%	197.68	15123450	14029007	14543457	118180	9897	451916	0.56%
1928455A	55095767	49757362	90.31%	197.93	17088308	15986244	16564399	123844	11323	388742	0.41%
1929671A	55266940	48282508	87.36%	197.89	18534792	17487648	18079270	117756	12007	325759	0.42%
1929976A	55656458	47546238	85.43%	197.47	15057168	14058065	14526753	115096	10118	405201	0.48%
1930827A	55552413	45214141	81.39%	197.22	19216183	18084465	18771640	122613	11403	310527	0.48%
1932301A	55275134	48307536	87.39%	197.34	15026038	13978807	14480487	114876	11739	418936	0.55%
1940746A	86325058	67219722	77.87%	196.64	34763496	32926450	33639344	218393	22068	883691	0.43%
1941919	84378028	68290039	80.93%	197.67	30699909	29230102	29807643	211888	20046	660332	0.39%

Sample ID	Number of input reads	Uniquely mapped reads number	Uniquely mapped reads %	Average mapped length	Number of splices: Total	Number of splices: Annotated (sjdb)	Number of splices: GT/AG	Number of splices: GC/AG	Number of splices: AT/AC	Number of splices: Non-canonical	Mismatch rate per base, %
1947162	89430673	69615164	77.84%	197.9	32344363	30969731	31569430	219636	19290	536007	0.40%
20180613-1A	170675534	144453199	84.64%	197.73	47641938	44182946	45850686	392065	36160	1363027	0.40%
20180613-3A	192124594	157719319	82.09%	197.54	63915639	59689678	61572173	508836	53188	1781442	0.36%
20180614-7A	170325859	145761592	85.58%	197.48	54563268	50553956	52580700	432151	45708	1504709	0.42%
20180614-9A	157219508	133131267	84.68%	197.46	48101086	44539593	46411008	390104	35890	1264084	0.40%
20180619-12A	155280577	136406718	87.85%	198.08	43543458	40935125	42646567	286455	27715	582721	0.46%
20180619-8A	158182821	136029897	86.00%	198.31	44038085	41734211	43148990	293022	31693	564380	0.38%
20180621-11A	164260672	134906405	82.13%	197.74	55368614	51913688	53803547	402166	38539	1124362	0.37%
20180621-16A	158314588	131369279	82.98%	197.65	49337904	46130390	47840484	382070	35956	1079394	0.38%
20180621-17A	164603400	140741653	85.50%	198.02	52630119	49550920	51143405	374055	38418	1074241	0.38%
20180621-18A	155133236	132624287	85.49%	197.98	49365113	46640113	48077514	338882	36065	912652	0.39%
20180621-1A	188248720	156871234	83.33%	198.01	58906866	55819105	57238337	405892	38730	1223907	0.39%
20180621-20A	164265574	135067273	82.22%	198.16	37719351	35187502	36766674	263527	25649	663501	0.43%
20180621-3A	157769628	136609256	86.59%	198.35	50934070	48346105	49781548	326344	36234	789944	0.39%
20180621-4A	156000504	133304283	85.45%	198.21	43749107	41225438	42538464	309809	31517	869317	0.40%
20180621-7A	189299198	148995420	78.71%	197.6	63593190	59720006	61533612	477748	47517	1534313	0.40%
20180621-8A	157931370	128470932	81.35%	198.11	51095291	48354291	49787516	348246	38273	921256	0.36%
20180621-9A	167509335	138593019	82.74%	197.98	50281409	47489514	49181191	342059	38009	720150	0.38%
3-19-1435611A	106188595	83441643	78.58%	196.87	28824639	26286738	26990490	272448	26465	1535236	0.43%
3-31-1930444A	143482917	115856310	80.75%	196.65	43101270	40113057	41726350	289257	28048	1057615	0.46%
4-10-1932654A	82770773	64372581	77.77%	196.71	29467877	27733875	28497017	207601	22198	741061	0.41%
4-2-1922690A	87704380	70948282	80.89%	196.78	22160603	20328017	21075144	193828	18224	873407	0.44%
4-3-1927708A	80979216	62811913	77.57%	196.6	24686746	23023580	23651236	187780	17229	830501	0.47%

Sample ID	Number of input reads	Uniquely mapped reads number	Uniquely mapped reads %	Average mapped length	Number of splices: Total	Number of splices: Annotated (sjdb)	Number of splices: GT/AG	Number of splices: GC/AG	Number of splices: AT/AC	Number of splices: Non-canonical	Mismatch rate per base, %
4-8-1932227A	83150592	68770352	82.71%	196.93	28876899	27085037	27854273	207508	20582	794536	0.43%
8125630A	50368727	43752776	86.86%	197.93	16427945	15588725	16068826	99399	10553	249167	0.45%
8128463A	46937495	40956645	87.26%	197.76	16015288	15273614	15660511	114437	10565	229775	0.49%
8128721A	32984599	19094178	57.89%	197.19	2109346	1493009	1637830	19165	2212	450139	0.78%
8129174A	55612502	45566026	81.93%	197.78	18634875	17714481	18123754	129895	13153	368073	0.39%
8131850A	55484167	43308905	78.06%	196.81	17021060	16084917	16544874	121208	12877	342101	0.52%
8132228A	50429382	43132547	85.53%	198.04	20532547	19701099	20069569	138704	15290	308984	0.43%
8133283A	55305477	45148649	81.64%	197.91	18834538	17982384	18449303	104192	10874	270169	0.49%
8133808A	38449120	32058052	83.38%	198.28	4373536	3980922	4108071	36936	3971	224558	0.52%
8133904A	50777272	44456420	87.55%	197.98	12713438	11928674	12274912	95897	9811	332818	0.45%
8134049A	50277297	44197712	87.91%	197.9	19037613	18205811	18700905	118794	14337	203577	0.38%
8134785C	50116488	42267394	84.34%	197.64	15453390	14659152	15002825	103570	10597	336398	0.67%
8135483A	50734742	43007233	84.77%	198.12	23457079	22669169	23058584	150226	17654	230615	0.42%
8136591A	45359179	38274323	84.38%	196.85	12780478	11876388	12181271	106265	10780	482162	0.49%

Table S3. Genes for classification of CRC patients from controls.

Ensembl ID	Enterz ID	Gene Symbol
ENSG00000169598	1677	DFFB
ENSG00000171603	22883	CLSTN1
ENSG00000171824	5394	EXOSC10
ENSG00000162490	374946	DRAXIN
ENSG00000236936	NA	NA
ENSG00000162551	249	ALPL
ENSG00000175130	65108	MARCKSL1
ENSG00000142694	55194	EVA1B
ENSG00000196449	79693	YRDC
ENSG00000084072	10450	PPIE
ENSG00000066185	84217	ZMYND12
ENSG00000117448	10327	AKR1A1
ENSG00000142973	1580	CYP4B1
ENSG00000162377	65260	COA7
ENSG00000079739	5236	PGM1
ENSG00000162437	55225	RAVER2
ENSG00000184588	5142	PDE4B
ENSG00000213516	494115	RBMXL1
ENSG00000154451	115362	GBP5
ENSG00000122484	79871	RPAP2
ENSG00000079335	8556	CDC14A
ENSG00000117543	51611	DPH5
ENSG00000177301	3737	KCNA2
ENSG00000156171	128338	DRAM2
ENSG00000198799	9860	LRIG2
ENSG00000173218	81839	VANGL1
ENSG00000134250	4853	NOTCH2
ENSG00000230850	NA	NA
ENSG00000215861	653513	LOC653513
ENSG00000143450	51686	OAZ3
ENSG00000163220	6280	S100A9
ENSG00000163221	6283	S100A12
ENSG00000158764	142683	ITLN2
ENSG00000143226	2212	FCGR2A
ENSG00000135845	5279	PIGC
ENSG00000234741	60674	GAS5
ENSG00000075391	9462	RASAL2
ENSG00000116668	54823	SWT1
ENSG00000081237	5788	PTPRC
ENSG00000143486	1939	EIF2D
ENSG00000143512	79802	HHIPL2

Ensembl ID	Enterz ID	Gene Symbol
ENSG00000187554	7100	TLR5
ENSG00000116574	58480	RHOU
ENSG00000010072	83932	SPRTN
ENSG00000059588	6894	TARBP1
ENSG00000151694	6868	ADAM17
ENSG00000084693	60509	AGBL5
ENSG00000213626	81606	LBH
ENSG00000115816	10153	CEBPZ
ENSG00000236213	NA	NA
ENSG00000119729	23433	RHOQ
ENSG00000232046	100507073	LINC01798
ENSG00000124374	400961	PAIP2B
ENSG00000132300	55037	PTCD3
ENSG00000115042	51011	FAHD2A
ENSG00000135945	51455	REV1
ENSG00000115641	2274	FHL2
ENSG00000175701	205251	SMIM37
ENSG00000125538	3553	IL1B
ENSG00000136709	55339	WDR33
ENSG00000232606	NA	NA
ENSG00000162980	26225	ARL5A
ENSG00000115165	9595	CYTIP
ENSG00000182263	55137	FIGN
ENSG00000138385	6741	SSB
ENSG00000224063	NA	NA
ENSG00000128699	94101	ORMDL1
ENSG00000138378	6775	STAT4
ENSG00000081320	9262	STK17B
ENSG00000240344	53938	PPIL3
ENSG00000204217	659	BMPR2
ENSG00000023228	4719	NDUFS1
ENSG00000115414	2335	FN1
ENSG00000153823	55022	PID1
ENSG00000066248	25791	NGEF
ENSG00000131375	23473	CAPN7
ENSG00000179152	285343	TCAIM
ENSG00000136068	2317	FLNB
ENSG00000144749	26018	LRIG1
ENSG00000163380	56203	LMOD3
ENSG00000113966	84100	ARL6
ENSG00000057019	131566	DCBLD2
ENSG00000154174	9868	TOMM70

Ensembl ID	Enterz ID	Gene Symbol
ENSG00000144820	84873	ADGRG7
ENSG00000154175	25890	ABI3BP
ENSG00000151576	79691	QTRT2
ENSG00000144843	141	ADPRH
ENSG00000163870	131601	TPRA1
ENSG00000248932	100507291	LOC100507291
ENSG00000198829	56670	SUCNR1
ENSG00000114416	8087	FXR1
ENSG00000244405	2119	ETV5
ENSG00000168824	27065	NSG1
ENSG00000152990	166647	ADGRA3
ENSG00000038210	55300	PI4K2B
ENSG00000109814	7358	UGDH
ENSG00000249348	100885776	UGDH-AS1
ENSG00000173597	27284	SULT1B1
ENSG00000163104	56916	SMARCAD1
ENSG00000164038	133308	SLC9B2
ENSG00000164022	9255	AIMP1
ENSG00000172399	51778	MYOZ2
ENSG00000245958	645513	LOC645513
ENSG00000164168	55751	TMEM184C
ENSG00000129116	23022	PALLD
ENSG00000109586	51809	GALNT7
ENSG00000213430	NA	NA
ENSG00000113460	55299	BRIX1
ENSG00000067113	8611	PLPP1
ENSG00000164512	79722	ANKRD55
ENSG00000086189	27292	DIMT1
ENSG00000113593	23398	PPWD1
ENSG00000113595	373	TRIM23
ENSG00000122012	22987	SV2C
ENSG00000152413	9456	HOMER1
ENSG00000247828	100505894	TMEM161B-AS1
ENSG00000081189	4208	MEF2C
ENSG00000164307	51752	ERAP1
ENSG00000174136	285704	RGMB
ENSG00000129595	64097	EPB41L4A
ENSG00000145779	25816	TNFAIP8
ENSG00000244921	NA	NA
ENSG00000120314	54853	WDR55
ENSG00000070814	6949	TCOF1

Ensembl ID	Enterz ID	Gene Symbol
ENSG00000131459	9945	GFPT2
ENSG00000145982	10667	FARS2
ENSG00000238221	NA	NA
ENSG00000111843	51522	TMEM14C
ENSG00000112186	10486	CAP2
ENSG00000112304	55856	ACOT13
ENSG00000146109	29777	ABT1
ENSG00000096654	7738	ZNF184
ENSG00000204475	259197	NCR3
ENSG00000250535	NA	NA
ENSG00000204287	3122	HLA-DRA
ENSG00000170734	5429	POLH
ENSG00000180992	64928	MRPL14
ENSG00000112742	7272	TTK
ENSG00000013375	5238	PGM3
ENSG00000111880	8732	RNGTT
ENSG00000132423	51805	COQ3
ENSG00000112297	202	CRYBG1
ENSG00000196591	3066	HDAC2
ENSG00000188820	441168	CALHM6
ENSG00000234117	NA	NA
ENSG00000047932	57120	GOPC
ENSG00000196569	3908	LAMA2
ENSG00000079819	2037	EPB41L2
ENSG00000118503	7128	TNFAIP3
ENSG00000120254	25902	MTHFD1L
ENSG00000092820	7430	EZR
ENSG00000197081	3482	IGF2R
ENSG00000184465	253769	WDR27
ENSG00000164916	221937	FOXK1
ENSG00000136261	28969	BZW2
ENSG00000136237	9771	RAPGEF5
ENSG00000153814	221895	JAZF1
ENSG00000105778	23080	AVL9
ENSG00000211699	NA	NA
ENSG00000214765	641977	SEPT7P2
ENSG00000198039	10793	ZNF273
ENSG00000223473	NA	NA
ENSG00000106638	26608	TBL2
ENSG00000214293	100505854	APTR
ENSG00000205413	54809	SAMD9
ENSG00000066923	10734	STAG3

Ensembl ID	Enterz ID	Gene Symbol
ENSG00000230257	58160	NFE4
ENSG00000184408	3751	KCND2
ENSG00000128513	25913	POT1
ENSG00000158623	26958	COPG2
ENSG00000244479	NA	NA
ENSG00000106526	653857	ACTR3C
ENSG00000213203	170575	GIMAP1
ENSG00000241735	220832	FABP5P3
ENSG00000124343	7499	XG
ENSG00000102048	140462	ASB9
ENSG00000102010	660	BMX
ENSG00000165168	1536	CYBB
ENSG00000102103	10084	PQBP1
ENSG00000204466	NA	NA
ENSG00000158578	212	ALAS2
ENSG00000082458	1741	DLG3
ENSG00000147164	29934	SNX12
ENSG00000165259	139324	HDX
ENSG00000179083	286499	FAM133A
ENSG00000158427	286527	TMSB15B
ENSG00000232160	101928578	RAP2C-AS1
ENSG00000182319	NA	NA
ENSG00000175445	4023	LPL
ENSG00000158863	64760	FAM160B2
ENSG00000147535	84513	PLPP5
ENSG00000047249	51606	ATP6V1H
ENSG00000147570	85479	DNAJC5B
ENSG00000164751	5828	PEX2
ENSG00000254266	101927003	PKIA-AS1
ENSG00000237264	NA	NA
ENSG00000155100	51633	OTUD6B
ENSG00000120963	51123	ZNF706
ENSG00000246263	101927221	UBR5-AS1
ENSG00000164929	79870	BAALC
ENSG00000164935	81501	DCSTAMP
ENSG00000172167	27085	MTBP
ENSG00000170881	11236	RNF139
ENSG00000042832	7038	TG
ENSG00000155926	6503	SLA
ENSG00000178685	84875	PARP10
ENSG00000137103	51754	TMEM8B
ENSG00000135048	23670	CEMIP2

Ensembl ID	Enterz ID	Gene Symbol
ENSG00000148019	84131	CEP78
ENSG00000119522	57706	DENND1A
ENSG00000136878	10868	USP20
ENSG00000148296	6838	SURF6
ENSG00000172889	51162	EGFL7
ENSG00000197070	92714	ARRDC1
ENSG00000134461	54522	ANKRD16
ENSG00000134470	3601	IL15RA
ENSG00000152457	64421	DCLRE1C
ENSG00000165983	9317	PTER
ENSG00000180592	387640	SKIDA1
ENSG00000175395	219749	ZNF25
ENSG00000151151	253430	IPMK
ENSG00000221817	101929145	PPP3CB-AS1
ENSG00000172586	118487	CHCHD1
ENSG00000156110	132	ADK
ENSG00000148655	83938	LRMDA
ENSG00000173124	142827	ACSM6
ENSG00000138185	953	ENTPD1
ENSG00000226688	728558	ENTPD1-AS1
ENSG00000177853	9849	ZNF518A
ENSG00000077150	4791	NFKB2
ENSG00000119927	57678	GPAM
ENSG00000198924	9937	DCLRE1A
ENSG00000165669	63877	FAM204A
ENSG00000183605	119559	SFXN4
ENSG00000165672	10935	PRDX3
ENSG00000179988	118672	PSTK
ENSG00000142089	10410	IFITM3
ENSG00000269821	10984	KCNQ1OT1
ENSG00000196565	3048	HBG2
ENSG00000171714	203859	ANO5
ENSG00000205213	55366	LGR4
ENSG00000176697	627	BDNF
ENSG00000109911	26610	ELP4
ENSG00000186714	493860	CCDC73
ENSG00000254879	NA	NA
ENSG00000255189	NA	NA
ENSG00000197629	219972	MPEG1
ENSG00000162227	10629	TAF6L
ENSG00000173457	26472	PPP1R14B
ENSG00000014138	23649	POLA2

Ensembl ID	Enterz ID	Gene Symbol
ENSG00000173442	254102	EHBP1L1
ENSG00000173039	5970	RELA
ENSG00000172922	84153	RNASEH2C
ENSG00000087365	10992	SF3B2
ENSG00000175634	6199	RPS6KB2
ENSG00000184154	220074	LRTOMT
ENSG00000241170	NA	NA
ENSG00000254429	NA	NA
ENSG00000149257	871	SERPINH1
ENSG00000137507	2615	LRRC32
ENSG00000227376	NA	NA
ENSG00000165490	220042	DDIAS
ENSG00000042429	9440	MED17
ENSG00000137693	10413	YAP1
ENSG00000150768	1737	DLAT
ENSG00000150776	55216	NKAPD1
ENSG00000166741	4837	NNMT
ENSG00000186174	283149	BCL9L
ENSG00000186166	338657	CCDC84
ENSG00000064309	50937	CDON
ENSG00000002016	5893	RAD52
ENSG00000255775	NA	NA
ENSG00000010278	928	CD9
ENSG00000250510	27239	GPR162
ENSG00000260423	101930452	LINC02367
ENSG00000139112	23710	GABARAPL1
ENSG00000084444	57613	FAM234B
ENSG00000151490	5800	PTPRO
ENSG00000111713	2998	GYS2
ENSG00000256377	NA	NA
ENSG00000139567	94	ACVRL1
ENSG00000111077	23371	TNS2
ENSG00000181852	10193	RNF41
ENSG00000123329	64333	ARHGAP9
ENSG00000177990	283417	DPY19L2
ENSG00000090382	4069	LYZ
ENSG00000180881	84698	CAPS2
ENSG00000136040	10154	PLXNC1
ENSG00000111144	4048	LTA4H
ENSG00000135127	92558	BICDL1
ENSG00000257218	283459	GATC
ENSG00000135124	5025	P2RX4

Ensembl ID	Enterz ID	Gene Symbol
ENSG00000170633	80196	RNF34
ENSG00000033030	55596	ZCCHC8
ENSG00000234152	NA	NA
ENSG00000139517	222484	LNX2
ENSG00000102738	10240	MRPS31
ENSG00000023516	11215	AKAP11
ENSG00000152213	115761	ARL11
ENSG00000231607	8847	DLEU2
ENSG00000188243	170622	COMMD6
ENSG00000139746	64062	RBM26
ENSG00000228889	100289373	UBAC2-AS1
ENSG00000175198	5095	PCCA
ENSG00000100814	57820	CCNB1IP1
ENSG00000165819	56339	METTL3
ENSG00000100813	22985	ACIN1
ENSG00000215277	100507650	RNF212B
ENSG00000215271	57594	HOMEZ
ENSG00000186648	90668	CARMIL3
ENSG00000100911	5721	PSME2
ENSG00000092098	55072	RNF31
ENSG00000100916	84312	BRMS1L
ENSG00000198513	51062	ATL1
ENSG00000198554	11169	WDHD1
ENSG00000131981	3958	LGALS3
ENSG00000139946	57161	PELI2
ENSG00000126785	57381	RHOJ
ENSG00000119596	56252	YLPM1
ENSG00000197249	5265	SERPINA1
ENSG00000198690	22909	FAN1
ENSG00000103994	64397	ZNF106
ENSG00000104177	50804	MYEF2
ENSG00000166477	123169	LEO1
ENSG00000103599	64799	IQCH
ENSG00000178802	4351	MPI
ENSG00000178761	57184	FAM219B
ENSG00000198794	192683	SCAMP5
ENSG00000140398	79661	NEIL1
ENSG00000167196	26263	FBXO22
ENSG00000140391	10099	TSPAN3
ENSG00000180953	400410	ST20
ENSG00000188659	283726	SAXO2
ENSG00000131876	6627	SNRPA1

Ensembl ID	Enterz ID	Gene Symbol
ENSG00000238098	650655	ABCA17P
ENSG00000131652	79228	THOC6
ENSG00000162086	7627	ZNF75A
ENSG00000089486	29965	CDIP1
ENSG00000179889	23042	PDXDC1
ENSG00000058600	55718	POLR3E
ENSG00000188603	1201	CLN3
ENSG00000261239	NA	NA
ENSG00000171241	79801	SHCBP1
ENSG00000121281	113	ADCY7
ENSG00000125148	4502	MT2A
ENSG00000135736	92922	CCDC102A
ENSG00000185669	333929	SNAI3
ENSG00000185324	8558	CDK10
ENSG00000187741	2175	FANCA
ENSG00000167720	63826	SRR
ENSG00000141258	9905	SGSM2
ENSG00000040531	1497	CTNS
ENSG00000182557	201305	SPNS3
ENSG00000167842	79003	MIS12
ENSG00000011295	54902	TTC19
ENSG00000226478	NA	NA
ENSG00000109084	27346	TMEM97
ENSG00000108733	5193	PEX12
ENSG00000172660	NA	NA
ENSG00000224298	NA	NA
ENSG00000141140	NA	NA
ENSG00000108753	NA	NA
ENSG00000125691	9349	RPL23
ENSG00000173786	1267	CNP
ENSG00000184922	752	FMNL1
ENSG00000167107	80221	ACSF2
ENSG00000006282	64847	SPATA20
ENSG00000121057	8165	AKAP1
ENSG00000008283	1534	CYB561
ENSG00000186665	284018	C17orf58
ENSG00000266714	80022	MYO15B
ENSG00000214140	768206	PRCD
ENSG00000163597	100507246	SNHG16
ENSG00000262873	NA	NA
ENSG00000176155	284001	CCDC57
ENSG00000176890	7298	TYMS

Ensembl ID	Enterz ID	Gene Symbol
ENSG00000132199	55556	ENOSF1
ENSG00000264235	104968399	LOC104968399
ENSG00000154655	91133	L3MBTL4
ENSG00000264876	NA	NA
ENSG00000154856	147495	APCDD1
ENSG00000177150	125228	FAM210A
ENSG00000141446	114799	ESCO1
ENSG00000101773	5932	RBBP8
ENSG00000134508	91768	CABLES1
ENSG00000134042	83876	MRO
ENSG00000166634	89777	SERPINB12
ENSG00000213742	102724826	ZNF337-AS1
ENSG00000125967	63941	NECAB3
ENSG00000080845	22839	DLGAP4
ENSG00000196562	55959	SULF2
ENSG00000124243	55653	BCAS4
ENSG00000124224	NA	NA
ENSG00000171469	93134	ZNF561
ENSG00000223547	284391	ZNF844
ENSG00000268278	NA	NA
ENSG00000213971	NA	NA
ENSG00000198453	374900	ZNF568
ENSG00000086544	80271	ITPKC
ENSG00000105755	23474	ETHE1
ENSG00000169169	126129	CPT1C
ENSG00000104960	53635	PTOV1
ENSG00000161551	84765	ZNF577
ENSG00000170954	55786	ZNF415
ENSG00000167615	114823	LENG8
ENSG00000104972	10859	LILRB1
ENSG00000133247	84787	KMT5C
ENSG00000178935	79818	ZNF552
ENSG00000099725	5616	PRKY
ENSG00000184979	11274	USP18
ENSG00000099977	1652	DDT
ENSG00000178026	388886	LRRC75B
ENSG00000225465	NA	NA
ENSG00000183530	253143	PRR14L
ENSG00000133466	114904	C1QTNF6
ENSG00000100079	3957	LGALS2
ENSG00000184381	8398	PLA2G6
ENSG00000243811	140564	APOBEC3D

Ensembl ID	Enterz ID	Gene Symbol
ENSG00000186976	64800	EFCAB6
ENSG00000138964	64098	PARVG
ENSG00000160191	5152	PDE9A
ENSG00000183250	84536	LINC01547
ENSG00000248527	NA	NA
ENSG00000179403	64856	VWA1
ENSG00000116251	6146	RPL22
ENSG00000171735	23261	CAMTA1
ENSG00000116288	11315	PARK7
ENSG00000142634	79180	EFHD2
ENSG00000132881	79363	CPLANE2
ENSG00000127481	23352	UBR4
ENSG00000090686	84196	USP48
ENSG00000142798	3339	HSPG2
ENSG00000204219	6920	TCEA3
ENSG00000117318	3399	ID3
ENSG00000188672	6006	RHCE
ENSG00000131914	79727	LIN28A
ENSG00000204160	84243	ZDHHC18
ENSG00000130768	27293	SMPDL3B
ENSG00000182866	3932	LCK
ENSG00000065978	4904	YBX1
ENSG00000117399	991	CDC20
ENSG00000117410	533	ATP6V0B
ENSG00000117472	10103	TSPAN1
ENSG00000173660	7388	UQCRH
ENSG00000232022	NA	NA
ENSG00000117834	200010	SLC5A9
ENSG00000203356	105378716	LINC01562
ENSG00000177606	3725	JUN
ENSG00000237928	100996570	NFIA-AS2
ENSG00000132849	10207	PATJ
ENSG00000240563	54596	L1TD1
ENSG00000237726	NA	NA
ENSG00000162620	127255	LRRIQ3
ENSG00000142875	5567	PRKACB
ENSG00000162643	126820	WDR63
ENSG00000162695	148867	SLC30A7
ENSG00000116266	6814	STXBP3
ENSG00000143126	1952	CELSR2
ENSG00000143093	85369	STRIP1
ENSG00000198765	6847	SYCP1

Ensembl ID	Enterz ID	Gene Symbol
ENSG00000163399	476	ATP1A1
ENSG00000183508	54855	FAM46C
ENSG00000134193	83998	REG4
ENSG00000143537	8751	ADAM15
ENSG00000143248	8490	RGS5
ENSG00000224286	284688	LINC01142
ENSG00000197959	26052	DNM3
ENSG00000162779	126859	AXDND1
ENSG00000162782	163589	TDRD5
ENSG00000143337	26092	TOR1AIP1
ENSG00000162704	10092	ARPC5
ENSG00000000971	3075	CFH
ENSG00000214796	NA	NA
ENSG00000133048	1116	CHI3L1
ENSG00000133065	254428	SLC41A1
ENSG00000143502	55061	SUSD4
ENSG00000162923	80232	WDR26
ENSG00000135773	10753	CAPN9
ENSG00000116991	57568	SIPA1L2
ENSG00000119285	55127	HEATR1
ENSG00000162843	128025	WDR64
ENSG00000162852	163882	CNST
ENSG00000171863	6201	RPS7
ENSG00000235092	100506299	ID2-AS1
ENSG00000143797	129642	MBOAT2
ENSG00000224400	NA	NA
ENSG00000119801	51646	YPEL5
ENSG00000169564	5093	PCBP1
ENSG00000183733	344018	FIGLA
ENSG00000243264	NA	NA
ENSG00000241244	NA	NA
ENSG00000211633	NA	NA
ENSG00000168754	51252	FAM178B
ENSG00000168658	200403	VWA3B
ENSG00000072163	55679	LIMS2
ENSG00000168702	53353	LRP1B
ENSG00000150556	130576	LYPD6B
ENSG00000115183	85461	TANC1
ENSG00000144285	6323	SCN1A
ENSG00000138382	29081	METTL5
ENSG00000071967	79901	CYBRD1
ENSG00000138448	3685	ITGAV

**Ensembl ID**	**Enterz ID**	**Gene Symbol**
ENSG00000168542	1281	COL3A1
ENSG00000128641	4430	MYO1B
ENSG00000237525	NA	NA
ENSG00000124006	23363	OBSL1
ENSG00000116106	2043	EPHA4
ENSG00000115009	6364	CCL20
ENSG00000124831	9208	LRRFIP1
ENSG00000065802	51665	ASB1
ENSG00000196277	2917	GRM7
ENSG00000144713	6161	RPL32
ENSG00000170876	79188	TMEM43
ENSG00000174748	6138	RPL15
ENSG00000091317	54918	CMTM6
ENSG00000168028	3921	RPSA
ENSG00000188846	9045	RPL14
ENSG00000172037	3913	LAMB2
ENSG00000224479	NA	NA
ENSG00000183662	407738	FAM19A1
ENSG00000144746	10550	ARL6IP5
ENSG00000244674	NA	NA
ENSG00000114455	11148	HHLA2
ENSG00000144821	22989	MYH15
ENSG00000240893	101929694	LINC02042
ENSG00000031081	57514	ARHGAP31
ENSG00000173706	57493	HEG1
ENSG00000181804	285195	SLC9A9
ENSG00000120742	27230	SERP1
ENSG00000181631	53829	P2RY13
ENSG00000152580	285313	IGSF10
ENSG00000244268	NA	NA
ENSG00000237787	NA	NA
ENSG00000114204	5276	SERPINI2
ENSG00000240292	NA	NA
ENSG00000213123	255758	TCTEX1D2
ENSG00000182903	170960	ZNF721
ENSG00000163945	57654	UVSSA
ENSG00000071127	9948	WDR1
ENSG00000163257	54876	DCAF16
ENSG00000109805	64151	NCAPG
ENSG00000250038	NA	NA
ENSG00000078140	3093	UBE2K
ENSG00000064042	22998	LIMCH1

Ensembl ID	Enterz ID	Gene Symbol
ENSG00000250657	NA	NA
ENSG00000163285	2565	GABRG1
ENSG00000145321	2638	GC
ENSG00000124875	6372	CXCL6
ENSG00000260265	NA	NA
ENSG00000156194	5470	PPEF2
ENSG00000138758	55752	11-Sep
ENSG00000145332	57563	KLHL8
ENSG00000187653	NA	NA
ENSG00000153064	55024	BANK1
ENSG00000248373	NA	NA
ENSG00000205403	3426	CFI
ENSG00000164096	401152	C4orf3
ENSG00000196159	79633	FAT4
ENSG00000153132	1047	CLGN
ENSG00000171557	2266	FGG
ENSG00000151790	6999	TDO2
ENSG00000145476	285440	CYP4V2
ENSG00000145494	4726	NDUFS6
ENSG00000133398	84246	MED10
ENSG00000248294	NA	NA
ENSG00000226259	NA	NA
ENSG00000157111	134285	TMEM171
ENSG00000164347	84340	GFM2
ENSG00000189127	340120	ANKRD34B
ENSG00000172497	134526	ACOT12
ENSG00000152348	83734	ATG10
ENSG00000145715	5921	RASA1
ENSG00000145777	85480	TSLP
ENSG00000152503	55521	TRIM36
ENSG00000168916	57507	ZNF608
ENSG00000238160	NA	NA
ENSG00000249119	NA	NA
ENSG00000248923	NA	NA
ENSG00000120727	51247	PAIP2
ENSG00000113140	6678	SPARC
ENSG00000155511	2890	GRIA1
ENSG00000022355	2554	GABRA1
ENSG00000113240	57396	CLK4
ENSG00000176783	80230	RUFY1
ENSG00000153046	9425	CDYL
ENSG00000124491	2162	F13A1

Ensembl ID	Enterz ID	Gene Symbol
ENSG00000096696	1832	DSP
ENSG00000230939	NA	NA
ENSG00000146038	51473	DCDC2
ENSG00000180596	8347	HIST1H2BC
ENSG00000180573	8334	HIST1H2AC
ENSG00000158373	3017	HIST1H2BD
ENSG00000168274	NA	NA
ENSG00000124557	696	BTN1A1
ENSG00000225173	NA	NA
ENSG00000213722	23564	DDAH2
ENSG00000204386	4758	NEU1
ENSG00000231500	6222	RPS18
ENSG00000204220	10471	PFDN6
ENSG00000096433	3710	ITPR3
ENSG00000112164	2740	GLP1R
ENSG00000188056	285852	TREML4
ENSG00000124733	4201	MEA1
ENSG00000096006	10321	CRISP3
ENSG00000079841	22999	RIMS1
ENSG00000186231	114792	KLHL32
ENSG00000155130	NA	NA
ENSG00000203760	387103	CENPW
ENSG00000028839	9519	TBPL1
ENSG00000225177	NA	NA
ENSG00000131016	9590	AKAP12
ENSG00000130338	56995	TULP4
ENSG00000112096	6648	SOD2
ENSG00000272549	401286	LINC02538
ENSG00000223855	441307	HRAT92
ENSG00000236039	101927630	LOC101927630
ENSG00000271133	101927811	LOC101927811
ENSG00000136231	10643	IGF2BP3
ENSG00000105926	51678	MPP6
ENSG00000180354	222166	MTURN
ENSG00000122643	51251	NT5C3A
ENSG00000106624	165	AEBP1
ENSG00000106078	23242	COBL
ENSG00000129103	25870	SUMF2
ENSG00000006704	9569	GTF2IRD1
ENSG00000188175	253012	HEPACAM2
ENSG00000160862	563	AZGP1
ENSG00000197093	79690	GAL3ST4

**Ensembl ID**	**Enterz ID**	**Gene Symbol**
ENSG00000091136	3912	LAMB1
ENSG00000128585	4289	MKLN1
ENSG00000240268	100289017	MOXD2P
ENSG00000127377	155051	CRYGN
ENSG00000105983	64327	LMBR1
ENSG00000205542	7114	TMSB4X
ENSG00000147003	57393	CLTRN
ENSG00000177324	139105	BEND2
ENSG00000184368	256714	MAP7D2
ENSG00000047597	7504	XK
ENSG00000147113	79742	CXorf36
ENSG00000102265	7076	TIMP1
ENSG00000234068	203569	PAGE2
ENSG00000102053	340554	ZC3H12B
ENSG00000230629	NA	NA
ENSG00000225470	554203	JPX
ENSG00000131171	6451	SH3BGRL
ENSG00000184867	9823	ARMCX2
ENSG00000198932	9737	GPRASP1
ENSG00000133131	79710	MORC4
ENSG00000101842	340547	VSIG1
ENSG00000131724	3597	IL13RA1
ENSG00000175556	79836	LONRF3
ENSG00000125356	4694	NDUFA1
ENSG00000125676	57187	THOC2
ENSG00000134590	8933	RTL8C
ENSG00000147378	89885	FATE1
ENSG00000130830	4354	MPP1
ENSG00000180190	157695	TDRP
ENSG00000164821	1669	DEFA4
ENSG00000254235	157273	LOC157273
ENSG00000164733	1508	CTSB
ENSG00000253490	101929450	LINC02099
ENSG00000133872	51669	SARAF
ENSG00000129691	9070	ASH2L
ENSG00000255101	NA	NA
ENSG00000147485	137902	PXDNL
ENSG00000254006	NA	NA
ENSG00000245910	641638	SNHG6
ENSG00000040341	27067	STAU2
ENSG00000104435	11075	STMN2
ENSG00000251136	101929709	LOC101929709

Ensembl ID	Enterz ID	Gene Symbol
ENSG00000248858	441369	FLJ46284
ENSG00000253197	NA	NA
ENSG00000254224	NA	NA
ENSG00000132561	4147	MATN2
ENSG00000164924	7534	YWHAZ
ENSG00000172164	6641	SNTB1
ENSG00000255080	NA	NA
ENSG00000173334	10221	TRIB1
ENSG00000153310	51571	FAM49B
ENSG00000008513	6482	ST3GAL1
ENSG00000107020	55848	PLGRKT
ENSG00000107165	7306	TYRP1
ENSG00000107186	8777	MPDZ
ENSG00000044459	54875	CNTLN
ENSG00000230453	441459	ANKRD18B
ENSG00000172159	257019	FRMD3
ENSG00000254473	105376114	LOC105376114
ENSG00000197506	64078	SLC28A3
ENSG00000131669	4814	NINJ1
ENSG00000136938	10541	ANP32B
ENSG00000106771	23731	TMEM245
ENSG00000119411	54836	BSPRY
ENSG00000041982	3371	TNC
ENSG00000148175	2040	STOM
ENSG00000148358	57720	GPR107
ENSG00000050555	10319	LAMC3
ENSG00000235106	266655	BRD3OS
ENSG00000122140	51116	MRPS2
ENSG00000184925	286256	LCN12
ENSG00000235281	NA	NA
ENSG00000077327	9576	SPAG6
ENSG00000229932	NA	NA
ENSG00000151023	219670	ENKUR
ENSG00000095777	53904	MYO3A
ENSG00000151033	119180	LYZL2
ENSG00000170759	3799	KIF5B
ENSG00000150093	3688	ITGB1
ENSG00000165512	7570	ZNF22
ENSG00000165633	196740	VSTM4
ENSG00000108176	56521	DNAJC12
ENSG00000095585	29760	BLNK
ENSG00000203867	282996	RBM20

Ensembl ID	Enterz ID	Gene Symbol
ENSG00000108061	8036	SHOC2
ENSG00000148908	6001	RGS10
ENSG00000176769	256536	TCERG1L
ENSG00000177106	64787	EPS8L2
ENSG00000110169	3263	HPX
ENSG00000166340	1200	TPP1
ENSG00000166796	3948	LDHC
ENSG00000165970	9152	SLC6A5
ENSG00000121621	81930	KIF18A
ENSG00000085063	966	CD59
ENSG00000149115	85456	TNKS1BP1
ENSG00000134827	6947	TCN1
ENSG00000149534	2206	MS4A2
ENSG00000110079	51338	MS4A4A
ENSG00000124942	79026	AHNAK
ENSG00000175550	10589	DRAP1
ENSG00000174080	8722	CTSF
ENSG00000255980	NA	NA
ENSG00000131626	8500	PPFIA1
ENSG00000110237	9828	ARHGEF17
ENSG00000175582	5870	RAB6A
ENSG00000137491	11309	SLCO2B1
ENSG00000215504	NA	NA
ENSG00000149196	51501	HIKESHI
ENSG00000204403	100506742	CASP12
ENSG00000204397	114769	CARD16
ENSG00000149289	85463	ZC3H12C
ENSG00000166736	3359	HTR3A
ENSG00000110324	3587	IL10RA
ENSG00000149428	10525	HYOU1
ENSG00000254612	NA	NA
ENSG00000177406	100049716	LOC100049716
ENSG00000118971	894	CCND2
ENSG00000187569	359787	DPPA3
ENSG00000214851	253128	LINC00612
ENSG00000247157	338817	LINC01252
ENSG00000171681	55729	ATF7IP
ENSG00000111341	4256	MGP
ENSG00000123095	79365	BHLHE41
ENSG00000165935	341346	SMCO2
ENSG00000110841	8496	PPFIBP1
ENSG00000257176	100506606	LOC100506606

**Ensembl ID**	**Enterz ID**	**Gene Symbol**
ENSG00000245614	100506660	DDX11-AS1
ENSG00000134283	51535	PPHLN1
ENSG00000139168	85437	ZCRB1
ENSG00000184613	4753	NELL2
ENSG00000257924	NA	NA
ENSG00000079337	10411	RAPGEF3
ENSG00000123352	65244	SPATS2
ENSG00000050438	9498	SLC4A8
ENSG00000167780	8435	SOAT2
ENSG00000172819	5916	RARG
ENSG00000065361	2065	ERBB3
ENSG00000196531	4666	NACA
ENSG00000213352	NA	NA
ENSG00000187109	4673	NAP1L1
ENSG00000072041	55117	SLC6A15
ENSG00000257345	NA	NA
ENSG00000139344	144193	AMDHD1
ENSG00000012504	9971	NR1H4
ENSG00000111199	59341	TRPV4
ENSG00000111412	79794	C12orf49
ENSG00000111445	5985	RFC5
ENSG00000089220	5037	PEBP1
ENSG00000150991	7316	UBC
ENSG00000132964	1024	CDK8
ENSG00000189403	3146	HMGB1
ENSG00000133083	9201	DCLK1
ENSG00000136167	3936	LCP1
ENSG00000231473	100862704	RB1-DT
ENSG00000136153	4008	LMO7
ENSG00000088387	23348	DOCK9
ENSG00000187498	1282	COL4A1
ENSG00000088448	55608	ANKRD10
ENSG00000129566	7011	TEP1
ENSG00000092067	1053	CEBPE
ENSG00000129473	599	BCL2L2
ENSG00000166091	116173	CMTM5
ENSG00000129515	58533	SNX6
ENSG00000151748	60485	SAV1
ENSG00000089775	7597	ZBTB25
ENSG00000119688	5826	ABCD4
ENSG00000119684	27030	MLH3
ENSG00000089916	55668	GPATCH2L

Ensembl ID	Enterz ID	Gene Symbol
ENSG00000258819	102724190	LINC02289
ENSG00000165409	7253	TSHR
ENSG00000197119	123096	SLC25A29
ENSG00000211896	NA	NA
ENSG00000211897	NA	NA
ENSG00000225698	NA	NA
ENSG00000215252	440270	GOLGA8B
ENSG00000104081	90427	BMF
ENSG00000166887	23339	VPS39
ENSG00000140263	6652	SORD
ENSG00000137857	53905	DUOX1
ENSG00000128849	84952	CGNL1
ENSG00000166104	NA	NA
ENSG00000140416	7168	TPM1
ENSG00000035664	23604	DAPK2
ENSG00000260776	NA	NA
ENSG00000167202	23102	TBC1D2B
ENSG00000185787	10933	MORF4L1
ENSG00000058335	5923	RASGRF1
ENSG00000259445	NA	NA
ENSG00000172183	3669	ISG20
ENSG00000140526	11057	ABHD2
ENSG00000185033	10509	SEMA4B
ENSG00000259582	101927310	LOC101927310
ENSG00000006327	51330	TNFRSF12A
ENSG00000140623	124404	12-Sep
ENSG00000262097	101927311	LINC02185
ENSG00000087250	4504	MT3
ENSG00000179776	1003	CDH5
ENSG00000159708	55282	LRRC36
ENSG00000182810	55794	DDX28
ENSG00000214353	100130894	VAC14-AS1
ENSG00000103035	5713	PSMD7
ENSG00000166816	197257	LDHD
ENSG00000103196	83716	CRISPLD2
ENSG00000268804	NA	NA
ENSG00000141503	50488	MINK1
ENSG00000181885	1366	CLDN7
ENSG00000132518	3000	GUCY2D
ENSG00000072210	224	ALDH3A2
ENSG00000226521	NA	NA
ENSG00000182271	388364	TMIGD1

Ensembl ID	Enterz ID	Gene Symbol
ENSG00000129277	NA	NA
ENSG00000267671	NA	NA
ENSG00000141753	3487	IGFBP4
ENSG00000167925	84514	GHDC
ENSG00000131469	6155	RPL27
ENSG00000161654	124801	LSM12
ENSG00000168591	79089	TMUB2
ENSG00000267278	100133991	MAP3K14-AS1
ENSG00000186868	4137	MAPT
ENSG00000178852	124989	EFCAB13
ENSG00000005884	3675	ITGA3
ENSG00000108821	1277	COL1A1
ENSG00000153930	162282	ANKFN1
ENSG00000062716	81671	VMP1
ENSG00000108506	57508	INTS2
ENSG00000170412	55890	GPRC5C
ENSG00000109062	9368	SLC9A3R1
ENSG00000267123	101928710	LINC02081
ENSG00000108679	3959	LGALS3BP
ENSG00000101745	23253	ANKRD12
ENSG00000101782	8780	RIOK3
ENSG00000046604	1829	DSG2
ENSG00000267390	NA	NA
ENSG00000221887	284293	HMSD
ENSG00000122490	80148	PQLC1
ENSG00000244588	642636	RAD21L1
ENSG00000125844	6238	RRBP1
ENSG00000125810	22918	CD93
ENSG00000125968	3397	ID1
ENSG00000101346	23509	POFUT1
ENSG00000101421	128866	CHMP4B
ENSG00000125971	83658	DYNLRB1
ENSG00000196756	388796	SNHG17
ENSG00000198900	7150	TOP1
ENSG00000124145	6385	SDC4
ENSG00000124164	9217	VAPB
ENSG00000197561	1991	ELANE
ENSG00000130270	148229	ATP8B3
ENSG00000105278	23217	ZFR2
ENSG00000167769	125981	ACER1
ENSG00000099783	4670	HNRNPM
ENSG00000142347	4542	MYO1F

Ensembl ID	Enterz ID	Gene Symbol
ENSG00000130816	1786	DNMT1
ENSG00000129353	57153	SLC44A2
ENSG00000198429	7620	ZNF69
ENSG00000123143	5585	PKN1
ENSG00000167460	7171	TPM4
ENSG00000130520	25804	LSM4
ENSG00000105707	3249	HPN
ENSG00000233214	NA	NA
ENSG00000090920	NA	NA
ENSG00000105372	6223	RPS19
ENSG00000118156	84215	ZNF541
ENSG00000063177	6141	RPL18
ENSG00000171049	2358	FPR2
ENSG00000189068	284415	VSTM1
ENSG00000223638	342931	RFPL4A
ENSG00000160505	147945	NLRP4
ENSG00000268205	NA	NA
ENSG00000129824	6192	RPS4Y1
ENSG00000154620	9087	TMSB4Y
ENSG00000215580	286554	BCORP1
ENSG00000099889	421	ARVCF
ENSG00000099901	5902	RANBP1
ENSG00000223553	NA	NA
ENSG00000187905	400891	LRRC74B
ENSG00000100218	27156	RSPH14
ENSG00000100228	9609	RAB36
ENSG00000099998	2687	GGT5
ENSG00000100219	7494	XBP1
ENSG00000226471	NA	NA
ENSG00000133488	284904	SEC14L4
ENSG00000128245	7533	YWHAH
ENSG00000128311	7263	TST
ENSG00000100092	23616	SH3BP1
ENSG00000100206	11144	DMC1
ENSG00000100311	5155	PDGFB
ENSG00000100316	6122	RPL3
ENSG00000100416	55687	TRMU
ENSG00000142192	351	APP
ENSG00000185917	54093	SETD4
ENSG00000177398	89766	UMODL1
ENSG00000142173	1292	COL6A2

Table S4. Genes for classification of CRC patients, healthy donors and patients with noncancerous diseases.

Ensembl ID	Entrez ID	Gene Symbol
ENSG00000127054	54973	INTS11
ENSG00000171603	22883	CLSTN1
ENSG00000160049	1676	DFFA
ENSG00000162490	374946	DRAXIN
ENSG00000236936	NA	NA
ENSG00000130695	64793	CEP85
ENSG00000000938	2268	FGR
ENSG00000160050	79140	CCDC28B
ENSG00000220785	NA	NA
ENSG00000142694	55194	EVA1B
ENSG00000084072	10450	PPIE
ENSG00000066185	84217	ZMYND12
ENSG00000159479	112950	MED8
ENSG00000117448	10327	AKR1A1
ENSG00000162377	65260	COA7
ENSG00000142856	23421	ITGB3BP
ENSG00000116652	NA	NA
ENSG00000162437	55225	RAVER2
ENSG00000162613	8880	FUBP1
ENSG00000154451	115362	GBP5
ENSG00000235777	100873933	DPYD-AS2
ENSG00000137996	8634	RTCA
ENSG00000177301	3737	KCNA2
ENSG00000156171	128338	DRAM2
ENSG00000198799	9860	LRIG2
ENSG00000052723	80143	SIKE1
ENSG00000173218	81839	VANGL1
ENSG00000225241	NA	NA
ENSG00000215861	653513	LOC653513
ENSG00000143369	1893	ECM1
ENSG00000163131	1520	CTSS
ENSG00000143434	10500	SEMA6C
ENSG00000163220	6280	S100A9
ENSG00000143554	11000	SLC27A3
ENSG00000162739	114836	SLAMF6
ENSG00000188404	6402	SELL
ENSG00000135845	5279	PIGC
ENSG00000234741	60674	GAS5
ENSG00000116161	27101	CACYBP
ENSG00000075391	9462	RASAL2

Ensembl ID	Entrez ID	Gene Symbol
ENSG00000116668	54823	SWT1
ENSG00000023572	51022	GLRX2
ENSG00000081237	5788	PTPRC
ENSG00000143486	1939	EIF2D
ENSG00000228106	NA	NA
ENSG00000187554	7100	TLR5
ENSG00000059588	6894	TARBP1
ENSG00000151694	6868	ADAM17
ENSG00000205795	192668	CYS1
ENSG00000169016	1876	E2F6
ENSG00000079785	1653	DDX1
ENSG00000119771	114818	KLHL29
ENSG00000138092	79172	CENPO
ENSG00000115207	2976	GTF3C2
ENSG00000198522	11321	GPN1
ENSG00000243147	9553	MRPL33
ENSG00000213626	81606	LBH
ENSG00000115816	10153	CEBPZ
ENSG00000115944	9167	COX7A2L
ENSG00000119729	23433	RHOQ
ENSG00000028116	7444	VRK2
ENSG00000162928	5194	PEX13
ENSG00000232046	100507073	LINC01798
ENSG00000124374	400961	PAIP2B
ENSG00000042493	822	CAPG
ENSG00000132300	55037	PTCD3
ENSG00000135945	51455	REV1
ENSG00000175701	205251	SMIM37
ENSG00000153208	10461	MERTK
ENSG00000125611	84269	CHCHD5
ENSG00000136709	55339	WDR33
ENSG00000232606	NA	NA
ENSG00000136536	64844	MARCH7
ENSG00000182263	55137	FIGN
ENSG00000169507	151258	SLC38A11
ENSG00000138385	6741	SSB
ENSG00000162998	2487	FRZB
ENSG00000224063	NA	NA
ENSG00000138378	6775	STAT4
ENSG00000081320	9262	STK17B
ENSG00000240344	53938	PPIL3
ENSG00000163596	130026	ICA1L

Ensembl ID	Entrez ID	Gene Symbol
ENSG00000023228	4719	NDUFS1
ENSG00000223725	NA	NA
ENSG00000030419	22807	IKZF2
ENSG00000156973	5147	PDE6D
ENSG00000066248	25791	NGEF
ENSG00000132321	79781	IQCA1
ENSG00000153558	25827	FBXL2
ENSG00000008226	9940	DLEC1
ENSG00000179152	285343	TCAIM
ENSG00000068745	51447	IP6K2
ENSG00000185909	200942	KLHDC8B
ENSG00000164068	63891	RNF123
ENSG00000088543	51161	C3orf18
ENSG00000023330	211	ALAS1
ENSG00000163380	56203	LMOD3
ENSG00000113966	84100	ARL6
ENSG00000144802	64332	NFKBIZ
ENSG00000181722	26137	ZBTB20
ENSG00000144843	141	ADPRH
ENSG00000163755	84343	HPS3
ENSG00000198829	56670	SUCNR1
ENSG00000114790	26084	ARHGEF26
ENSG00000079257	56925	LXN
ENSG00000171757	151827	LRRC34
ENSG00000144962	83893	SPATA16
ENSG00000090530	55214	P3H2
ENSG00000188958	257313	UTS2B
ENSG00000187527	344905	ATP13A5
ENSG00000198836	4976	OPA1
ENSG00000168824	27065	NSG1
ENSG00000196526	60312	AFAP1
ENSG00000109814	7358	UGDH
ENSG00000109189	64854	USP46
ENSG00000249242	441027	TMEM150C
ENSG00000172399	51778	MYOZ2
ENSG00000245958	645513	LOC645513
ENSG00000164168	55751	TMEM184C
ENSG00000109686	152503	SH3D19
ENSG00000109654	23321	TRIM2
ENSG00000109586	51809	GALNT7
ENSG00000249915	10016	PDCD6
ENSG00000213430	NA	NA

Ensembl ID	Entrez ID	Gene Symbol
ENSG00000113460	55299	BRIX1
ENSG00000198865	100129792	CCDC152
ENSG00000164172	4338	MOCS2
ENSG00000067113	8611	PLPP1
ENSG00000164512	79722	ANKRD55
ENSG00000113593	23398	PPWD1
ENSG00000152413	9456	HOMER1
ENSG00000081189	4208	MEF2C
ENSG00000153113	831	CAST
ENSG00000129595	64097	EPB41L4A
ENSG00000145779	25816	TNFAIP8
ENSG00000164244	133619	PRRC1
ENSG00000244921	NA	NA
ENSG00000146013	2676	GFRA3
ENSG00000120738	1958	EGR1
ENSG00000070814	6949	TCOF1
ENSG00000039600	11063	SOX30
ENSG00000155858	134353	LSM11
ENSG00000174705	285590	SH3PXD2B
ENSG00000164466	94081	SFXN1
ENSG00000131459	9945	GFPT2
ENSG00000111843	51522	TMEM14C
ENSG00000112186	10486	CAP2
ENSG00000112304	55856	ACOT13
ENSG00000010704	3077	HFE
ENSG00000158406	8365	HIST1H4H
ENSG00000146109	29777	ABT1
ENSG00000096654	7738	ZNF184
ENSG00000137404	11270	NRM
ENSG00000204475	259197	NCR3
ENSG00000204287	3122	HLA-DRA
ENSG00000198502	3127	HLA-DRB5
ENSG00000112640	5528	PPP2R5D
ENSG00000180992	64928	MRPL14
ENSG00000112077	6005	RHAG
ENSG00000188107	346007	EYS
ENSG00000118407	27145	FILIP1
ENSG00000013375	5238	PGM3
ENSG00000111886	2570	GABRR2
ENSG00000123552	85015	USP45
ENSG00000112297	202	CRYBG1
ENSG00000243587	NA	NA

Ensembl ID	Entrez ID	Gene Symbol
ENSG00000196591	3066	HDAC2
ENSG00000188820	441168	CALHM6
ENSG00000047932	57120	GOPC
ENSG00000196569	3908	LAMA2
ENSG00000118515	6446	SGK1
ENSG00000118503	7128	TNFAIP3
ENSG00000092820	7430	EZR
ENSG00000106399	6119	RPA3
ENSG00000136261	28969	BZW2
ENSG00000106546	196	AHR
ENSG00000136237	9771	RAPGEF5
ENSG00000156928	115416	MALSU1
ENSG00000153814	221895	JAZF1
ENSG00000106086	84725	PLEKHA8
ENSG00000105778	23080	AVL9
ENSG00000211698	NA	NA
ENSG00000211699	NA	NA
ENSG00000198039	10793	ZNF273
ENSG00000214293	100505854	APTR
ENSG00000127928	2792	GNGT1
ENSG00000196652	23660	ZKSCAN5
ENSG00000164675	154865	IQUB
ENSG00000128513	25913	POT1
ENSG00000237243	NA	NA
ENSG00000122778	57670	KIAA1549
ENSG00000257743	93432	MGAM2
ENSG00000213203	170575	GIMAP1
ENSG00000002933	55365	TMEM176A
ENSG00000178234	63917	GALNT11
ENSG00000241735	220832	FABP5P3
ENSG00000214106	100132707	PAXIP1-AS2
ENSG00000157212	22976	PAXIP1
ENSG00000102010	660	BMX
ENSG00000086712	55787	TXLNG
ENSG00000184831	79135	APOO
ENSG00000158427	286527	TMSB15B
ENSG00000182319	NA	NA
ENSG00000175445	4023	LPL
ENSG00000241852	541565	C8orf58
ENSG00000147535	84513	PLPP5
ENSG00000047249	51606	ATP6V1H
ENSG00000008988	6224	RPS20

Ensembl ID	Entrez ID	Gene Symbol
ENSG00000164751	5828	PEX2
ENSG00000237264	NA	NA
ENSG00000155100	51633	OTUD6B
ENSG00000188343	137392	FAM92A
ENSG00000120963	51123	ZNF706
ENSG00000246263	101927221	UBR5-AS1
ENSG00000164929	79870	BAALC
ENSG00000164935	81501	DCSTAMP
ENSG00000172167	27085	MTBP
ENSG00000042832	7038	TG
ENSG00000155926	6503	SLA
ENSG00000178685	84875	PARP10
ENSG00000147852	7436	VLDLR
ENSG00000187742	79048	SECISBP2
ENSG00000196312	84278	MFSD14C
ENSG00000136936	7507	XPA
ENSG00000056558	7185	TRAF1
ENSG00000136877	2356	FPGS
ENSG00000223551	NA	NA
ENSG00000130707	445	ASS1
ENSG00000148296	6838	SURF6
ENSG00000123453	1757	SARDH
ENSG00000196139	8644	AKR1C3
ENSG00000134470	3601	IL15RA
ENSG00000151657	22944	KIN
ENSG00000229124	100507347	VIM-AS1
ENSG00000151151	253430	IPMK
ENSG00000198954	26128	KIF1BP
ENSG00000107742	9806	SPOCK2
ENSG00000138303	51008	ASCC1
ENSG00000166348	159195	USP54
ENSG00000214655	23053	ZSWIM8
ENSG00000148655	83938	LRMDA
ENSG00000152782	53354	PANK1
ENSG00000107443	54619	CCNJ
ENSG00000227492	NA	NA
ENSG00000099194	6319	SCD
ENSG00000077150	4791	NFKB2
ENSG00000179988	118672	PSTK
ENSG00000196565	3048	HBG2
ENSG00000171714	203859	ANO5
ENSG00000176697	627	BDNF

Ensembl ID	Entrez ID	Gene Symbol
ENSG00000109911	26610	ELP4
ENSG00000254879	NA	NA
ENSG00000255189	NA	NA
ENSG00000166902	54948	MRPL16
ENSG00000176485	11145	PLA2G16
ENSG00000014138	23649	POLA2
ENSG00000173039	5970	RELA
ENSG00000172543	1521	CTSW
ENSG00000087365	10992	SF3B2
ENSG00000174165	254359	ZDHHC24
ENSG00000175634	6199	RPS6KB2
ENSG00000184154	220074	LRTOMT
ENSG00000166435	143570	XRRA1
ENSG00000254429	NA	NA
ENSG00000149257	871	SERPINH1
ENSG00000171533	4135	MAP6
ENSG00000137507	2615	LRRC32
ENSG00000227376	NA	NA
ENSG00000087884	28971	AAMDC
ENSG00000042429	9440	MED17
ENSG00000137693	10413	YAP1
ENSG00000150776	55216	NKAPD1
ENSG00000166741	4837	NNMT
ENSG00000002016	5893	RAD52
ENSG00000171860	719	C3AR1
ENSG00000111796	3820	KLRB1
ENSG00000139112	23710	GABARAPL1
ENSG00000151490	5800	PTPRO
ENSG00000121350	79912	PYROXD1
ENSG00000256377	NA	NA
ENSG00000175548	144245	ALG10B
ENSG00000111077	23371	TNS2
ENSG00000181852	10193	RNF41
ENSG00000175203	10540	DCTN2
ENSG00000135407	10677	AVIL
ENSG00000177990	283417	DPY19L2
ENSG00000090382	4069	LYZ
ENSG00000173401	256710	GLIPR1L1
ENSG00000139278	11010	GLIPR1
ENSG00000111615	11103	KRR1
ENSG00000111144	4048	LTA4H
ENSG00000120805	400	ARL1

Ensembl ID	Entrez ID	Gene Symbol
ENSG00000255857	100506649	PXN-AS1
ENSG00000257218	283459	GATC
ENSG00000135124	5025	P2RX4
ENSG00000110931	10645	CAMKK2
ENSG00000170633	80196	RNF34
ENSG00000033030	55596	ZCCHC8
ENSG00000130921	91574	C12orf65
ENSG00000139517	222484	LNX2
ENSG00000102738	10240	MRPS31
ENSG00000231607	8847	DLEU2
ENSG00000136122	79866	BORA
ENSG00000175198	5095	PCCA
ENSG00000215271	57594	HOMEZ
ENSG00000186648	90668	CARMIL3
ENSG00000139908	283629	TSSK4
ENSG00000100916	84312	BRMS1L
ENSG00000139946	57161	PELI2
ENSG00000126814	57570	TRMT5
ENSG00000126785	57381	RHOJ
ENSG00000119596	56252	YLPM1
ENSG00000197249	5265	SERPINA1
ENSG00000185215	7127	TNFAIP2
ENSG00000166166	115708	TRMT61A
ENSG00000227729	647286	RD3L
ENSG00000226777	9834	FAM30A
ENSG00000224078	NA	NA
ENSG00000103599	64799	IQCH
ENSG00000178802	4351	MPI
ENSG00000198794	192683	SCAMP5
ENSG00000140398	79661	NEIL1
ENSG00000169410	5780	PTPN9
ENSG00000167196	26263	FBXO22
ENSG00000188659	283726	SAXO2
ENSG00000131876	6627	SNRPA1
ENSG00000238098	650655	ABCA17P
ENSG00000162065	57465	TBC1D24
ENSG00000131652	79228	THOC6
ENSG00000167984	197358	NLRC3
ENSG00000179889	23042	PDXDC1
ENSG00000134419	6210	RPS15A
ENSG00000271609	NA	NA
ENSG00000058600	55718	POLR3E

Ensembl ID	Entrez ID	Gene Symbol
ENSG00000140750	55114	ARHGAP17
ENSG00000188603	1201	CLN3
ENSG00000261239	NA	NA
ENSG00000171241	79801	SHCBP1
ENSG00000187193	4501	MT1X
ENSG00000104731	54758	KLHDC4
ENSG00000185324	8558	CDK10
ENSG00000070366	23293	SMG6
ENSG00000167720	63826	SRR
ENSG00000141258	9905	SGSM2
ENSG00000040531	1497	CTNS
ENSG00000167842	79003	MIS12
ENSG00000215067	100506713	ALOX12-AS1
ENSG00000221926	10626	TRIM16
ENSG00000154025	125206	SLC5A10
ENSG00000154898	NA	NA
ENSG00000214832	NA	NA
ENSG00000109084	27346	TMEM97
ENSG00000007202	9703	KIAA0100
ENSG00000092871	117584	RFFL
ENSG00000172660	NA	NA
ENSG00000141140	NA	NA
ENSG00000108753	NA	NA
ENSG00000056661	NA	NA
ENSG00000167107	80221	ACSF2
ENSG00000006282	64847	SPATA20
ENSG00000154975	56934	CA10
ENSG00000121053	8288	EPX
ENSG00000189050	51136	RNFT1
ENSG00000008283	1534	CYB561
ENSG00000271605	284021	MILR1
ENSG00000266714	80022	MYO15B
ENSG00000214140	768206	PRCD
ENSG00000163597	100507246	SNHG16
ENSG00000267506	NA	NA
ENSG00000262873	NA	NA
ENSG00000176155	284001	CCDC57
ENSG00000079134	9984	THOC1
ENSG00000176890	7298	TYMS
ENSG00000154655	91133	L3MBTL4
ENSG00000177150	125228	FAM210A
ENSG00000101773	5932	RBBP8

Ensembl ID	Entrez ID	Gene Symbol
ENSG00000153339	22878	TRAPPC8
ENSG00000134769	1837	DTNA
ENSG00000152217	26040	SETBP1
ENSG00000134042	83876	MRO
ENSG00000091157	23335	WDR7
ENSG00000242550	5273	SERPINB10
ENSG00000125901	64949	MRPS26
ENSG00000171984	149840	C20orf196
ENSG00000101230	140862	ISM1
ENSG00000101004	22981	NINL
ENSG00000213742	102724826	ZNF337-AS1
ENSG00000101412	1869	E2F1
ENSG00000080845	22839	DLGAP4
ENSG00000198026	63925	ZNF335
ENSG00000198185	55713	ZNF334
ENSG00000124243	55653	BCAS4
ENSG00000124224	NA	NA
ENSG00000171469	93134	ZNF561
ENSG00000090339	3383	ICAM1
ENSG00000223547	284391	ZNF844
ENSG00000197857	51710	ZNF44
ENSG00000105607	2639	GCDH
ENSG00000130309	79709	COLGALT1
ENSG00000105750	7639	ZNF85
ENSG00000268278	NA	NA
ENSG00000213971	NA	NA
ENSG00000153879	1054	CEBPG
ENSG00000153902	163175	LGI4
ENSG00000268947	NA	NA
ENSG00000011600	7305	TYROBP
ENSG00000086544	80271	ITPKC
ENSG00000167578	53916	RAB4B
ENSG00000079462	5050	PAFAH1B3
ENSG00000267680	7767	ZNF224
ENSG00000230510	100506012	PPP5D1
ENSG00000178150	163071	ZNF114
ENSG00000169169	126129	CPT1C
ENSG00000104960	53635	PTOV1
ENSG00000170954	55786	ZNF415
ENSG00000133247	84787	KMT5C
ENSG00000178935	79818	ZNF552
ENSG00000099725	5616	PRKY

Ensembl ID	Entrez ID	Gene Symbol
ENSG00000215193	55670	PEX26
ENSG00000100027	29799	YPEL1
ENSG00000272216	NA	NA
ENSG00000178026	388886	LRRC75B
ENSG00000100150	9681	DEPDC5
ENSG00000100234	7078	TIMP3
ENSG00000100079	3957	LGALS2
ENSG00000184381	8398	PLA2G6
ENSG00000100226	9567	GTPBP1
ENSG00000186976	64800	EFCAB6
ENSG00000156253	10069	RWDD2B
ENSG00000159147	29980	DONSON
ENSG00000160191	5152	PDE9A
ENSG00000160255	3689	ITGB2
ENSG00000225630	NA	NA
ENSG00000237973	NA	NA
ENSG00000248527	NA	NA
ENSG00000179403	64856	VWA1
ENSG00000116251	6146	RPL22
ENSG00000225077	NA	NA
ENSG00000175262	148345	C1orf127
ENSG00000127481	23352	UBR4
ENSG00000142798	3339	HSPG2
ENSG00000070831	998	CDC42
ENSG00000204219	6920	TCEA3
ENSG00000117318	3399	ID3
ENSG00000117305	3155	HMGCL
ENSG00000130768	27293	SMPDL3B
ENSG00000134697	29889	GNL2
ENSG00000169218	284654	RSPO1
ENSG00000131236	10487	CAP1
ENSG00000065978	4904	YBX1
ENSG00000117834	200010	SLC5A9
ENSG00000203356	105378716	LINC01562
ENSG00000162407	8613	PLPP3
ENSG00000134716	1573	CYP2J2
ENSG00000237928	100996570	NFIA-AS2
ENSG00000132849	10207	PATJ
ENSG00000203963	400757	C1orf141
ENSG00000142864	26135	SERBP1
ENSG00000162620	127255	LRRIQ3
ENSG00000137968	204962	SLC44A5

Ensembl ID	Entrez ID	Gene Symbol
ENSG00000142875	5567	PRKACB
ENSG00000162643	126820	WDR63
ENSG00000213512	388646	GBP7
ENSG00000237435	102723661	LOC102723661
ENSG00000188641	1806	DPYD
ENSG00000162695	148867	SLC30A7
ENSG00000143126	1952	CELSR2
ENSG00000143093	85369	STRIP1
ENSG00000116473	5906	RAP1A
ENSG00000198162	10905	MAN1A2
ENSG00000134193	83998	REG4
ENSG00000232265	100505824	LOC100505824
ENSG00000143546	6279	S100A8
ENSG00000143537	8751	ADAM15
ENSG00000233540	100874284	DNM3-IT1
ENSG00000162779	126859	AXDND1
ENSG00000162782	163589	TDRD5
ENSG00000143337	26092	TOR1AIP1
ENSG00000143333	6004	RGS16
ENSG00000058085	3918	LAMC2
ENSG00000162704	10092	ARPC5
ENSG00000116679	10625	IVNS1ABP
ENSG00000214796	NA	NA
ENSG00000133048	1116	CHI3L1
ENSG00000133065	254428	SLC41A1
ENSG00000143502	55061	SUSD4
ENSG00000162909	824	CAPN2
ENSG00000154358	84033	OBSCN
ENSG00000135773	10753	CAPN9
ENSG00000119285	55127	HEATR1
ENSG00000162843	128025	WDR64
ENSG00000162852	163882	CNST
ENSG00000115705	7173	TPO
ENSG00000186487	23040	MYT1L
ENSG00000214866	728597	DCDC2C
ENSG00000235576	NA	NA
ENSG00000134308	10971	YWHAQ
ENSG00000224400	NA	NA
ENSG00000213639	5500	PPP1CB
ENSG00000230876	285045	LINC00486
ENSG00000177994	129852	C2orf73
ENSG00000115380	2202	EFEMP1

Ensembl ID	Entrez ID	Gene Symbol
ENSG00000119862	29094	LGALSL
ENSG00000169564	5093	PCBP1
ENSG00000118640	8673	VAMP8
ENSG00000243264	NA	NA
ENSG00000241244	NA	NA
ENSG00000211633	NA	NA
ENSG00000168754	51252	FAM178B
ENSG00000168658	200403	VWA3B
ENSG00000169756	3987	LIMS1
ENSG00000115091	10096	ACTR3
ENSG00000115112	29842	TFCP2L1
ENSG00000072163	55679	LIMS2
ENSG00000238860	NA	NA
ENSG00000121989	92	ACVR2A
ENSG00000073737	10170	DHRS9
ENSG00000115252	5136	PDE1A
ENSG00000138448	3685	ITGAV
ENSG00000168542	1281	COL3A1
ENSG00000243478	NA	NA
ENSG00000204186	57683	ZDBF2
ENSG00000114948	8745	ADAM23
ENSG00000144406	285175	UNC80
ENSG00000135929	1593	CYP27A1
ENSG00000123989	79586	CHPF
ENSG00000116106	2043	EPHA4
ENSG00000178602	150677	OTOS
ENSG00000196277	2917	GRM7
ENSG00000170876	79188	TMEM43
ENSG00000092345	1618	DAZL
ENSG00000231304	100874028	SGO1-AS1
ENSG00000174748	6138	RPL15
ENSG00000091317	54918	CMTM6
ENSG00000188846	9045	RPL14
ENSG00000241186	6997	TDGF1
ENSG00000172037	3913	LAMB2
ENSG00000187672	26059	ERC2
ENSG00000144724	5793	PTPRG
ENSG00000163618	8618	CADPS
ENSG00000183662	407738	FAM19A1
ENSG00000144746	10550	ARL6IP5
ENSG00000081154	57092	PCNP
ENSG00000144821	22989	MYH15

Ensembl ID	Entrez ID	Gene Symbol
ENSG00000091986	151887	CCDC80
ENSG00000185565	4045	LSAMP
ENSG00000031081	57514	ARHGAP31
ENSG00000221955	84561	SLC12A8
ENSG00000196353	131034	CPNE4
ENSG00000240086	NA	NA
ENSG00000242104	NA	NA
ENSG00000169903	7104	TM4SF4
ENSG00000152580	285313	IGSF10
ENSG00000244268	NA	NA
ENSG00000197415	79674	VEPH1
ENSG00000182903	170960	ZNF721
ENSG00000163956	4043	LRPAP1
ENSG00000071127	9948	WDR1
ENSG00000109805	64151	NCAPG
ENSG00000249645	NA	NA
ENSG00000163285	2565	GABRG1
ENSG00000109171	57606	SLAIN2
ENSG00000128040	6691	SPINK2
ENSG00000169116	25849	PARM1
ENSG00000260265	NA	NA
ENSG00000156194	5470	PPEF2
ENSG00000138760	950	SCARB2
ENSG00000138758	55752	SEPT11
ENSG00000197826	255119	CFAP299
ENSG00000145287	51316	PLAC8
ENSG00000145332	57563	KLHL8
ENSG00000170509	345275	HSD17B13
ENSG00000248373	NA	NA
ENSG00000164109	4085	MAD2L1
ENSG00000249464	285419	LINC01091
ENSG00000077684	79960	JADE1
ENSG00000153132	1047	CLGN
ENSG00000109670	55294	FBXW7
ENSG00000151790	6999	TDO2
ENSG00000251511	NA	NA
ENSG00000109572	1182	CLCN3
ENSG00000250131	NA	NA
ENSG00000145476	285440	CYP4V2
ENSG00000223361	NA	NA
ENSG00000248294	NA	NA
ENSG00000226259	NA	NA

Ensembl ID	Entrez ID	Gene Symbol
ENSG00000172497	134526	ACOT12
ENSG00000152348	83734	ATG10
ENSG00000151422	2241	FER
ENSG00000145777	85480	TSLP
ENSG00000238160	NA	NA
ENSG00000249119	NA	NA
ENSG00000248923	NA	NA
ENSG00000152377	6695	SPOCK1
ENSG00000164266	6690	SPINK1
ENSG00000211445	2878	GPX3
ENSG00000113140	6678	SPARC
ENSG00000022355	2554	GABRA1
ENSG00000250274	100128059	LOC100128059
ENSG00000113240	57396	CLK4
ENSG00000176783	80230	RUFY1
ENSG00000244041	NA	NA
ENSG00000124491	2162	F13A1
ENSG00000223342	NA	NA
ENSG00000096696	1832	DSP
ENSG00000229931	NA	NA
ENSG00000137261	9856	KIAA0319
ENSG00000180596	8347	HIST1H2BC
ENSG00000197459	NA	NA
ENSG00000225173	NA	NA
ENSG00000204580	780	DDR1
ENSG00000213722	23564	DDAH2
ENSG00000204386	4758	NEU1
ENSG00000231500	6222	RPS18
ENSG00000204220	10471	PFDN6
ENSG00000112164	2740	GLP1R
ENSG00000096006	10321	CRISP3
ENSG00000186231	114792	KLHL32
ENSG00000173214	91749	MFSD4B
ENSG00000155130	NA	NA
ENSG00000203760	387103	CENPW
ENSG00000146376	93663	ARHGAP18
ENSG00000216917	NA	NA
ENSG00000118514	64577	ALDH8A1
ENSG00000225177	NA	NA
ENSG00000120265	5110	PCMT1
ENSG00000198729	81706	PPP1R14C
ENSG00000131016	9590	AKAP12

Ensembl ID	Entrez ID	Gene Symbol
ENSG00000130338	56995	TULP4
ENSG00000112096	6648	SOD2
ENSG00000086232	27102	EIF2AK1
ENSG00000236039	101927630	LOC101927630
ENSG00000271133	101927811	LOC101927811
ENSG00000105926	51678	MPP6
ENSG00000122643	51251	NT5C3A
ENSG00000122545	989	SEPT7
ENSG00000106624	165	AEBP1
ENSG00000106078	23242	COBL
ENSG00000146648	1956	EGFR
ENSG00000006704	9569	GTF2IRD1
ENSG00000164692	1278	COL1A2
ENSG00000160868	1576	CYP3A4
ENSG00000091136	3912	LAMB1
ENSG00000128609	4698	NDUFA5
ENSG00000128585	4289	MKLN1
ENSG00000198074	57016	AKR1B10
ENSG00000105929	50617	ATP6V0A4
ENSG00000240268	100289017	MOXD2P
ENSG00000211745	NA	NA
ENSG00000205542	7114	TMSB4X
ENSG00000147003	57393	CLTRN
ENSG00000177324	139105	BEND2
ENSG00000047597	7504	XK
ENSG00000189221	4128	MAOA
ENSG00000147113	79742	CXorf36
ENSG00000102265	7076	TIMP1
ENSG00000102053	340554	ZC3H12B
ENSG00000230629	NA	NA
ENSG00000229807	7503	XIST
ENSG00000225470	554203	JPX
ENSG00000131171	6451	SH3BGRL
ENSG00000198932	9737	GPRASP1
ENSG00000260831	NA	NA
ENSG00000133131	79710	MORC4
ENSG00000101842	340547	VSIG1
ENSG00000131724	3597	IL13RA1
ENSG00000125676	57187	THOC2
ENSG00000134590	8933	RTL8C
ENSG00000104635	23516	SLC39A14
ENSG00000255101	NA	NA

Ensembl ID	Entrez ID	Gene Symbol
ENSG00000147485	137902	PXDNL
ENSG00000254006	NA	NA
ENSG00000169085	254778	C8orf46
ENSG00000245910	641638	SNHG6
ENSG00000040341	27067	STAU2
ENSG00000104435	11075	STMN2
ENSG00000176623	51115	RMDN1
ENSG00000254219	NA	NA
ENSG00000169439	6383	SDC2
ENSG00000132561	4147	MATN2
ENSG00000156482	6156	RPL30
ENSG00000154188	284	ANGPT1
ENSG00000255080	NA	NA
ENSG00000249816	157381	LINC00964
ENSG00000153310	51571	FAM49B
ENSG00000107020	55848	PLGRKT
ENSG00000107165	7306	TYRP1
ENSG00000107186	8777	MPDZ
ENSG00000044459	54875	CNTLN
ENSG00000230453	441459	ANKRD18B
ENSG00000225937	50652	PCA3
ENSG00000165119	3190	HNRNPK
ENSG00000235819	NA	NA
ENSG00000185963	23299	BICD2
ENSG00000131669	4814	NINJ1
ENSG00000130956	22927	HABP4
ENSG00000270332	101928550	SMC2-AS1
ENSG00000119411	54836	BSPRY
ENSG00000106780	1955	MEGF9
ENSG00000136942	11224	RPL35
ENSG00000136935	2800	GOLGA1
ENSG00000148358	57720	GPR107
ENSG00000165699	7248	TSC1
ENSG00000235106	266655	BRD3OS
ENSG00000122140	51116	MRPS2
ENSG00000184925	286256	LCN12
ENSG00000231298	100216001	MANCR
ENSG00000078114	10529	NEBL
ENSG00000229932	NA	NA
ENSG00000120549	56243	KIAA1217
ENSG00000151033	119180	LYZL2
ENSG00000170759	3799	KIF5B

Ensembl ID	Entrez ID	Gene Symbol
ENSG00000150093	3688	ITGB1
ENSG00000165633	196740	VSTM4
ENSG00000166224	8879	SGPL1
ENSG00000165338	143279	HECTD2
ENSG00000108239	23232	TBC1D12
ENSG00000203867	282996	RBM20
ENSG00000108061	8036	SHOC2
ENSG00000148908	6001	RGS10
ENSG00000176769	256536	TCERG1L
ENSG00000051009	84067	FAM160A2
ENSG00000110169	3263	HPX
ENSG00000183378	341277	OVCH2
ENSG00000244398	NA	NA
ENSG00000166833	89797	NAV2
ENSG00000247151	338739	CSTF3-DT
ENSG00000135374	2001	ELF5
ENSG00000175274	9537	TP53I11
ENSG00000066336	6688	SPI1
ENSG00000255197	NA	NA
ENSG00000149115	85456	TNKS1BP1
ENSG00000213593	51075	TMX2
ENSG00000134827	6947	TCN1
ENSG00000149534	2206	MS4A2
ENSG00000254952	NA	NA
ENSG00000110079	51338	MS4A4A
ENSG00000124942	79026	AHNAK
ENSG00000162191	51035	UBXN1
ENSG00000110047	10938	EHD1
ENSG00000168070	283129	MAJIN
ENSG00000175550	10589	DRAP1
ENSG00000213402	5790	PTPRCAP
ENSG00000069482	51083	GAL
ENSG00000255980	NA	NA
ENSG00000131626	8500	PPFIA1
ENSG00000110237	9828	ARHGEF17
ENSG00000215504	NA	NA
ENSG00000204403	100506742	CASP12
ENSG00000137752	834	CASP1
ENSG00000166736	3359	HTR3A
ENSG00000177103	57453	DSCAML1
ENSG00000110324	3587	IL10RA
ENSG00000095139	372	ARCN1

Ensembl ID	Entrez ID	Gene Symbol
ENSG00000149564	90952	ESAM
ENSG00000254612	NA	NA
ENSG00000084234	334	APLP2
ENSG00000149418	6768	ST14
ENSG00000183715	4978	OPCML
ENSG00000177406	100049716	LOC100049716
ENSG00000118971	894	CCND2
ENSG00000237248	100499405	LINC00987
ENSG00000256660	387837	CLEC12B
ENSG00000139151	89869	PLCZ1
ENSG00000123095	79365	BHLHE41
ENSG00000064115	51768	TM7SF3
ENSG00000110841	8496	PPFIBP1
ENSG00000257176	100506606	LOC100506606
ENSG00000079337	10411	RAPGEF3
ENSG00000050438	9498	SLC4A8
ENSG00000123358	3164	NR4A1
ENSG00000135486	3178	HNRNPA1
ENSG00000111481	22818	COPZ1
ENSG00000065361	2065	ERBB3
ENSG00000196531	4666	NACA
ENSG00000166987	114785	MBD6
ENSG00000213352	NA	NA
ENSG00000156076	11197	WIF1
ENSG00000187109	4673	NAP1L1
ENSG00000257345	NA	NA
ENSG00000139344	144193	AMDHD1
ENSG00000075089	64431	ACTR6
ENSG00000196091	4604	MYBPC1
ENSG00000166598	7184	HSP90B1
ENSG00000111199	59341	TRPV4
ENSG00000135111	6926	TBX3
ENSG00000111412	79794	C12orf49
ENSG00000157837	121665	SPPL3
ENSG00000206192	NA	NA
ENSG00000132964	1024	CDK8
ENSG00000133083	9201	DCLK1
ENSG00000133103	57511	COG6
ENSG00000120658	55068	ENOX1
ENSG00000136110	11061	CNMD
ENSG00000187498	1282	COL4A1
ENSG00000088448	55608	ANKRD10

Ensembl ID	Entrez ID	Gene Symbol
ENSG00000130177	8881	CDC16
ENSG00000129566	7011	TEP1
ENSG00000211786	NA	NA
ENSG00000100439	63874	ABHD4
ENSG00000092067	1053	CEBPE
ENSG00000129473	599	BCL2L2
ENSG00000258643	100529063	BCL2L2-PABPN1
ENSG00000129515	58533	SNX6
ENSG00000119688	5826	ABCD4
ENSG00000119684	27030	MLH3
ENSG00000170345	2353	FOS
ENSG00000089916	55668	GPATCH2L
ENSG00000258819	102724190	LINC02289
ENSG00000021645	9369	NRXN3
ENSG00000211896	NA	NA
ENSG00000211955	NA	NA
ENSG00000225698	NA	NA
ENSG00000215252	440270	GOLGA8B
ENSG00000104081	90427	BMF
ENSG00000166145	6692	SPINT1
ENSG00000104147	11339	OIP5
ENSG00000067369	7158	TP53BP1
ENSG00000137857	53905	DUOX1
ENSG00000157456	9133	CCNB2
ENSG00000137818	6176	RPLP1
ENSG00000187806	338949	TMEM202
ENSG00000167202	23102	TBC1D2B
ENSG00000185787	10933	MORF4L1
ENSG00000058335	5923	RASGRF1
ENSG00000172183	3669	ISG20
ENSG00000140545	4240	MFGE8
ENSG00000140526	11057	ABHD2
ENSG00000185033	10509	SEMA4B
ENSG00000176463	28232	SLCO3A1
ENSG00000007541	9091	PIGQ
ENSG00000260260	100507303	SNHG19
ENSG00000008517	9235	IL32
ENSG00000140623	124404	SEPT12
ENSG00000262097	101927311	LINC02185
ENSG00000087253	54947	LPCAT2
ENSG00000159708	55282	LRRC36
ENSG00000124067	6560	SLC12A4

Ensembl ID	Entrez ID	Gene Symbol
ENSG00000182810	55794	DDX28
ENSG00000214353	100130894	VAC14-AS1
ENSG00000166816	197257	LDHD
ENSG00000135697	53630	BCO1
ENSG00000103196	83716	CRISPLD2
ENSG00000197912	6687	SPG7
ENSG00000141503	50488	MINK1
ENSG00000181885	1366	CLDN7
ENSG00000170315	7314	UBB
ENSG00000072210	224	ALDH3A2
ENSG00000128487	92521	SPECC1
ENSG00000226521	NA	NA
ENSG00000267671	NA	NA
ENSG00000161654	124801	LSM12
ENSG00000168591	79089	TMUB2
ENSG00000108821	1277	COL1A1
ENSG00000153930	162282	ANKFN1
ENSG00000062716	81671	VMP1
ENSG00000108506	57508	INTS2
ENSG00000267361	NA	NA
ENSG00000267123	101928710	LINC02081
ENSG00000108679	3959	LGALS3BP
ENSG00000171298	2548	GAA
ENSG00000183010	5831	PYCR1
ENSG00000132874	8170	SLC14A2
ENSG00000167306	4645	MYO5B
ENSG00000267013	101927229	LINC01929
ENSG00000267390	NA	NA
ENSG00000221887	284293	HMSD
ENSG00000125841	80023	NRSN2
ENSG00000088888	57506	MAVS
ENSG00000101265	9770	RASSF2
ENSG00000125968	3397	ID1
ENSG00000101421	128866	CHMP4B
ENSG00000196756	388796	SNHG17
ENSG00000101076	3172	HNF4A
ENSG00000124145	6385	SDC4
ENSG00000177410	441951	ZFAS1
ENSG00000232442	100505771	MHENCR
ENSG00000203880	55251	PCMTD2
ENSG00000197561	1991	ELANE
ENSG00000130270	148229	ATP8B3

**Ensembl ID**	**Entrez ID**	**Gene Symbol**
ENSG00000179855	126326	GIPC3
ENSG00000105278	23217	ZFR2
ENSG00000197256	25959	KANK2
ENSG00000105518	374882	TMEM205
ENSG00000198429	7620	ZNF69
ENSG00000074181	4854	NOTCH3
ENSG00000130520	25804	LSM4
ENSG00000256229	90649	ZNF486
ENSG00000105707	3249	HPN
ENSG00000181666	284459	HKR1
ENSG00000167644	64073	C19orf33
ENSG00000128626	6183	MRPS12
ENSG00000105372	6223	RPS19
ENSG00000187244	4059	BCAM
ENSG00000125740	2354	FOSB
ENSG00000090372	29888	STRN4
ENSG00000118156	84215	ZNF541
ENSG00000105398	6822	SULT2A1
ENSG00000063177	6141	RPL18
ENSG00000104951	259307	IL4I1
ENSG00000171049	2358	FPR2
ENSG00000189068	284415	VSTM1
ENSG00000248385	441864	TARM1
ENSG00000189013	3805	KIR2DL4
ENSG00000108107	6158	RPL28
ENSG00000268205	NA	NA
ENSG00000129824	6192	RPS4Y1
ENSG00000154620	9087	TMSB4Y
ENSG00000215580	286554	BCORP1
ENSG00000131100	529	ATP6V1E1
ENSG00000099889	421	ARVCF
ENSG00000099901	5902	RANBP1
ENSG00000161133	373856	USP41
ENSG00000223553	NA	NA
ENSG00000224144	NA	NA
ENSG00000099998	2687	GGT5
ENSG00000226471	NA	NA
ENSG00000133488	284904	SEC14L4
ENSG00000128311	7263	TST
ENSG00000100092	23616	SH3BP1
ENSG00000100206	11144	DMC1
ENSG00000100316	6122	RPL3

Ensembl ID	Entrez ID	Gene Symbol
ENSG00000100416	55687	TRMU
ENSG00000154654	4685	NCAM2
ENSG00000142192	351	APP
ENSG00000237945	100506334	LINC00649
ENSG00000185917	54093	SETD4
ENSG00000177398	89766	UMODL1
ENSG00000160207	11077	HSF2BP

Table S5. Predicted probabilities for multiclass classification.

Training Set (LOOCV)					Internal validation set				
Training set sample index	Probability of normal sample	Probability of benign disease	Probability of CRC	True label	Internal validation set sample index	Probability of normal sample	Probability of benign disease	Probability of CRC	True label
1	0.007	0.287	0.706	Malignant	1	0.046	0.052	0.902	Malignant
2	0.004	0.831	0.164	Benign	2	0.006	0.908	0.086	Benign
3	0.004	0.039	0.957	Malignant	3	0.013	0.186	0.801	Malignant
4	0.031	0.136	0.833	Benign	4	0.014	0.841	0.146	Benign
5	0.081	0.432	0.487	Benign	5	0.053	0.573	0.374	Benign
6	0.017	0.826	0.158	Benign	6	0.013	0.462	0.525	Benign
7	0.034	0.567	0.400	Malignant	7	0.016	0.331	0.653	Malignant
8	0.027	0.602	0.371	Benign	8	0.003	0.735	0.262	Benign
9	0.017	0.341	0.642	Benign	9	0.004	0.803	0.193	Benign
10	0.028	0.385	0.587	Benign	10	0.013	0.450	0.537	Malignant
11	0.014	0.488	0.498	Benign	11	0.022	0.503	0.475	Benign
12	0.031	0.845	0.124	Benign	12	0.022	0.254	0.724	Malignant
13	0.004	0.850	0.146	Benign	13	0.007	0.574	0.419	Benign
14	0.014	0.005	0.981	Malignant	14	0.021	0.316	0.663	Malignant
15	0.004	0.834	0.162	Benign	15	0.021	0.525	0.454	Benign
16	0.009	0.913	0.078	Benign	16	0.017	0.032	0.951	Malignant
17	0.007	0.954	0.039	Benign	17	0.008	0.400	0.592	Benign
18	0.032	0.777	0.191	Benign	18	0.006	0.652	0.342	Malignant
19	0.047	0.797	0.156	Normal	19	0.026	0.675	0.299	Benign
20	0.041	0.702	0.257	Normal	20	0.029	0.465	0.505	Benign
21	0.010	0.944	0.046	Benign	21	0.004	0.902	0.094	Benign
22	0.016	0.936	0.048	Benign	22	0.010	0.382	0.608	Benign
23	0.024	0.614	0.363	Benign	23	0.029	0.159	0.812	Malignant
24	0.028	0.953	0.019	Benign	24	0.010	0.477	0.513	Malignant
25	0.033	0.945	0.022	Benign	25	0.003	0.425	0.572	Malignant
26	0.042	0.837	0.121	Benign	26	0.006	0.402	0.592	Benign
27	0.012	0.948	0.040	Benign	27	0.005	0.001	0.994	Malignant
28	0.008	0.969	0.023	Benign	28	0.018	0.127	0.855	Malignant
29	0.023	0.915	0.063	Benign	29	0.006	0.939	0.055	Benign
30	0.017	0.822	0.161	Benign	30	0.004	0.965	0.031	Benign
31	0.045	0.470	0.484	Benign	31	0.079	0.020	0.901	Malignant
32	0.004	0.939	0.057	Benign	32	0.004	0.954	0.042	Benign
33	0.020	0.591	0.389	Benign	33	0.004	0.012	0.984	Malignant
34	0.012	0.952	0.037	Benign	34	0.018	0.841	0.141	Malignant
35	0.033	0.541	0.427	Malignant	35	0.011	0.592	0.397	Benign
36	0.014	0.674	0.312	Benign	36	0.004	0.602	0.394	Benign

Training Set (LOOCV)					Internal validation set				
37	0.009	0.782	0.209	Benign	37	0.005	0.550	0.445	Malignant
38	0.011	0.868	0.120	Benign	38	0.007	0.868	0.125	Benign
39	0.004	0.973	0.023	Benign	39	0.019	0.594	0.387	Benign
40	0.036	0.840	0.124	Benign	40	0.017	0.652	0.331	Benign
41	0.012	0.176	0.812	Benign	41	0.005	0.951	0.044	Benign
42	0.021	0.129	0.851	Malignant	42	0.014	0.066	0.921	Malignant
43	0.009	0.528	0.463	Benign	43	0.028	0.097	0.875	Malignant
44	0.013	0.016	0.971	Malignant	44	0.006	0.073	0.921	Malignant
45	0.036	0.610	0.354	Benign	45	0.014	0.904	0.082	Benign
46	0.014	0.056	0.930	Malignant	46	0.015	0.012	0.973	Malignant
47	0.006	0.940	0.054	Benign	47	0.029	0.283	0.688	Benign
48	0.027	0.296	0.677	Malignant	48	0.030	0.538	0.433	Benign
49	0.058	0.457	0.484	Malignant	49	0.005	0.002	0.993	Malignant
50	0.103	0.349	0.548	Malignant	50	0.027	0.543	0.430	Malignant
51	0.085	0.812	0.104	Benign	51	0.018	0.485	0.497	Benign
52	0.022	0.817	0.161	Benign	52	0.094	0.232	0.675	Benign
53	0.024	0.330	0.645	Benign	53	0.008	0.944	0.048	Benign
54	0.011	0.941	0.048	Benign	54	0.022	0.939	0.039	Benign
55	0.020	0.566	0.414	Benign	55	0.016	0.930	0.053	Benign
56	0.017	0.284	0.699	Malignant	56	0.032	0.065	0.903	Malignant
57	0.019	0.704	0.277	Benign	57	0.011	0.174	0.815	Benign
58	0.008	0.697	0.296	Benign	58	0.011	0.905	0.083	Benign
59	0.005	0.974	0.021	Benign	59	0.025	0.491	0.484	Benign
60	0.010	0.017	0.973	Malignant	60	0.007	0.750	0.243	Benign
61	0.005	0.228	0.767	Malignant	61	0.006	0.117	0.877	Malignant
62	0.016	0.728	0.257	Benign	62	0.033	0.436	0.531	Malignant
63	0.028	0.174	0.798	Benign	63	0.006	0.975	0.019	Benign
64	0.025	0.458	0.517	Malignant	64	0.044	0.162	0.794	Malignant
65	0.025	0.430	0.545	Malignant	65	0.016	0.337	0.647	Benign
66	0.006	0.249	0.745	Malignant	66	0.020	0.239	0.741	Malignant
67	0.004	0.681	0.315	Benign	67	0.012	0.077	0.911	Malignant
68	0.028	0.093	0.879	Malignant	68	0.010	0.207	0.782	Malignant
69	0.005	0.498	0.497	Malignant	69	0.128	0.151	0.721	Benign
70	0.020	0.100	0.880	Malignant	70	0.037	0.384	0.578	Benign
71	0.018	0.015	0.967	Malignant	71	0.002	0.711	0.287	Malignant
72	0.028	0.671	0.301	Benign	72	0.002	0.824	0.174	Benign
73	0.009	0.105	0.886	Malignant	73	0.025	0.329	0.647	Benign
74	0.023	0.262	0.716	Malignant	74	0.034	0.110	0.857	Malignant
75	0.007	0.240	0.753	Malignant	75	0.022	0.643	0.335	Benign
76	0.013	0.330	0.657	Malignant	76	0.019	0.249	0.732	Benign
77	0.010	0.802	0.189	Benign	77	0.033	0.434	0.532	Malignant

Training Set (LOOCV)					Internal validation set				
78	0.024	0.362	0.614	Benign	78	0.026	0.261	0.713	Malignant
79	0.007	0.864	0.129	Benign	79	0.003	0.910	0.088	Benign
80	0.003	0.972	0.026	Benign	80	0.988	0.003	0.009	Normal
81	0.025	0.128	0.848	Malignant	81	0.018	0.081	0.901	Malignant
82	0.024	0.352	0.624	Malignant	82	0.003	0.970	0.027	Benign
83	0.005	0.335	0.660	Malignant	83	0.022	0.224	0.754	Malignant
84	0.008	0.976	0.016	Benign	84	0.018	0.746	0.235	Benign
85	0.016	0.084	0.900	Malignant	85	0.021	0.469	0.510	Benign
86	0.031	0.759	0.210	Benign	86	0.010	0.835	0.155	Malignant
87	0.009	0.078	0.913	Malignant	87	0.899	0.042	0.060	Normal
88	0.006	0.931	0.063	Benign	88	0.023	0.399	0.578	Malignant
89	0.017	0.376	0.607	Malignant	89	0.013	0.623	0.365	Benign
90	0.009	0.753	0.238	Malignant	90	0.065	0.799	0.136	Normal
91	0.012	0.198	0.790	Malignant	91	0.018	0.269	0.713	Malignant
92	0.015	0.574	0.412	Benign	92	0.033	0.307	0.661	Malignant
93	0.048	0.470	0.482	Malignant	93	0.016	0.041	0.943	Malignant
94	0.020	0.028	0.952	Malignant	94	0.009	0.931	0.060	Benign
95	0.036	0.276	0.689	Malignant	95	0.035	0.027	0.938	Malignant
96	0.023	0.216	0.761	Malignant	96	0.029	0.365	0.606	Benign
97	0.010	0.753	0.237	Benign	97	0.022	0.851	0.127	Benign
98	0.014	0.743	0.243	Benign	98	0.024	0.006	0.971	Malignant
99	0.069	0.080	0.851	Malignant	99	0.076	0.715	0.209	Benign
100	0.023	0.558	0.419	Benign	100	0.024	0.249	0.727	Malignant
101	0.003	0.909	0.088	Benign	101	0.003	0.984	0.012	Benign
102	0.020	0.399	0.580	Benign	102	0.004	0.873	0.123	Malignant
103	0.004	0.898	0.098	Benign	103	0.014	0.748	0.237	Benign
104	0.008	0.925	0.068	Benign	104	0.003	0.937	0.060	Benign
105	0.010	0.331	0.659	Malignant	105	0.025	0.018	0.957	Malignant
106	0.019	0.865	0.116	Benign	106	0.016	0.729	0.255	Benign
107	0.005	0.807	0.188	Benign	107	0.809	0.101	0.090	Normal
108	0.007	0.281	0.712	Malignant	108	0.027	0.097	0.876	Malignant
109	0.012	0.526	0.461	Malignant	109	0.035	0.113	0.852	Benign
110	0.005	0.916	0.079	Benign	110	0.038	0.238	0.725	Malignant
111	0.021	0.677	0.302	Benign	111	0.024	0.340	0.636	Benign
112	0.006	0.145	0.849	Malignant	112	0.024	0.448	0.528	Benign
113	0.008	0.854	0.138	Benign	113	0.023	0.541	0.436	Benign
114	0.018	0.365	0.617	Malignant	114	0.015	0.804	0.181	Benign
115	0.019	0.942	0.039	Benign	115	0.025	0.245	0.730	Malignant
116	0.022	0.211	0.767	Malignant	116	0.003	0.934	0.063	Malignant
117	0.003	0.974	0.023	Benign	117	0.015	0.695	0.290	Benign
118	0.007	0.779	0.215	Benign	118	0.024	0.331	0.645	Benign

Training Set (LOOCV)					Internal validation set				
119	0.012	0.798	0.191	Malignant	119	0.025	0.139	0.836	Malignant
120	0.019	0.698	0.283	Malignant	120	0.010	0.234	0.756	Malignant
121	0.018	0.811	0.171	Benign					
122	0.018	0.454	0.527	Normal					
123	0.014	0.670	0.316	Malignant					
124	0.004	0.028	0.968	Malignant					
125	0.023	0.100	0.877	Malignant					
126	0.011	0.554	0.435	Benign					
127	0.003	0.920	0.077	Benign					
128	0.023	0.079	0.898	Malignant					
129	0.019	0.676	0.305	Benign					
130	0.008	0.946	0.046	Benign					
131	0.003	0.794	0.203	Benign					
132	0.008	0.745	0.247	Malignant					
133	0.006	0.474	0.519	Benign					
134	0.004	0.937	0.059	Benign					
135	0.004	0.916	0.081	Malignant					
136	0.044	0.266	0.690	Malignant					
137	0.013	0.330	0.657	Malignant					
138	0.077	0.271	0.653	Malignant					
139	0.004	0.955	0.041	Benign					
140	0.005	0.612	0.383	Malignant					
141	0.006	0.265	0.729	Malignant					
142	0.074	0.877	0.050	Benign					
143	0.013	0.007	0.981	Malignant					
144	0.021	0.852	0.127	Benign					
145	0.014	0.900	0.086	Benign					
146	0.054	0.597	0.349	Benign					
147	0.011	0.586	0.403	Benign					
148	0.038	0.800	0.162	Benign					
149	0.013	0.101	0.886	Malignant					
150	0.023	0.089	0.888	Malignant					
151	0.028	0.295	0.676	Malignant					
152	0.012	0.759	0.229	Benign					
153	0.022	0.693	0.285	Benign					
154	0.029	0.934	0.036	Benign					
155	0.022	0.397	0.580	Benign					
156	0.030	0.528	0.442	Malignant					
157	0.018	0.933	0.049	Benign					
158	0.029	0.042	0.929	Benign					
159	0.029	0.009	0.962	Malignant					

Training Set (LOOCV)					Internal validation set				
160	0.006	0.794	0.200	Benign					
161	0.019	0.581	0.400	Benign					
162	0.038	0.314	0.648	Malignant					
163	0.015	0.169	0.816	Benign					
164	0.020	0.836	0.144	Benign					
165	0.022	0.861	0.117	Benign					
166	0.017	0.112	0.872	Malignant					
167	0.016	0.027	0.956	Benign					
168	0.011	0.682	0.308	Malignant					
169	0.016	0.634	0.350	Malignant					
170	0.028	0.024	0.948	Malignant					
171	0.023	0.457	0.521	Malignant					
172	0.015	0.868	0.117	Benign					
173	0.023	0.340	0.638	Benign					
174	0.011	0.175	0.814	Malignant					
175	0.018	0.007	0.975	Malignant					
176	0.018	0.056	0.926	Malignant					
177	0.963	0.016	0.022	Normal					
178	0.755	0.096	0.149	Normal					
179	0.951	0.022	0.027	Normal					
180	0.769	0.126	0.106	Normal					
181	0.984	0.008	0.008	Normal					
182	0.973	0.017	0.010	Normal					
183	0.965	0.022	0.013	Normal					
184	0.976	0.013	0.010	Normal					
185	0.864	0.033	0.103	Normal					
186	0.970	0.012	0.018	Normal					
187	0.989	0.006	0.006	Normal					
188	0.861	0.093	0.045	Normal					
189	0.955	0.020	0.026	Normal					
190	0.986	0.005	0.010	Normal					
191	0.027	0.689	0.285	Benign					
192	0.014	0.874	0.112	Benign					
193	0.027	0.605	0.368	Malignant					
194	0.032	0.860	0.108	Benign					
195	0.017	0.520	0.463	Malignant					
196	0.025	0.399	0.576	Malignant					
197	0.013	0.157	0.830	Malignant					
198	0.019	0.397	0.585	Malignant					
199	0.015	0.061	0.925	Malignant					
200	0.023	0.375	0.602	Malignant					

Training Set (LOOCV)					Internal validation set				
201	0.007	0.358	0.635	Malignant					
202	0.007	0.766	0.226	Benign					
